# Biosynthesis of the bacterial antibiotic 3,7-dihydroxytropolone through enzymatic salvaging of catabolic shunt products†

**DOI:** 10.1039/d4sc01715c

**Published:** 2024-04-24

**Authors:** Lars Höing, Sven T. Sowa, Marina Toplak, Jakob K. Reinhardt, Roman Jakob, Timm Maier, Markus A. Lill, Robin Teufel

**Affiliations:** a Pharmaceutical Biology, Department of Pharmaceutical Sciences, University of Basel Klingelbergstrasse 50 4056 Basel Switzerland robin.teufel@unibas.ch; b Hilde-Mangold-Haus (CIBSS), University of Freiburg Habsburgerstrasse 49 79104 Freiburg im Breisgau Germany; c Biozentrum, University of Basel Spitalstrasse 41 4056 Basel Switzerland; d Computational Pharmacy, Department of Pharmaceutical Sciences, University of Basel Klingelbergstrasse 50 4056 Basel Switzerland

## Abstract

The non-benzenoid aromatic tropone ring is a structural motif of numerous microbial and plant natural products with potent bioactivities. In bacteria, tropone biosynthesis involves early steps of the widespread CoA-dependent phenylacetic acid (paa) catabolon, from which a shunt product is sequestered and surprisingly further utilized as a universal precursor for structurally and functionally diverse tropone derivatives such as tropodithietic acid or (hydroxy)tropolones. Here, we elucidate the biosynthesis of the antibiotic 3,7-dihydroxytropolone in Actinobacteria by *in vitro* pathway reconstitution using paa catabolic enzymes as well as dedicated downstream tailoring enzymes, including a thioesterase (TrlF) and two flavoprotein monooxygenases (TrlCD and TrlE). We furthermore mechanistically and structurally characterize the multifunctional key enzyme TrlE, which mediates an unanticipated *ipso*-substitution involving a hydroxylation and subsequent decarboxylation of the CoA-freed side chain, followed by ring oxidation to afford tropolone. This study showcases a remarkably efficient strategy for 3,7-dihydroxytropolone biosynthesis and illuminates the functions of the involved biosynthetic enzymes.

## Abstract 2

The biosynthesis of the bacterial antibiotic dihydroxytropolone was reconstituted *in vitro* starting from a catabolic shunt product. The involved key flavoprotein monooxygenase TrlE was further structurally and mechanistically characterized.

## Graphical abstract

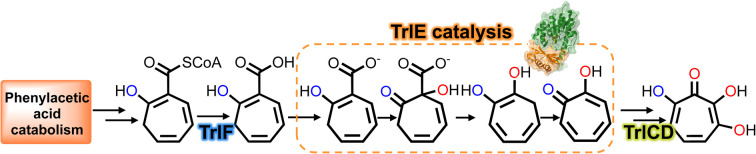

## Introduction

**Fig. 1:**
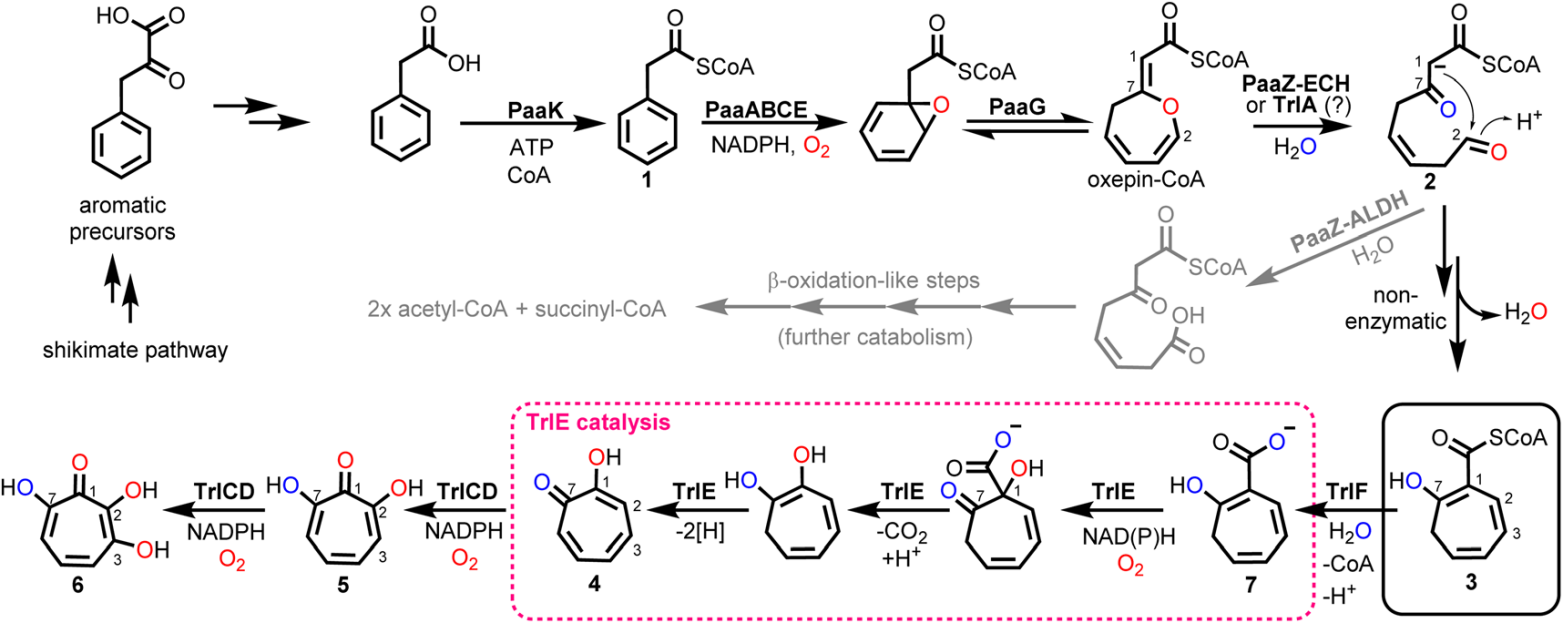
Bacterial *paa* catabolic pathway and biosynthesis of 6 as elucidated herein. Catabolic steps produce reactive 2, which is either converted into central metabolites (grey arrows; catabolic route) or spontaneously cyclizes to the tropone-precursor 3 (black arrows; biosynthetic route). In *Streptomyces* spp., 3 is further converted into 6 involving pathway-specific enzymes encoded by the *trl* gene cluster, with FPMO TrlE (red dashed box) as key enzyme (see text for details). Oxygen atoms shown in red and blue indicate incorporation from O_2_ and H_2_O, respectively. Note that the carbon numbering for all compounds is according to compound 6.

Tropone (2,4,6-cycloheptatrien-1-one) natural products have potent bioactivities against cancer cells, microorganisms and viruses and are generated by fungi, plants and bacteria *via* unrelated biosynthetic pathways. In bacteria, tropone formation was previously shown to surprisingly depend on a catabolic pathway that normally enables aerobic growth on various aromatic compounds such as styrene, 2-phenylethylamine or phenylalanine.^1^ Typically, these aromatics get converted into phenylacetic acid (paa), which is then further degraded into the central metabolites succinyl-CoA and acetyl-CoA by the widespread pathway (Fig. 1).^2–4^ This process first involves the activation of paa by phenylacetyl-CoA synthetase (PaaK; encoded, *e.g.*, by the *paa* gene cluster in *Escherichia coli* K12) to phenylacetyl-CoA (1), followed by the epoxidation of the phenyl ring by a diiron-dependent multicomponent monooxygenase (PaaABCE)^5,6^ and the isomerization to oxepin-CoA by an enoyl-CoA isomerase (PaaG).^7^ The enol ether moiety of this oxepin ring is subsequently hydrolysed by a bifunctional fusion protein (PaaZ), consisting of an N-terminal aldehyde dehydrogenase (ALDH) and a C-terminal (*R*)-specific (MaoC-like) enoyl-CoA hydratase (ECH) domain.^1^ Following ECH-mediated ring cleavage, the produced highly reactive semialdehyde intermediate 2 is rapidly oxidized to the stable carboxylic acid by the ALDH domain, which is facilitated through substrate channeling,^8^ before further final β-oxidation-like degradation steps.^1,2^ However, in a competing side reaction, 2 may also undergo a rapid non-enzymatic intramolecular Knoevenagel condensation to the shunt product dihydrotropone-2-carboxyl-CoA (2-hydroxycyclohepta-1,4,6-triene-1-carboxyl-CoA; (3)). Accidental formation of this shunt product cannot be entirely prevented by PaaZ, thus necessitating strategies to recover the trapped CoA in the bacteria.^1^ Typically, this is achieved *via* a 3-specific thioesterase (PaaY),^5^ while some bacteria instead use 3 as a precursor for the production of diverse secondary metabolites, which frees CoA in the course of the corresponding downstream biosynthetic steps.^1,9^

Aside from rare tropone-containing aromatic polyketides,^10^ the vast majority of bacterial tropones likely derive from the universal precursor 3.^9^ Examples are tropone derivatives such as the virulence factor tropolone (2-hydroxytropone; 4) from the plant pathogen *Burkholderia* spp.^11–13^ or 7-hydroxytropolone (5) and 3,7-dihydroxytropolone (6) from *Streptomyces neyagawaensis* and *S. tropolofaciens* No. K611-97, respectively.^14–16^ Typically, (di)hydroxylated tropolones have strong cytotoxic, antimicrobial and antiviral activities when three adjacent oxygens are present, which allows chelation of up to two metal ions (*e.g.*, Fe^2+^, Zn^2+^, Cu^2+^, Mg^2+^) and thus enables the (un)competitive inhibition of (bi)metallic enzymes such as the medically relevant inositol monophosphatase, matrix metalloproteases and virulence factors (*e.g.*, anthrax lethal factor or HIV reverse transcriptase).^9^ Compound 5, *e.g.*, was shown to inhibit the initiation of DNA synthesis of the hepatitis B virus^17^ and is furthermore highly cytotoxic against cultured B16 melanoma cells, thereby substantially extending the life span of mice with B16 melanoma.^15^ Recent studies suggest that the cytotoxic effects of tropolones (in multiple myeloma cells) may be due to the alteration of cellular iron availability.^18^ Compound 5 was also shown to be synergistic with aminoglycoside antibiotics *in vitro* when administered against resistant bacteria due to the inhibition of the aminoglycoside-inactivating enzyme 2′′-*O*-adenylyltransferase, likely by chelating two catalytically important Mg^2+^ ions.^9,14,16^ While 5 and 6 were discovered several decades ago, a biosynthetic gene cluster (BGC) was only recently identified for 6 biosynthesis in *S. cyaneofuscatus* Soc7 (Table S1†) and *S. luteogriseus* Slg41, which produce 6 at very high titers compared to the originally reported producer *S. tropolofaciens* No. K611-97.^19^ Activity guided cosmid screening and heterologous expression in *S. coelicolor* M1152 revealed a set of distinct co-localized genes required for formation of 6 in these bacteria, as confirmed by comprehensive gene inactivation experiments.^19^ This identified BGC allows the bacteria to produce 6*via* key intermediate 3, in conjunction with the genes from early paa catabolism. Here we reconstitute 6 biosynthesis *in vitro* using heterologously produced enzymes and unravel unexpected enzyme functionalities, in particular for flavoprotein monooxygenase (FPMO) TrlE, which was mechanistically and structurally investigated and shown to catalyze an unusual reaction sequence comprising an *ipso*-substitution of the carboxyl side chain for a hydroxyl group and a final two-electron ring oxidation.

## Results & discussion

### The 3,7-dihydroxytropolone biosynthetic gene cluster is widespread in actinobacteria

**Fig. 2:**
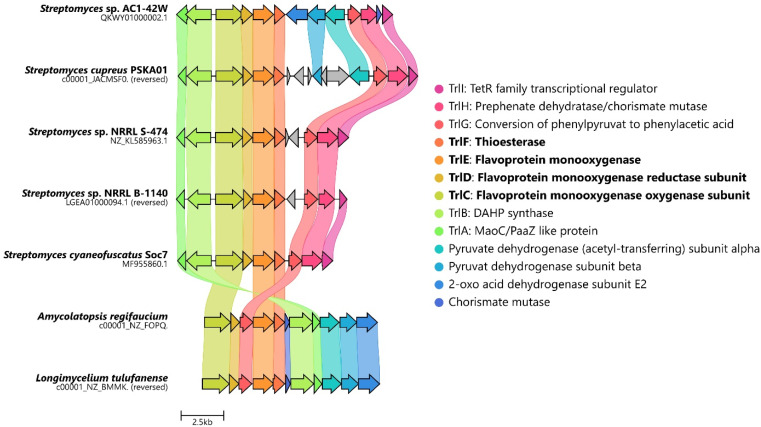
Alignment of the predicted *trl* gene clusters identified in various Actinobacteria. Homologues are highlighted in the same color. Figure created with CAGECAT.^25^

The biosynthesis of 6*via* the *trl* BGC suggested that (hydroxy)tropolones in Actinobacteria are produced from paa-derived 3, which has recently also been shown for Gram-negative *Burkholderia* sp.^12^ and presumably *Pseudomonas* sp.^20^ Accordingly, formation of the virulence factor 4 in the rice pathogen *B. plantarii*^11,13^ involves a functional homologue of flavoprotein TdaE from tropodithietic acid biosynthesis,^9,21–23^ which functions as a highly unusual dioxygenase^12^ and is structurally related to acyl-CoA dehydrogenases (rather than typical oxygenases). TdaE converts 3*via* ring oxidation, CoA-ester oxygenolysis and epoxidation into a highly reactive compound, which spontaneously decarboxylates to 4 (*vide infra*). In contrast, the *trl* BGC encodes seemingly classical FPMOs such as TrlE and TrlCD. To further investigate the occurrence of the *trl* BGC in bacteria, a pBLAST^24^ was performed using TrlE as a query. This allowed the identification of several highly similar BGCs in *Streptomyces* spp., but also other Actinobacteria such as *Amycolatopsis regifaucium* or *Longimycelium tulufanense* from the Pseudonocardiaceae family (Fig. 2), which were both obtained and cultivated. After testing various culture conditions, 5 and 6 could indeed be identified in *L. tulufanense via* LC-MS analysis and based on their distinct UV-vis spectra (Fig. S1 and S2†), whereas *A. regifaucium* apparently lacked tropolone production, showing that the strategy for the biosynthesis of hydroxylated tropolones is widespread in Actinobacteria and possibly dependent on specific environmental influences.

### Enzymatic formation of 3 and the role of putative ECH TrlA

To gain further insights into the underlying enzymology for 6 formation, we first reconstituted oxepin-CoA biosynthesis *in vitro* using chemically synthesized 1 and heterologously produced PaaABCE and PaaG, according to previous studies.^1,2,12^ Based on the homology of TrlA to PaaZ-ECH and the phenotype of the *trlA* deletion mutant,^19^ the same function was expected, that is, hydrolytic cleavage of oxepin-CoA yielding 3. The absence of an ALDH domain would thus result in the complete rerouting of the catabolic pathway to 5 biosynthesis. Surprisingly, however, no conversion of oxepin-CoA was observed upon addition of heterologously produced TrlA under all tested conditions (Fig. S3†). TrlA could be effortlessly produced in *E. coli* Bl21 and purified; moreover, it seemed properly folded and stable. We therefore scrutinized its amino acid sequence and compared it with PaaZ-ECH and related enzymes (Fig. S4†). Important catalytic residues for water activation (H38, D33) and oxyanion hole formation (G56) were conserved, implying full hydratase functionality. We speculate that TrlA may be directly regulated *in vivo* on the enzymatic level by an unknown mechanism, possibly allowing a controlled switch from aromatic catabolism to 5 biosynthesis in *Streptomyces* spp. To investigate downstream 5-biosynthetic enzymes, we thus utilized a PaaZ-E256Q variant with a dysfunctional ALDH domain^1^ to generate 2.

### TrlF is homologous to PaaY and functions as 3-specific thioesterase

We next explored the role of TrlF. Deletion of *trlF* abolished 5 production and furthermore suggested a role for TrlF early in the processing of 3, proposed as an oxidative decarboxylation of the CoA-freed side-chain of a 3-derived intermediate affording tropone.^19^ However, when TrlF was added to the enzyme assays, the direct conversion of 3 into dihydrotropone-2-carboxylate (7) was observed, as verified by UPLC-HRMS analysis and comparison to a chemically synthesized standard (*i.e.* tropone-2-carboxylate, which could be converted into 7 by chemical oxidation, see ESI Fig. S5 and S6†). Tropone, in contrast, was only observed in minor amounts in the assays, arising from the spontaneous decarboxylation and oxidation of 7, similar to previous observations from bacterial mutant strains lacking the ALDH functionality for processing of 2.^26^ When analyzing the protein sequence of TrlF, we noticed that it is homologous to PaaY from paa catabolism, which was previously shown to function as a zinc-dependent thioesterase^5^ with the structural (active site) architecture of γ-carbonic anhydrases^27,28^ (Fig. S7†). PaaY orthologs were confirmed to be homotrimers,^27,28^ whose biological role is to hydrolyze the thioester of shunt product 3 and thus to counteract the depletion of CoA during paa catabolism.^5^ These enzymes contain one central Ca^2+^ and three Zn^2+^ at the interface between the monomers,^27^ which likely have both structural and catalytic roles equivalent to carbonic anhydrases. Interestingly, PaaY from *E. coli* was reported to lack carbonic anhydrase functionality,^28^ as opposed to the respective ortholog from the human pathogen *Acinetobacter baumanii* (for which a functional paa catabolon is important for virulence^27^) that also catalyzed the interconversion of CO_2_ and HCO_3_^−^; additionally, both enzymes differed in their specificity for acyl-CoA-esters.^5,27^ We could observe carbonic anhydrase activity of TrlF from *S. cyaneofuscatus* Soc7 based on a photometric test using the pH indicator dye phenol red in saturated CO_2_ solution with a specific activity of ∼0.57 μmol min^−1^ mg^−1^ (Fig. S8†). The underlying catalytic mechanism of PaaY orthologs and TrlF most likely corresponds to the moonlighting functionality of some carbonic anhydrases that cleave thioesters *via* nucleophilic attack of an active site Zn^2+^-bound hydroxide ion on the carbonyl of the thioester moiety.^29^

### TrlE is a multifunctional key tailoring enzyme

**Fig. 3:**
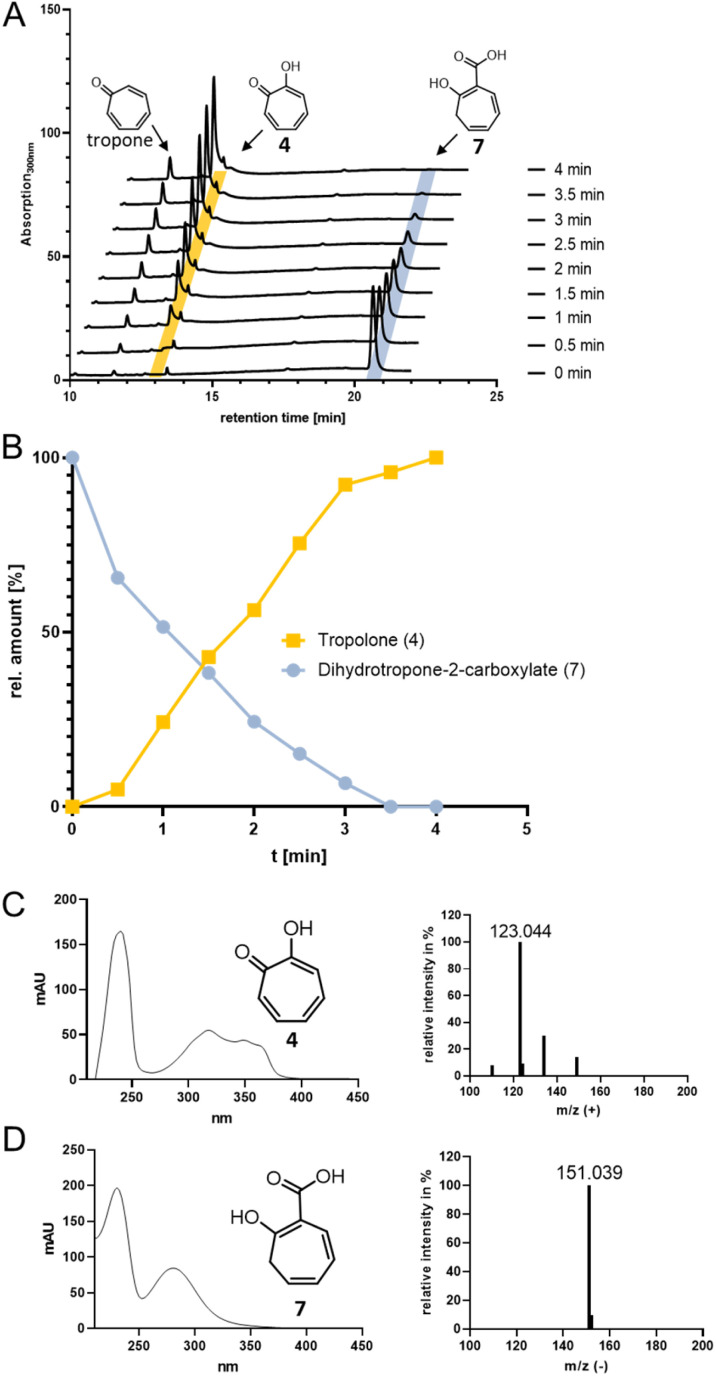
(A) RP-HPLC chromatograms at 300 nm showing the conversion of 7 (highlighted in blue) into 4 (highlighted in yellow) by TrlE. (B) Plotted data from (A) showing relative amounts of 4 and 7 in % (area under the peak) against time. (C) UV-vis and MS spectra of 4 (identical to commercially available 4 standard) and of 7.

For catalysis of the next pathway step presumably involving a ring hydroxylation, the predicted FPMO TrlE seemed a prime candidate. In general, flavoenzymes are specific for flavin mononucleotide (FMN) or flavin adenine dinucleotide (FAD). Heterologously produced and purified TrlE exhibited yellow color and a UV-vis spectrum indicative of bound flavin, which was identified *via* HPLC-DAD as FAD by comparison with a standard (Fig. S9†). The Δ*trlE* mutant strain accumulated mainly tropone, which was accordingly proposed as substrate for the production of 4*via* hydroxylation.^19^ However, TrlE with NADPH could not convert tropone; instead, the enzyme surprisingly accepted the TrlF-product 7 and generated 4 without accumulation of apparent intermediates, as shown by RP-HPLC, LC-MS (Fig. 3) and comparison with a commercially available standard (Fig. S10–S12†). In this reaction, minor amounts of tropone were found, most probably resulting from the spontaneous decomposition of the TrlF-produced 7 (as described above), which could also explain tropone-accumulation in the Δ*trlE* mutant strain.

This unexpected result implied that TrlE catalyzes several steps in 6 biosynthesis, including an *ipso*-substitution initiated by C1-hydroxylation that enables the subsequent side-chain elimination *via* decarboxylation with the ring ketone acting as electron sink, before a final two-electron ring oxidation generates aromatic 4. ^18^O-isotope labelling experiments confirmed the incorporation of O_2_-derived oxygen into the substrate and thus verified TrlE's oxygenase functionality (Fig. S13†). Based on sequence comparison and phylogenetic analysis, TrlE belongs to the group A FPMOs that typically feature a mobile flavin cofactor with distinctive “OUT” and “IN” conformations, enabling flavin reduction (in the presence of the native substrate) and substrate hydroxylation, respectively.^30,31^ Microbial group A FPMOs are widely found in natural product biosynthetic pathways and particularly oxygenate activated substrates, *e.g.*, *via* hydroxylation of phenolic moieties or Baeyer–Villiger oxygenations of ketones.^31–33^ Sequence comparison and a phylogenetic tree (Fig. S14, S15 and Table S2†) revealed that TrlE is closely related to enzymes from catabolic pathways, including salicylate hydroxylases from various *Pseudomonas putida* strains (*e.g.*, SalH^34^ and NahG^35^) that convert salicylate into catechol, 3-hydroxybenzoate 6-hydroxylase (3HB6H) from *Rhodococcus jostii*,^36^ or 6-hydroxynicotinate 3-monooxygenase (NicC) from *Pseudomonas putida* KT2440 and other strains.^37,38^ The only other enzyme involved in natural product biosynthesis is TropB from *Talaromyces stipitatus* catalyzing an oxidative phenol dearomatization in the biosynthesis of the fungal tropolone natural product stipitatic acid (which is biosynthetically unrelated to bacterial tropolones).^39–41^ Interestingly, in this case, the tropolone ring is formed in downstream steps by a non-heme Fe(ii)-dependent dioxygenase TropC *via* an oxidative ring expansion.^40^

**Fig. 4:**
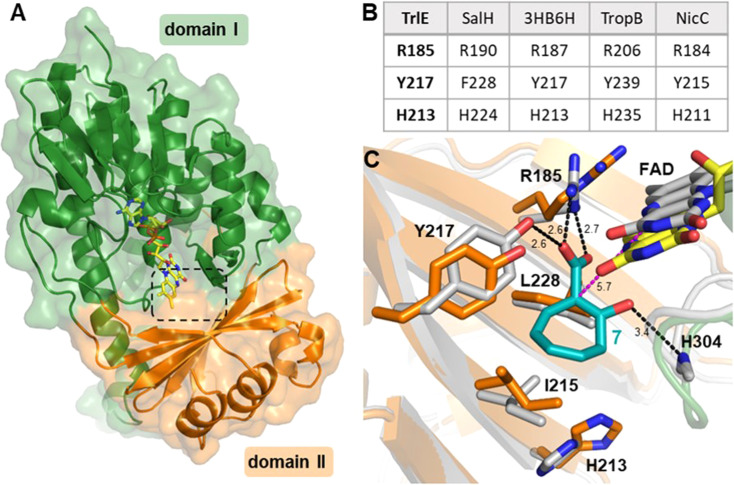
Structure of TrlE with FAD in the IN conformation and predicted binding mode of 7. (A) Overall structure of TrlE comprising the FAD-binding domain (domain I; green; residues 1–71, 105–177 and 278-400) and the substrate binding domain (domain II; orange; residues 72–104 and 178–287). A dashed box indicates the location of the active site. (B) Comparison of corresponding active site residues from structural alignments with SalH (PDB ID: 5EVY), 3-hydroxybenzoate 6-hydroxylase (3HB6H; PDB ID: 4BJZ), TropB (PDB ID: 6NET) and NicC (PDB ID: 5EOW). (C) Predicted binding mode of 7 in the active site of TrlE. The TrlE structure resulting from molecular dynamics simulation (white) is shown aligned to the experimental crystal structure (orange, green, yellow). Potential hydrogen bonds are indicated as black dashes. The distance between C_1_ in 7 to C_4α_ in FAD after molecular dynamics simulation is highlighted with magenta dashes. Distances are shown in Ångström.

To gain more insights into the underlying catalytic mechanism of TrlE, further mechanistic and structural investigation was conducted starting with protein crystallization. Following extensive screening, yellow TrlE crystals indicative of bound FAD could be obtained that diffracted up to 2.5 Å (Table S3†). The structure was solved *via* molecular replacement and showed that TrlE features the typical glutathione reductase (GR-2) type Rossmann fold of group A FPMOs with two distinct domains I and II involved in FAD and substrate binding, respectively (Fig. 4A). The non-covalently bound FAD observed in this structure is present in the “IN” conformation, in which the isoalloxazine moiety faces the proposed substrate binding site.

Interestingly, the active site of TrlE with residues R185, Y217 and H213 most closely resembles TropB, 3HB6H and NicC (rather than SalH/NahG), implying similar mechanisms for substrate binding and activation in these enzymes (Fig. 4B). To further investigate the substrate binding and activation by TrlE, co-crystallization and soaking of the protein crystals with 7 was attempted. While TrlE readily crystallized with bound FAD, no complex structure with the substrate could be obtained. Similar attempts with the substrate analogues tropone-2-carboxylate and salicylate also remained unsuccessful, although binding of these compounds to TrlE was confirmed by differential scanning fluorimetry (DSF) (Fig. S16–S18 and Table S4†).

To further investigate the structural basis for substrate binding and activation, a molecular model was generated for 7 interacting with TrIE. Flexible molecular docking of 7 into the obtained crystal structure of TrIE was conducted. Throughout a subsequent 100 ns molecular dynamics simulation, the position of 7 remained in the proposed substrate binding pocket nearby the isoalloxazine moiety of the FAD (Fig. S19†). Similar binding positions were also observed for the native substrates in the respective crystal structures of SalH, 3HB6H and TropB (Fig. S20†). For our analysis, we chose a representative binding mode of 7 (Fig. 4C).

**Fig. 5:**
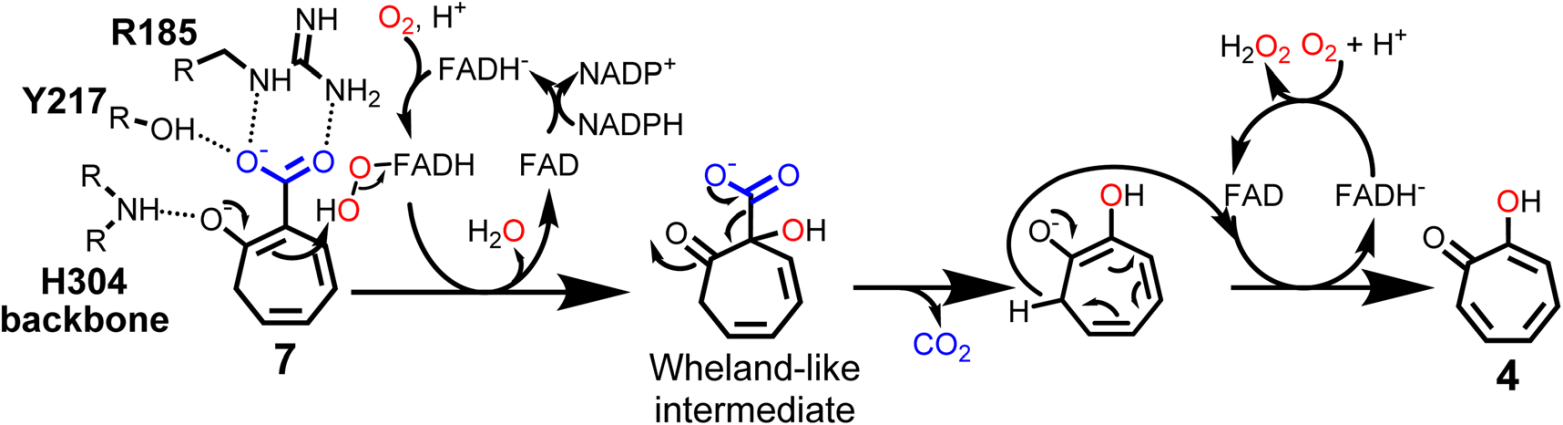
Proposed catalytic mechanism of TrlE. Conversion of 7 into 4*via* flavin-C_4α_-hydroperoxide-mediated hydroxylation, followed by decarboxylation and FAD-dependent ring oxidation.

These results suggest a plausible substrate binding mode with the carboxylate group of 7 forming hydrogen bonds with the side-chains of R185 and Y217, while the ring system packs against a hydrophobic part of the pocket formed by L228 and I215. Compound 7 is ring hydroxylated at C_1_, which is located approximately 5.6 Å from the flavin C_4α_ and thus fully in line with typical substrate orientations in FPMOs that employ the canonical flavin-C_4α_-(hydro)peroxide for catalysis.^30,42,43^ Most likely, to lower the activation barrier for hydroxylation, first the activating adjacent ring hydroxyl group needs to become deprotonated, as similarly described, *e.g.*, for the prototype group A FPMO *para*-hydroxybenzoate hydroxylase.^44^ In the case of TrlE, this hydroxyl group of 7 points upwards towards the re-side of the FAD-C_4α_, adjacent to a loop with the sequence motif PHH (Fig. 4C). In some group A FPMOs, this loop plays an important role in substrate binding and is key for regulating the switch from the flavin “IN” into the “OUT” conformation.^45^ For TrlE, no amino acid side chains point towards the hydroxyl group of 7, however, the backbone nitrogen of H304 from this loop is suitably positioned at 3.4 Å distance (Fig. 4C) to stabilize the negative charge and thus promote an electrophilic attack of the flavin-C_4α_-hydroperoxide onto the C_1_ of 7, generating a Wheland-like intermediate as described for SalH/NahG^35^ (Fig. 5). Subsequently, the carboxylate group of 7, whose negative charge is initially stabilized by the R185 and Y217 side chains, would be pushed towards a hydrophobic part of the pocket lined by L228, which would favor decarboxylation through electrostatic destabilization^35^ and afford 4 as product following a final two-electron ring oxidation. Interestingly, such an oxidation step was not reported for any of the closely related catabolic enzymes that mediate similar *ipso*-substitutions. To investigate whether TrlE actively catalyzes this step or if it results from spontaneous oxidation by O_2_, 4 was chemically reduced with sodium dithionite to obtain the proposed intermediate, before TrlE was added. Re-oxidation was spectroscopically monitored at 340 nm and clearly showed that intact TrlE led to the fastest formation of 4 compared to heat-inactivated enzyme or free FAD (Fig. S21 and S22†).

In SalH/NahG, H226 was proposed to serve as catalytic base; in TrlE, the corresponding H213 is however located approximately 8 Å from the hydroxyl group of 7, which makes a direct involvement in catalysis unlikely. In addition, Y217 is not conserved in these enzymes, instead a phenylalanine is present at the corresponding position. To investigate the role of both amino acids in TrlE catalysis, site directed mutagenesis was employed, generating the mutants H213A, H213E, H213Q and Y217F. Overall, the resulting variants showed reduced FAD loading upon purification and were less heat stable compared to the wild type (Fig. S16A†), while their hydrodynamic behavior during size-exclusion chromatography remained unaffected (Fig. S23†). While H213A lost the ability to produce 4 entirely, the other variants showed strongly reduced catalytic activity, with slowed substrate consumption and product formation compared to the wild-type enzyme (Fig. S24†). In our current model, Y217 contributes to substrate binding, but is not directly involved in catalysis. Therefore, the observed reduced catalytic activity seems plausible. For both H213E and H213Q variants, minor product formation was still observed, which, together with the proposed substrate positioning in the active site, indicates that this residue most likely plays only a subsidiary role in catalysis. The strongly reduced thermal stability in all H213 variants rather implies that this residue is important for the structural integrity of TrlE.

### TrlCD is a two-component FPMO producing 3,7-dihydroxytropolone as main product

The Δ*trlC* mutant strain accumulated 4 as main compound, implying that TrlC may act subsequent to TrlE and likely catalyzes two consecutive hydroxylations of 4*via*5 into 6.^19^ TrlC is predicted to be the monooxygenase component of a two-component FPMO that relies on a separate enzyme, for flavin reduction, here TrlD.^46,47^ As opposed to the corresponding monooxygenase constituents, these flavin reductases (fre) typically have higher affinity for oxidized rather than reduced flavins, thus ensuring efficient reductive and oxidative half reactions.^46,47^ Following the purification of the two separately heterologously produced enzymes TrlC and TrlD, the cofactor specificity of TrlD was analyzed. Accordingly, TrlD preferred FAD and NADH over FMN and NADPH, respectively (Fig. S25†). This is consistent with the observation that a fraction of TrlD was co-purified with bound FAD (Fig. S9†). When TrlCD, FAD and NADH were added together to the *in vitro* enzyme mix, the TrlE-produced 4 was rapidly converted into a new compound (5); analogous results were obtained from separate TrlCD assays without the other enzymes using commercially available 4 (Fig. S26 and S27†). Compound 5 was identified as 7-hydroxytropolone *via* UPLC-HRMS, NMR, and UV spectroscopy, fully consistent with previously published data^14^ (Table S5 and Fig. S28–32†). It is noteworthy that TrlC also generated 5*in vitro* when TrlD was substituted with the flavin reductase from *E. coli*, albeit product formation was slightly diminished (Fig. S33†). Interestingly, in contrast to Δ*trlC*, *trlD* inactivation did not completely abolish product formation, leading to accumulation of 5 rather than 4 in the heterologous producer strain *S. coelicolor* M1152, suggesting that the loss of the flavin reductase TrlD is to some extent complemented by the host strain (presumably by other flavin reductases). Notably, the final product 3,7-dihydroxytropolone (6) was observed in the TrlCD assay after prolonged incubation time and only when an NADH regeneration system (consisting of sodium formate and formate dehydrogenase^48^) was employed, as verified by LC-MS (Fig. S26 and S34–S36†). Although the quantities of 6 obtained *in vitro* did not suffice for NMR measurements, the produced compound matched previously reported physicochemical properties of 6 and also the compound produced by *L. tulufanens* in this work.

**Fig. 6:**
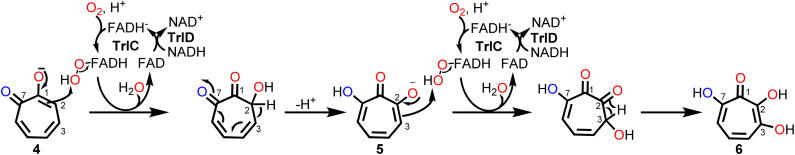
Proposed mechanism for the dual hydroxylation of 4 by the two-component FPMO TrlCD affording 6. See text for details.

Notably, the complete *in vitro* pathway reconstitution of 6 biosynthesis could be achieved, allowing the complete one-pot conversion of 1 into 6 by combining enzymes from early paa catabolism with the specific tropolone tailoring enzymes (Fig. S27†). Interestingly, 6 was only produced in spurious amounts when TrlD was substituted with flavin reductase from *E. coli* under the same conditions, which suggests the requirement of proper protein–protein-interactions between the two native redox partner enzymes for efficient catalysis. For 6 formation, we propose a mechanism involving two consecutive *ortho*-hydroxylations at C2 (activated by the C1-hydroxyl) and the adjacent C3 (enabled by the newly introduced C2-hydroxyl), which would make substrate re-positioning of 5 likely superfluous within the active site of TrlC for the second hydroxylation reaction (Fig. 6). Further insights into the underlying mechanism will require detailed structural and mechanistic studies of TrlCD.

## Conclusion

In summary, for the first time a complete bacterial tropone biosynthetic pathway could be reconstituted *in vitro* using a combination of 3-producing paa catabolic enzymes as well as enzymes specific for the formation of 4, 5 and 6, *i.e.* thioesterase TrlF and the FPMOs TrlE and TrlCD, whose functions could be assigned and investigated in detail. The strategy for formation of hydroxylated tropolones thus relies on an unusual intertwining of enzymes from primary and secondary metabolism. While the biosynthesis of unmodified 4 from 3 in Gram-negative bacteria has been recently linked to the action of the non-canonical flavoprotein dioxygenase TdaE that structurally resembles acyl-CoA dehydrogenases,^12^ herein we describe a structurally and functionally unrelated group A FPMO TrlE from Gram-positive Actinobacteria with GR-2 type Rossmann fold as key enzyme for 4 formation. In contrast to TdaE orthologs, which produce a highly reactive epoxide that is prone to undergo spontaneous decarboxylation, TrlE directly controls side-chain elimination *via* the herein identified *ipso*-substitution. Notably, both enzymes catalyze multiple reactions and combine oxidation reactions that do not involve oxygen-transfer to the substrate (normally facilitated by dedicated flavoprotein oxidases or dehydrogenases) with distinct oxygenation chemistry. While TdaE may rely on non-canonical flavin-N_5_-oxygen adducts for oxygen transfers,^12,49,50^ TrlE most likely employs the flavin-C_4α_-hydroperoxide for hydroxylation and represents a novel multitasking FPMO in natural product biosynthesis that consecutively catalyzes not only hydroxylation and decarboxylation but a two-electron ring oxidation as well. In conjunction with the elucidation of the role of TrlCD, these results underscore the predominant roles of flavoenzymes in the construction and redox tailoring of microbial natural product skeletons such as tropones^32^ and open new possibilities for the bioengineering of tropolone pathways and the generation of novel derivatives.

## Data availability

The X-ray crystallography structure of TrlE was deposited at the RCSB Protein Data Bank (PDB) with the code 8RQH. All further relevant data can be found in this manuscript or the ESI.† Raw data is available at the Zenodo online repository or upon request.

## Author contributions

Lars Höing: investigation, formal analysis, validation, visualization, writing – original draft, Sven T. Sowa: formal analysis, supervision, data curation, writing – review & editing, Marina Toplak: conceptualization, supervision, writing – review & editing, Jakob K. Reinhardt: formal analysis, writing – review & editing, Roman Jakob: formal analysis, data curation, writing – review & editing, Timm Maier: resources, writing – review & editing, Markus A. Lill: investigation, formal analysis, validation, writing – review & editing, Robin Teufel: project administration, conceptualization, supervision, funding acquisition, writing – original draft.

## Conflicts of interest

The authors declare no conflict of interest.

## Supplementary Material

SC-015-D4SC01715C-s001
