# Supplementary material for: Biosynthesis of the bacterial antibiotic 3,7-dihydroxytropolone through enzymatic salvaging of catabolic shunt products
Source: Chem Sci. 2024 Apr 24;15(20):7749–56. doi: 10.1039/d4sc01715c (PMC11110157; doi:10.1039/d4sc01715c)
Supplement: SC-015-D4SC01715C-s001 [file SC-015-D4SC01715C-s001.pdf]

Electronic Supplementary Material (ESI) for Chemical Science.
This journal is © The Royal Society of Chemistry 2024

Supporting Information

Biosynthesis of the Bacterial Antibiotic 3,7-Dihydroxytropolone through Enzymatic Salvaging of Catabolic Shunt Products

Lars Höing[a], Sven T. Sowa[a], Marina Toplak[b], Jakob K. Reinhardt[a], Roman Jakob[c], Timm Maier[c], Markus A. Lill[d] and Robin Teufel[a]*

Table of Contents

1. Experimental Procedures 4

1.1 Materials 4

1.2 Media and Microorganisms 4

1.3 Cloning and recombinant production of TrlA 4

1.4 Protein purification of TrlA 5

1.5 Cloning, recombinant production and protein purification of TrlC 5

1.6 Cloning, recombinant production and protein purification of TrlD 5

1.7 Cloning, recombinant production and protein purification of TrlE 5

1.8 Cloning, recombinant production and protein purification of TrlF 5

1.9 Cloning, recombinant production and protein purification of TrlE Variants 5

1.10 Recombinant production and purification of PaaABCE, PaaG, PaaZ-E256Q and PaaY 6

1.11 Multiple sequence alignment Trl gene cluster 6

1.12 Phylogenetic distance tree TrlE 6

1.13 Analytical size exclusion chromatography (SEC) 6

1.14 Determination of melting temperature (TM) of TrlE and variants 6

1.15 Crystallization of TrlE, data collection and refinement 6

1.16 Docking, molecular dynamics (MD) simulation and energy minimization 6

1.17 Chemical synthesis of phenylacetyl-CoA (PaCoA) (1) 7

1.18 HPLC analysis 7

1.19 LC-HRMS and LC-MS analysis 7

1.20 Enzymatic synthesis of 2 8

1.21 Enzymatic synthesis of 3 8

1.22 Turnover assay with TrlF 8

1.23 Carbonic Anhydrase Test 8

1.24 Turnover assay with TrlE 8

1.25 Tropone turnover assay with TrlE 9

1.26 Turnover assay with TrlCD 9

1.27 Comparison of TrlA and PaaZ-E256Q 9

1.28 Oxepin-CoA assay TrlE 9
1.29 $^{18}O_2$-labeling assays 9
1.30 Differential scanning fluorimetry (DSF) measurements 10
1.31 Re-oxidation of 4 10
1.32 Cultivation of *Longimycelium tulufanense* 10
1.33 NMR measurement of 5 10
2. Supplemental Figures and Tables: 11
Table S1 Trl gene cluster of *Streptomyces cyaneofuscatus* Soc7 11
Figure S1 Observed production of 4, 5, and 6 in *Longimycelium tulufanense* 12
Figure S2 UV-visible spectra of compounds 4, 5 and 6 13
Figure S3 Functional test of putative hydratase TrlA 14
Figure S4 Multiple sequence alignment of TrlA from *Streptomyces cyaneofuscatus* Soc7, PaaZ from *E. coli* K12 and homologue proteins from other organisms 15
Figure S5 Time course of 7 formation by TrlF 16
Figure S6 Oxidation of 7 to tropone-2-carboxylate 17
Figure S7 Multiple sequence alignment of TrlF from *Streptomyces cyaneofuscatus* Soc7 and homologues from other organisms 18
Figure S8 Test for carbonic anhydrase activity of TrlF 19
Figure S9 RP-HPLC analysis (450 nm) of isolated cofactors from TrlE and TrlD 20
Figure S10 NADPH-dependency of the turnover of (TrlF-produced) 7 to tropolone (4) by TrlE 21
Figure S11 Incubation of tropone with TrlE 22
Figure S12 Comparison of tropone and tropolone standards to peaks from assay containing TrlE and TrlF 23
Figure S13 Incorporation of 18O labeled oxygen into tropolone (4) by TrlE 24
Figure S14 Multiple sequence alignment of TrlE with known FAD-dependent group A monooxygenases 25
Figure S15 Phylogenetic distance tree of TrlE and other group A flavoprotein monooxygenases 26
Table S2 TrlE and homologues enzymes from the phylogenetic distance tree with according PDB code, Uniprot Accession Nr. and species 27
Table S3 Data collection and refinement statistics from the crystal structure of TrlE. Statistics for the highest-resolution shell are shown in brackets 28
Figure S16 Determination of the melting temperature (T_m) of TrlE and variants 29
Figure S17 Effect of tropone-2-carboxylic acid (TCA) on the melting temperature (T_m) of TrlE 30
Figure S18 Effect of FAD on the melting temperature (T_m) of TrlE 30
Table S4 Melting temperatures (TM) and standard deviation (SD) in °C of TrlE and its variants 31
Figure S19 Representative docking poses of 7 in the active site of TrlE 32
Figure S20 Comparison of the docked pose of 7 in the active site of TrlE with crystal structures of homologs in complex with their native substrates 33

Figure S21 UV-visible spectra of tropolone (4) without (solid line) and with dithionite (dashed line) in 50 mM Tris-HCl pH 8 buffer 34

Figure S22 TrlE mediated re-oxidation of tropolone........ 35

Figure S23 SEC of TrlE variants 36

Figure S24 RP-HPLC analysis (300nm) of the conversion of 7 (highlighted in blue) into 4 (green) by TrlE WT and different variants 37

Figure S25 Cofactor preferences of TrlD 38

Figure S26 RP-HPLC analysis (300 nm) of 7-hydroxytropolone (5) and 3,7-dihydroxytropolone (6) formation catalyzed by TrlCD over time 39

Figure S27 Complete 3,7-dihydroxytropolone (6) biosynthesis pathway reconstituted 40

Table S5 HRMS-measurements and calculated masses for compounds 4, 5 and 6........ 41

Figure S28 1H NMR spectrum of 5 (500 MHz, C6D6) 42

Figure S29 ^{13}C NMR spectrum of 5 (126 MHz, C_6D_6). 43

Figure S30 COSY NMR spectrum of 5 (500 MHz, C_6D_6). 43

Figure S31 HSQC NMR spectrum of 5 (500 MHz, C_6D_6). 44

Figure S32 HMBC NMR spectrum of 5 (500 MHz, C_6D_6). 44

Figure S33 Comparison of the conversion of 4 into 5 and 6 by TrlCD and TrlC + Fre including cofactor regeneration system 45

Figure S34 Comparison of conversion of tropolone (4) by TrlCD with and without cofactor regeneration system 46

Figure S35 EIC of compounds 4, 5 and 6 obtained during enzymatic assays with TrlCD 47

Figure S36 Overview of structures, methanol solutions and UV spectra of compounds 5 and 6..... 48

Figure S37 SDS-PAGE analysis of different fractions collected during affinity purification (IMAC) of 6xHis-gb1-tagged TrlA 49

Figure S38 SDS-PAGE analysis of different fractions collected during affinity purification (IMAC) of 6xHis-tagged TrlC........ 50

Figure S39 SDS-PAGE analysis of different fractions collected during affinity purification (IMAC) of 6xHis-gb1-tagged TrlD 51

Figure S40 SDS-PAGE analysis of different fractions collected during affinity purification (IMAC) of 6xHis-gb1-tagged TrlE........ 52

Figure S41 SDS-PAGE analysis of different fractions collected during affinity purification (MBP-Trap) of MBP-tagged TrlF........ 53

Figure S42 SDS-PAGE analysis of different fractions collected during affinity purification (IMAC) of 6xHis-gb1-tagged TrlE H213A........ 54

Figure S43 SDS-PAGE analysis of different fractions collected during affinity purification (IMAC) of 6xHis-gb1-tagged TrlE H213E 54

Figure S44 SDS-PAGE analysis of different fractions collected during affinity purification (IMAC) of 6xHis-gb1-tagged TrlE H213Q 55

Figure S45 SDS-PAGE analysis of different fractions collected during affinity purification (IMAC) of 6xHis-gb1-tagged TrlE Y217F........ 55

Figure S46 Analytical SEC of his-gb1 tagged TrlE, TrlA and TrlD, his tagged TrlC and MBP tagged TrlF 56

3. SI References 57

1. Experimental Procedures

1.1 Materials

All chemicals and reagents were obtained from Carl Roth (Karlsruhe, Germany), Sigma-Aldrich (St. Louis, MO, USA), Alfa Aesar (Haverhill, MA, USA), Biomol (Hamburg, Germany) and Fisher Scientific (Hampton, NH, USA). Primers, enzymes and additional material used for molecular cloning were purchased either from New England Biolabs (NEB), Thermo Fisher Scientific or Sigma-Aldrich. Custom synthesized DNA was obtained from Biocat (Heidelberg, Germany) and Twist Bioscience (South San Francisco, CA, USA). DNA sequencing was carried out by Eurofins Genomics (Ebersberg, Germany) and Microsynth (Balgach, Switzerland). For protein purification, equipment (MBP-Trap columns, Ni-NTA-columns and gelfiltration coumns) from Cytiva (Marlborough, MA, USA) was used. Concentration of proteins was carried out in centrifugal devices from Thermo Fisher Scientific and PALL (New York, USA). SDS-PAGE and Agarose gel electrophoresis were carried out in equipment from Cleaver Scientific (Rugby, UK).

1.2 Media and Microorganisms

Since the strain *Streptomyces cyaneofuscatus* Soc7 was unobtainable, 3,7-dihydroxytropolone formation was investigated in two strains with homologous gene clusters, i.e. *Longimycelium tulufanense* DSM 46696 and *Amycolatopsis regifaucium* DSM 45072 (both obtained from the German Collection of Microorganisms and Cell Cultures, DSMZ). The media used to cultivate both strains on plates was GYM Streptomyces medium (D-glucose 4 g/L, yeast extract 4 g/L, malt extract 10 g/L, $CaCO_3$ 2 g/L and agar 12 g/L). For cultivation in liquid media either GYM Streptomyces medium (D-glucose 4 g/L, yeast extract 4 g/L, malt extract 10 g/L and $CaCO_3$ 2 g/L) or GYM-NaCl medium (D-glucose 4 g/L, yeast extract 4 g/L, malt extract 10 g/L, NaCl 4 g/L and $CaCO_3$ 2 g/L) was used. Both strains were cultivated at 140 rpm, *Longimycelium tulufanense* at 37°C and *Amycolatopsis regifaucium* at 28°C.

1.3 Cloning and recombinant production of TrlA

For the recombinant production of TrlA (NCBI accession number: AWF83805.1, see table S1) the corresponding gene was synthesized and codon optimized for *E. coli* by Biocat. Restriction digestion of the gene was carried out with NotI (3') and NcoI (5'), afterwards the gene was cloned into the pET-M11-His-gb1-TEV vector. This vector contained the solubility enhancer protein gb1 (B1 domain of *Streptococcal* protein g) between the hexahistidine-tag and the tobacco etch virus (TEV)-cleavage site at the N-terminus of the protein. After proper insertion into the target vector was confirmed by sanger sequencing, the corresponding plasmid was transformed into *E. coli* BL21 (DE3) pL1SL2 cells obtained from Leadley et al.[1] These cells contain the GroES/GroEL chaperonin system from *Streptomyces coelicolor*, which facilitate correct folding of proteins from G+C rich species. Gene expression was carried out in TB-medium supplemented with kanamycin (50 µg/mL), ampicillin 100 µg/mL), chloramphenicol (20 µg/mL) and D-glucose (0.2% w/v). For inoculation, a pre-culture was grown in LB-medium (supplemented with the same amount of antibiotics and glucose) until an OD_{600} of ~0.1 was reached. The main culture was incubated at 37°C and 130 rpm until an OD_{600} of ~0.6 was reached. Afterwards, the temperature was adjusted to 18°C and protein production was induced with 0.25 mM IPTG. The cells were harvested after overnight incubation at 5.000 x g, washed with 0.9 % NaCl solution (w/v), centrifuged again at 5.000 x g and then frozen at -20°C.

1.4 Protein purification of TrlA

The cells were resuspended in buffer with 10 % glycerol (v/v), 300 mM NaCl, 50 mM sodium phosphate buffer (Na_2HPO_4/ NaH_2PO_4) at pH 7.4 (buffer A). After 15 min incubation on ice, the cells were lysed by ultrasonication (3 s pulse, 3 s pause; 4 min pulse time, amplitude: 60 %, 2 times). The lysate was centrifuged for 30 min at 18.000 x g to remove cell debris. The remaining supernatant was then filtered through a PVDF filter with pore size of 0.22 µm before being loaded on a Ni-NTA column (Cytiva) equilibrated with buffer A. Unspecific bound proteins were eluted by washing with 10 column volumes of buffer A and buffer B with a ratio of 96:4 (buffer B consists of buffer A + 500 mM imidazole). The His-gb1-tagged protein was eluted using 100 % buffer B. Small samples (20 µL) from each elution fraction were taken for SDS-PAGE analysis (see Figure S37), fractions containing the desired protein were pooled together and concentrated in an Amicon centrifugal filter (Sigma Aldrich). Subsequently, the buffer was exchanged using a desalting column (HiTrap Desalt, Cytiva), the obtained fractions concentrated and flash frozen in liquid nitrogen before being stored at -80°C.

1.5 Cloning, recombinant production and protein purification of TrlC

The procedure for cloning, recombinant production and purification of TrlC (NCBI accession number: AWF83807.1, see table S1), was carried out the same way as for TrlA. Only exception was using a vector with different tags for cloning, namely the pET-M11-His-TEV vector (for SDS-PAGE analysis see Figure S38).

1.6 Cloning, recombinant production and protein purification of TrlD

The procedure for cloning, recombinant production and purification of TrlD (NCBI accession number: AWF83808.1, see table S1), was carried out the same way as for TrlA (for SDS-PAGE analysis see Figure S39).

1.7 Cloning, recombinant production and protein purification of TrlE

The procedure for cloning, recombinant production and purification of TrlE (NCBI accession number: AWF83809.1, see table S1), was carried out the same way as for TrlA. Only differences were adding a spatula tip of FAD to the resuspended cells prior to sonication and supplementing buffer A and B during purification with 10 µM of FAD (for SDS-PAGE analysis see Figure S40).

1.8 Cloning, recombinant production and protein purification of TrlF

The procedure for cloning and recombinant production of TrlF (NCBI accession number: AWF83810.1, see Table S1), was carried out the same way as for TrlA. Only exception was using a vector with different tags for cloning, namely the pET-M11-MBP-TEV vector, containing a solubility enhancer protein MBP (Maltose binding protein) in front of the tobacco etch virus (TEV)-cleavage site at the N-terminus of the protein.

For protein purification the buffers and FPLC program were altered to suit the new MBP-tag. Buffer A contained 10 % glycerol (v/v), 200 mM KCl, 20 mM Tris, at pH 8 (buffer A) and buffer B contained 10 % glycerol (v/v), 200 mM KCl and 10 mM Maltose at pH 8. Elution was carried out using a linear gradient of B 0 % to 100 % buffer B within 10 column volumes) (for SDS-PAGE analysis see Figure S41).

1.9 Cloning, recombinant production and protein purification of TrlE Variants

Genes encoding variants of TrlE (amino acid exchange either, H213A, H213E, H213Q or Y217H) were obtained from TwistBioScience in a pET28a vector, which allowed for production of heterologous proteins with an N-terminal hexahistidine-tag followed by a gb1-tag and the tobacco etch virus (TEV)-cleavage site. The whole construct was flanked by NcoI and XhoI cleavage sites. Recombinant production and protein purification were carried out as described for TrlE (for SDS-PAGE analysis see Figures S42 - 45).

1.10 Recombinant production and purification of PaaABCE, PaaG, PaaZ-E256Q and PaaY

Recombinant production and purification of PaaABCE, PaaG, PaaZ-E256Q and PaaY were carried out as previously described.[2-4]

1.11 Multiple sequence alignment Trl gene cluster

A pBLAST was conducted with the sequence of TrlE from *Streptomyces cyaneofuscatus* Soc7 against the non-redundant protein sequences database from NCBI. The genomic environments of the putative *trlE* genes were scrutinized for other potential *trl* genes and newly identified gene clusters were then included in the multiple sequence alignment. Chosen sequences were aligned and visualized using the software CAGECAT[5].

1.12 Phylogenetic distance tree TrlE

Amino acid sequences of TrlE and known FAD-dependent group A monooxygenases with solved crystal structure were aligned using the ClustalW algorithm with default settings. The alignment was processed and visualized with the software Mega11[6] using the Jones-Taylor-Thornton (JTT) matrix based model and maximum likelihood method (1,000 replicates) (for PDB codes see Table S2).

1.13 Analytical size exclusion chromatography (SEC)

Analytical size exclusion chromatography was carried out using a Superdex 200 GL 10/300 column (Cytiva). The column was equilibrated with 10% glycerol, 300 mM NaCl, 50 mM sodium phosphate buffer (Na_2HPO_4/ NaH_2PO_4) at pH 7.4 for all his-tagged protein and 10 % glycerol, 20 mM Tris, 200 mM KCl at pH 8 for all MBP-tagged proteins. Proteins were run at 0.4 mL/min and molecular weights of the eluting main peaks were estimated from a previously generated calibration curve with proteins of known molecular weight (see Figure S46).

1.14 Determination of melting temperature (TM) of TrlE and variants

The melting temperatures of TrlE and its variants were determined using the Prometheus NanoDSF device from NanoTemper Technologies. Protein concentration was adjusted to 5 µM in 50 mM Tris-HCl buffer with pH 8. Possible binding partners were added to a final concentration of 1 mM. All measurements were performed in triplicates. Samples were equilibrated at 20°C prior to measurement start. During measurement the temperature increased for 1.5°C per minute until 95°C was reached.

1.15 Crystallization of TrlE, data collection and refinement

TrlE (8.8 mg/mL in 50 mM Tris pH 7.4, 10% glycerol and 250 µM FAD) was crystallized at room temperature using sitting-drop vapor diffusion method by mixing 300 nL of protein with 300 nL of precipitant (0.1 M Bis-Tris propane pH 8.5, 0.25 M Na_2SO_4, 15 %(w/v) PEG 3350). Small rods appeared after 13 days and grew in size for additional 20 days.

Data was collected at the SLS beamline X06SA (Swiss Light Source, Paul Scherrer Institute, Switzerland) at 100 K and were processed using XDS software[7] and scaled using aimless[8]. The crystal structure was determined by molecular replacement with Phaser[9] using an AlphaFold-model[10] of TrlE. Manual model building was done with Coot[11] and structure refinement in PHENIX[12] and REFMAC5[13]. Model quality was validated with Molprobity[14]. Data collection and refinement statistics are summarized in Table S3. The atomic coordinates and structure factors have been deposited in the Protein Data Bank under the accession code 8RQH.

1.16 Docking, molecular dynamics (MD) simulation and energy minimization

Dihydrotropone-2-carboxylate (**7**) was docked into the obtained crystal structure of TrlE using Induced Fit Docking protocol[13,14]. Compound **7** and the protein structure were prepared with standard settings using LigPrep (LigPrep, Schrödinger, LLC, New York, NY, 2024) and Protein Preparation Wizard[15], respectively. To define the search volume for docking, the homologous structure of salicylate hydroxylase with co-crystalized 2-hydroxybenzoic acid was first aligned to the crystal structure of TrlE.

The search volume was defined based on the position of 2-hydroxybenzoic acid in the superimposed structure.

Based on the top-ranked docking pose, molecular dynamics simulation was performed using Desmond[16] in an NPT-ensemble using OPLS2005 force field. Periodic boundary conditions were applied to the protein-ligand system in a cubic water box with a 10 Å water buffer around the protein. Electrostatic forces were treated using particle-mesh Ewald summation with a short range cut-off of 9 Å. After the default relaxation protocol, MD simulation was performed for 100 ns with frames stored every 100 ps. The MD trajectory was clustered and the representative structure of the largest cluster minimized using simulated annealing for 100 ps. The final structure was utilized for visual analysis and interpretation.

1.17 Chemical synthesis of phenylacetyl-CoA (PaCoA) (1)

Phenylacetyl-CoA was synthesized using phenylacetyl succinimide and coenzyme A as described in literature.[2, 15] Purification was carried out by means of preparative HPLC. The sample was filtered and applied to a Sunfire Prep C18 column (10x150 mm, Waters Corporation) equilibrated with 2 % acetonitrile and 98 % 10 mM ammonium acetate buffer (pH 4.5). The sample was eluted with a flow rate of 4 mL/min and absorption was monitored at 260 nm with an UV diode array detector. After collection, the sample was lyophilized and subsequently stored at -80°C upon further usage.

1.18 HPLC analysis

Assay analysis by HPLC was carried out on an Agilent 1100 chromatographic system, equipped with a SemiPrep VP NUCLEODUR Gravity SB column (250 x 10 mm ID, 5 µm, Macherey-Nagel) combined with a UNIVERSAL RP guard column (4 x 3 mm ID, Macherey-Nagel). Pre-equilibration of the column was conducted with either 10 mM AmAc pH 4.5 (solution A1) and acetonitrile (solution B1) in a ratio of 98:2 for analysis of aqueous samples or with H_2O + 0.1 % formic acid (solution A2) and acetonitrile + 0.1 % formic acid (solution B2) in a ratio of 98:2 for extracted compounds. The flow rate was set to 3 mL/min with following gradient: 2 % - 60 % B1/2 (0 - 15 min), 60 % B1/2 (15 - 16 min), 60 % - 2 % B1/2 (16 – 17 min), 2 % B1/2 (17 – 25 min). Absorption was monitored at 260, 300 and 340 nm by the DAD-detector. Purification of compounds from upscaled assays and cell cultures was performed on an Agilent 1100 chromatographic system equipped with an XBridge BEH C18 OBD Prep column (150 x 10 mm ID, 5 µm, Waters) equipped with a guard column. Pre-equilibration of the column was conducted using H_2O + 0.1 % formic acid (solution A2) and acetonitrile + 0.1 % formic acid (solution B2) in a ratio of 95:5. The flow rate was set to 4 mL/min with following gradient: 5 % B2 (0 – 2 min), 5 % – 12 % B2 (2 – 7 min), 12 % B2 (7 – 9 min), 12 % – 60 % B2 (9 – 17 min), 60 % B2 (17 – 23 min), 60 % – 100 % B2 (23 – 24 min), 100 % B2 (24 – 27 min), 100 % – 5 % B2 (27 – 28 min), 5 % B2 (28 – 33 min). Absorption was monitored at 260, 280, 300, 350 and 370 nm as all of these wavelengths were maxima of different compounds occurring in the enzymatic assays.

1.19 LC-HRMS and LC-MS analysis

LC-HRMS and LC-MS analysis of assays was carried out on two different systems. The system used for LC-HRMS was a Waters Acquity UPLC H class system coupled with a diode array detector. For LC-MS analysis a Shimadzu LCMS-8030 Triple Quad Mass Spectrometer was used, equipped with an analytical SunFire C18 column (150 x 3 mm ID, 3.5 µm, Waters) combined with a guard column (10 x 3 mm ID). Pre-equilibration of the column was conducted with either 10 mM AmAc pH 4.5 (solution A1) and acetonitrile (solution B1) in a ratio of 98:2 for analysis of aqueous samples or with H_2O + 0.1 % formic acid (solution A2) and acetonitrile + 0.1 % formic acid (solution B2) in a ratio of 98:2 for extracted compounds. The flow rate was set to 0.4 mL/min with following gradient: 2 % - 12 % B1/2 (0 – 4 min), 12 % B2 (4 – 6 min), 12 % - 60 % B1/2 (6 – 14 min), 60 % B1/2 (14 - 18 min), 60 % - 100 % B1/2 (18 – 19 min), 100 % B1/2 (19 – 24 min), 100 % - 2 % B1/2 (24 – 25 min), 2 % B1/2 (25 – 30 min). Absorption

was monitored from 190 – 800 nm. Samples were analyzed in MS ESI positive and negative mode with a capillary voltage of 3 kV, 250°C DL temperature, 400 °C heat block temperature and 3 L/min nebulizing gas flow.

1.20 Enzymatic synthesis of 2

In order to produce **2**, an assay containing 0.5 mM PaCoA (**1**), 1 mg/mL PaaABCE and 1 µM PaaG was prepared in 50 mM Tris-HCl pH 8 buffer and incubated at 30°C for 2 minutes. Afterwards, 1.5 mM of NADPH was added to start the reaction. The assay was incubated at 30°C and 900 rpm for 10 min and stopped with methanol. The sample was lyophilized overnight and subsequently resuspended in water. Purification was carried out by means of preparative HPLC. The sample was filtered and applied to a SemiPrep VP NUCLEODUR Gravity SB column (250 x 10 mm ID, 5 µm, Macherey-Nagel) combined with a UNIVERSAL RP guard column (4 x 3 mm ID, Macherey-Nagel) and equilibrated with 2 % acetonitrile and 98 % 10 mM ammonium acetate buffer (pH 4.5). The sample was eluted with a flow rate of 3 mL/min and absorption was monitored at 260 nm with an UV diode array detector. After collection, the sample was lyophilized and subsequently stored at -80°C upon further usage.

1.21 Enzymatic synthesis of 3

In order to produce **3**, an assay containing 0.5 mM PaCoA (**1**), 1 mg/mL PaaABCE, 1 µM PaaG and 1.5 µM PaaZ-E256Q was prepared in 50 mM Tris-HCl pH 8 buffer and incubated at 30°C for 2 minutes. Afterwards, 1.5 mM of NADPH (1) was added to start the reaction. The assay was incubated at 30°C and 900 rpm for 10 min. The resulting substrate mix was then used in subsequent assays.

1.22 Turnover assay with TrlF

To investigate the enzymatic function of TrlF, 350 µL of the substrate mix (described in production of **3**) was prepared freshly. 50 µL were set aside and quenched with an equal amount of ethyl acetate + 1 % formic acid serving as the control sample. The other 300 µL were incubated with 2.5 µM TrlF at 30°C and 900 rpm. Samples with 50 µL each were taken after 0, 2, 4, 6, 8 and 10 min and quenched with an equal amount of ethyl acetate + 1 % formic acid. Following this, all samples were centrifuged for 10 min at 18.000 x g. The ethyl acetate fractions were dried in a speed-vac for 1 h and resuspended in 50 µL of acetonitrile before being analyzed by HPLC-DAD and LC-MS.

1.23 Carbonic Anhydrase Test

Carbonic anhydrase activity of TrlF was tested in assays measured on a plate reader MultiSkan Go (ThermoScientific) using the pH indicator dye phenol red. In 60 µL of 20 mM Tris-HCl pH 8 buffer and 40 µL of CO_2-saturated water phenol red is added to a final concentration of 5 µM. The CO_2-saturated water was prepared by putting dry ice into distilled and deionized water and letting it dissolve for 30 min. The reaction was started by adding different amounts of TrlF to the solution. Controls were analyzed without the addition of any protein. After reaction start the OD557nm was measured every second. All measurements were performed in triplicates.

1.24 Turnover assay with TrlE

To investigate the enzymatic function of TrlE, 400 µL of the turnover assay with TrlF were prepared freshly and incubated for 10 min at 30°C and 900 rpm. Then, TrlE and NADPH were added to a final concentration of 0.1 µM and 1.5 mM respectively. The assay was incubated at 30°C and 900 rpm. Samples with 50 µL each were taken after 0, 0.5, 1, 1.5, 2, 2.5, 3, 3.5 and 4 min and quenched with an equal amount of ethyl acetate + 1 % formic acid. Subsequently, all samples were centrifuged for 10 min at 18.000 x g. The ethyl acetate fractions were dried in a speed-vac for 1 h and resuspended in 50 µL of acetonitrile before being analyzed by HPLC-DAD and LC-MS.

1.25 Tropone turnover assay with TrlE

To rule out the possibility of tropone being the native substrate for TrlE, an assay containing 0.5 mM of tropone (commercially available standard, Alfa Aesar) and 2.5 µM TrlE and 1.5 mM NADPH in 100 µL Tris-HCl pH 8 was conducted. The reaction mixture was incubated at 30°C and 900 rpm for 10 min and subsequently quenched with an equal amount of ethyl acetate + 1 % formic acid. The sample was dried in a speed-vac for 1 h and resuspended in 100 µL of acetonitrile before being analyzed by HPLC-DAD.

1.26 Turnover assay with TrlCD

In order to assess the function of the two component flavoprotein monooxygenase TrlCD, an assay containing tropolone and a NADH regeneration system was conducted. The reaction mixture contained 25 µM TrlC, 125 µM TrlD, 100 µM sodium formate, 4 U/mL formate dehydrogenase, 0.1 mg/mL catalase, 30 µM FAD, 5 mM NADH and 0.5 mM tropolone in Tris-HCl pH 8. The assay was incubated at 30°C and 900 rpm. 50 µL samples were taken at 0, 0.5, 1, 2, 3, and 4 hours and quenched with an equal volume of ethyl acetate + 1 % formic acid. The samples were centrifuged at 18.000 x g and the ethyl acetate fractions were subsequently dried under N2 gas flow. Samples were resuspended in a 4:1 mixture of water and acetonitrile containing 50 mM of EDTA before being analyzed by HPLC-DAD and LC-MS. The assay was also conducted with a substrate mix containing enzymatically produced tropolone (as described in the turnover assay with TrlE) as a control. Since there were no observable changes in the outcome of the assay, commercially available tropolone was used hereafter to reduce assay complexity.

1.27 Comparison of TrlA and PaaZ-E256Q

To investigate the possible hydratase function of TrlA in comparison to PaaZ-E256Q, assays were conducted containing 0.5 mM PaCoA (**1**), 1 mg/mL PaaABCE, 1 µM PaaG and 1.5 µM of either PaaZ-E256Q or TrlA, respectively, which were incubated in 50 mM Tris-HCl pH 8 buffer at 30°C for 2 minutes. Afterwards, 1.5 mM of NADPH was added to start the reaction. After 15 min the reaction was stopped with an equal volume of methanol. The samples were vortexed, centrifuged at 18.000 x g and subsequently dried for 30 min in a speed-vac before being analyzed by HPLC.

1.28 Oxepin-CoA assay TrlE

To assess the necessity of NADPH for tropolone formation by TrlE, an assay using oxepin-CoA as starting substrate was prepared. 1 mM of oxepin-CoA was incubated with 1.5 µM of PaaZ-E256Q at 30°C and 900 rpm for 10 min. Subsequently, 2.5 µM TrlF was added and the assay was incubated for another 10 min with the same conditions. Afterwards, the reaction mixture was split into two equal parts. 2.5 µM TrlE was added to one of the assays. Both reaction mixtures, with and without TrlE, were incubated for an additional 10 min at 30°C and 900 rpm. The assays were stopped with an equal volume of ethylacetate + 1 % formic acid, vortexed and centrifuged at 18.000 x g. Samples were dried in a speed-vac for 30 min, volumes were adjusted to 50 µL with acetonitrile, before being analyzed by HPLC.

1.29 $^{18}O_2$-labeling assays

To study oxygen incorporation of O_2 into tropolone, assays were conducted with ^{18}O labeled oxygen. **3** was produced by mixing 250 µM of oxepin-CoA with 5 µM of PaaZ-E256Q in an anoxic environment (glove box) in Tris-HCl pH 8 and the mixture then incubated for 10 min at RT. Afterwards, TrlF was added to a final concentration of 2.5 µM and incubated for another 10 min at RT. TrlE was added to a final concentration of 2.5 µM to the assay, then the reaction mixture was split into two equal parts. Both reaction mixtures were transferred to an anaerobic air-tight bottle with septum. Upon removal from the glove box ~2 ml of $^{18}O_2$ was injected into one of the bottles, the other was opened. Both reaction mixtures were incubated at 30°C and 900 rpm for 15 min, then ethylacetate + 1 % formic acid was added to stop the reaction and extract the reaction products. Samples were vortexed and

centrifuged at 18.000 x g afterwards. Subsequently, the samples were dried in the speed-vac for ~30 min, the volume was adjusted to 50 µL using acetonitrile, before analyzing ^{18}O incorporation on the LC-HRMS system.

1.30 Differential scanning fluorimetry (DSF) measurements

In order to determine the melting point of all produced proteins DSF measurements were carried out on a NanoDSF Prometheus from NanoTemper Technologies (Munich, Germany). Protein concentrations were adjusted to 10 µM in degassed 50 mM Tris-HCl buffer with pH 8. Ligands were dissolved in the same buffer and as concentration 50 µM for FAD and 1 mM for salicylate was used as final concentrations. The temperature increase was set to 1.5°C per minute and the overall temperature gradient was set from 25°C to 90°C. All measurements were performed in triplicates.

1.31 Re-oxidation of 4

To investigate the proposed ring oxidation step by TrlE in the course of **4** synthesis, a re-oxidation assay was conducted in which 25 µM of **4** in Tris-HCl pH 8 was first reduced using 200 µM of sodium dithionite. This chemical reduction in aqueous solution could be followed by monitoring the absorption at 340 nm using a NanoPhotometer NP80 device (Implen), which subsequently also allowed to determine the rate of re-oxidation to **4** for different concentrations of TrlE (0.5, 1 and 2 µM). As controls, heat-denatured TrlE with the same concentrations (0.5, 1 and 2 µM) as well as pure FAD (2 µM) were used. All measurements were performed with n = 4.

1.32 Cultivation of *Longimycelium tulufanense*

L. tulufanense was cultivated on GYM Streptomyces medium agar plates (D-glucose 4 g/L, yeast extract 4 g/L, malt extract 10 g/L, $CaCO_3$ 2 g/L and agar 12 g/L). A single colony was picked from the agar plate after 5 days of cultivation and incubated in 50 mL GYM-NaCl medium (D-glucose 4 g/L, yeast extract 4 g/L, malt extract 10 g/L, NaCl 4 g/L and $CaCO_3$ 2 g/L) at 140 rpm and 37°C for 5 days. The culture was harvested by centrifugation at 18.000 x g for 30 min, the supernatant as well as the pellet fraction were extracted with EtOAc + 1 % FA. The extracts were dried under N_2 gas flow and resuspended in a 4:1 mixture of water and acetonitrile containing 50 mM of EDTA before being analyzed by HPLC-DAD and LC-MS.

1.33 NMR measurement of 5

Compound **5** was obtained from an upscaled enzymatic reaction (20 mL) described under 1.25. The reaction mixture was extracted two times with an equal amount of EtOAc + 1 % FA. The combined organic phases were dried using N_2 and the dried residue was purified using semi-preparative HPLC collecting the peak containing compound **5** (see 1.18). Fractions containing isolated **5** were extracted two times with an equal amount of EtOAc + 1 % FA, the organic phases combined and dried under N_2 gas flow and subsequently stored at -80°C until NMR measurement.

NMR samples were dissolved in 120 µL of deuterated benzene (C_6D_6) and transferred into 3 mm NMR tubes. Spectra were measured on a Bruker Avance III NMR spectrometer operating at 500.13 MHz for ^{1}H and 125.77 MHz for ^{13}C nuclei. ^{1}H, ^{13}C, COSY, HSQC, and HMBC spectra were recorded at 23°C on a BBO probe.

Compound **5**: ^{1}H NMR (500 MHz, C_6D_6) δ ppm 6.24 (m, H-4/5) δ ppm 6.97 (m, H-3/6), ^{13}C NMR (126 MHz, C_6D_6) δ ppm 120.4 (C-3/6) 129.3 (C-4/5) 160.1 (C-2/7) 169.8 (C-1).

2. Supplemental Figures and Tables:

Table S1 Trl gene cluster of *Streptomyces cyaneofuscatus* Soc7

Trl gene cluster		Annotation	AA
**TrlA**	AWF83805.1	Enoyl hydratase	159
**TrlB**	AWF83806.1	DAHP synthase	484
**TrlC**	AWF83807.1	Tropolone 3,7-monooxygenase oxidase component	530
**TrlD**	AWF83808.1	Tropolone 3,7-monooxygenase flavin reductase component	185
**TrlE**	AWF83809.1	Tropone 2-monooxygenase	400
**TrlF**	AWF83810.1	1,4,6-cycloheptatriene-1-carboxylic acid decarboxylase	203
**TrlG**	AWF83811.1	putative A-factor biosynthesis hotdog domain-containing protein	253
**TrlH**	AWF83812.1	Bifunctional prephenate dehydratase and chorismate mutase	402
**TrlI**	AWF83813.1	TetR family transcriptional regulator	209

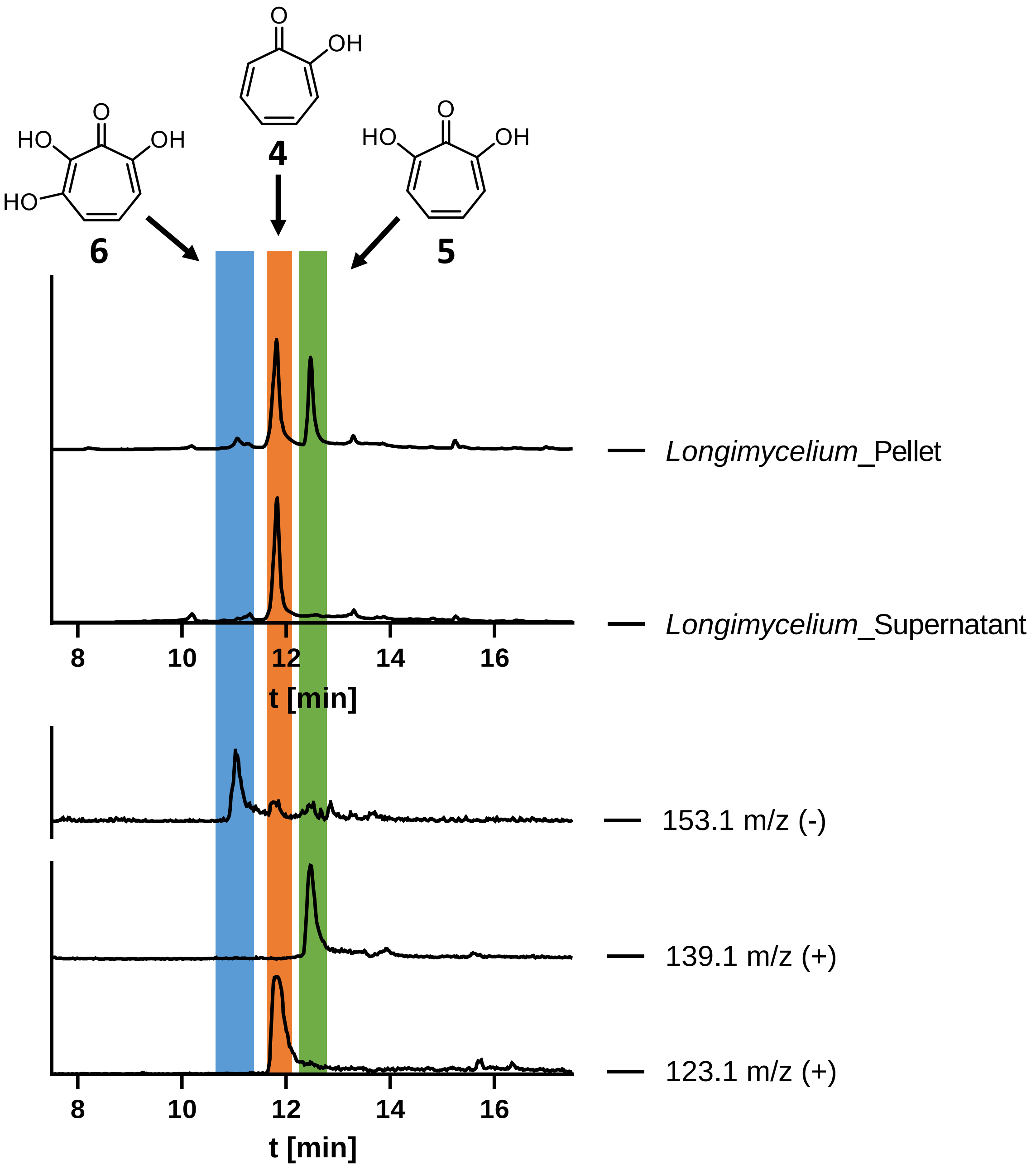

Figure S1 Observed production of 4, 5, and 6 in *Longimycelium tulufanense.* After 5 days of incubation of *Longimycelium tulufanense* in GYM-NaCl media at 37°C and 140 rpm, the three distinct (hydroxyl)tropolones 4, 5 and 6 could be observed in the supernatant as well as in the pellet fraction of harvested cultures. Shown are the UV traces from RP-HPLC (300 nm) and the extracted ion chromatograms (EIC) with corresponding masses in the ionization modes which gave the best signals (positive mode for 4 and 5, negative mode for 6, all ESI). Retention time, UV-visible spectra and masses measured on the LCMS system matched the data obtained in the *in vitro* assay with the *Trl* enzymes. Samples were measured with the HPLC program for the analytical SunFire column (see above in method section, 1.19 LC-MS analysis).

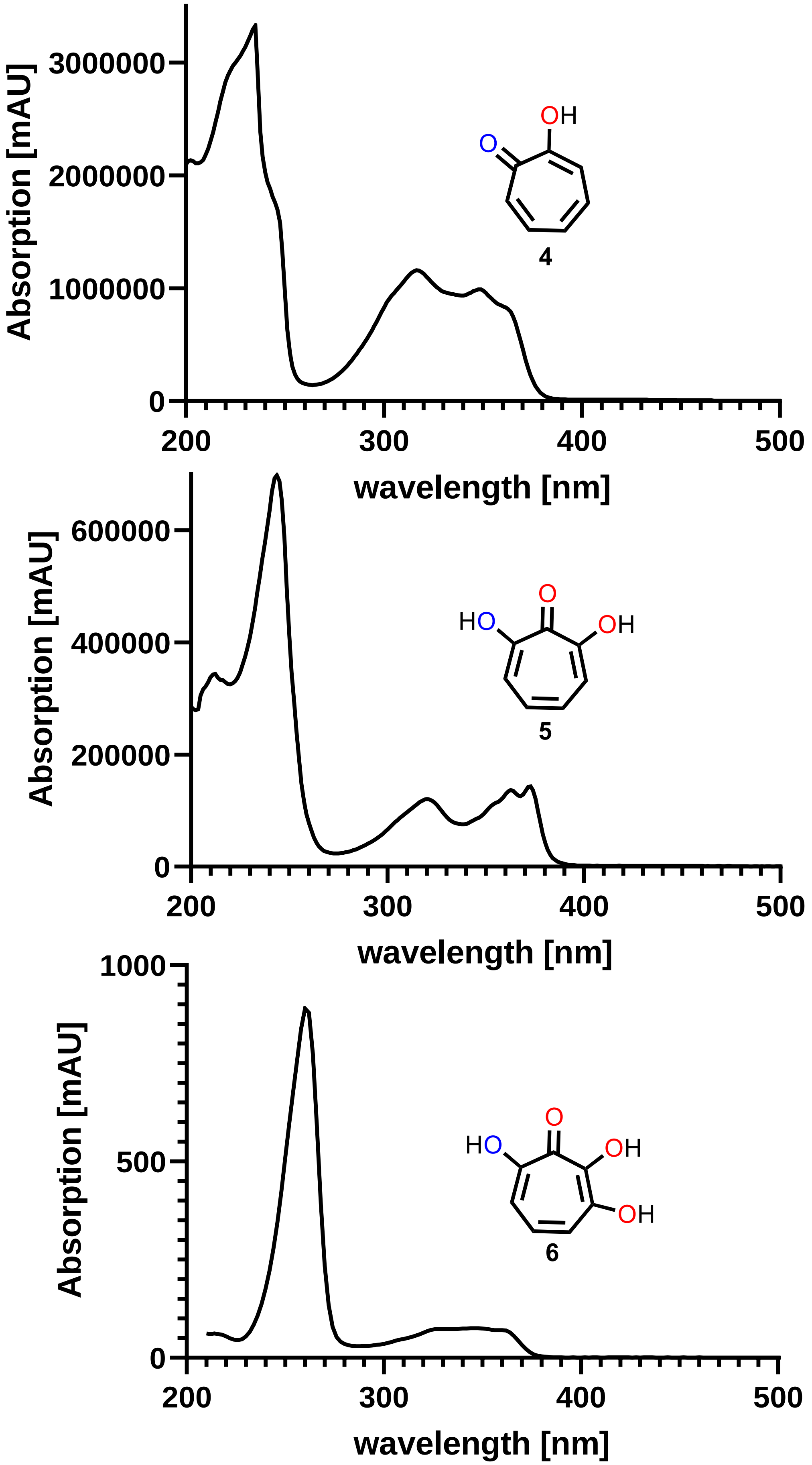

Figure S2 UV-visible spectra of compounds 4, 5 and 6

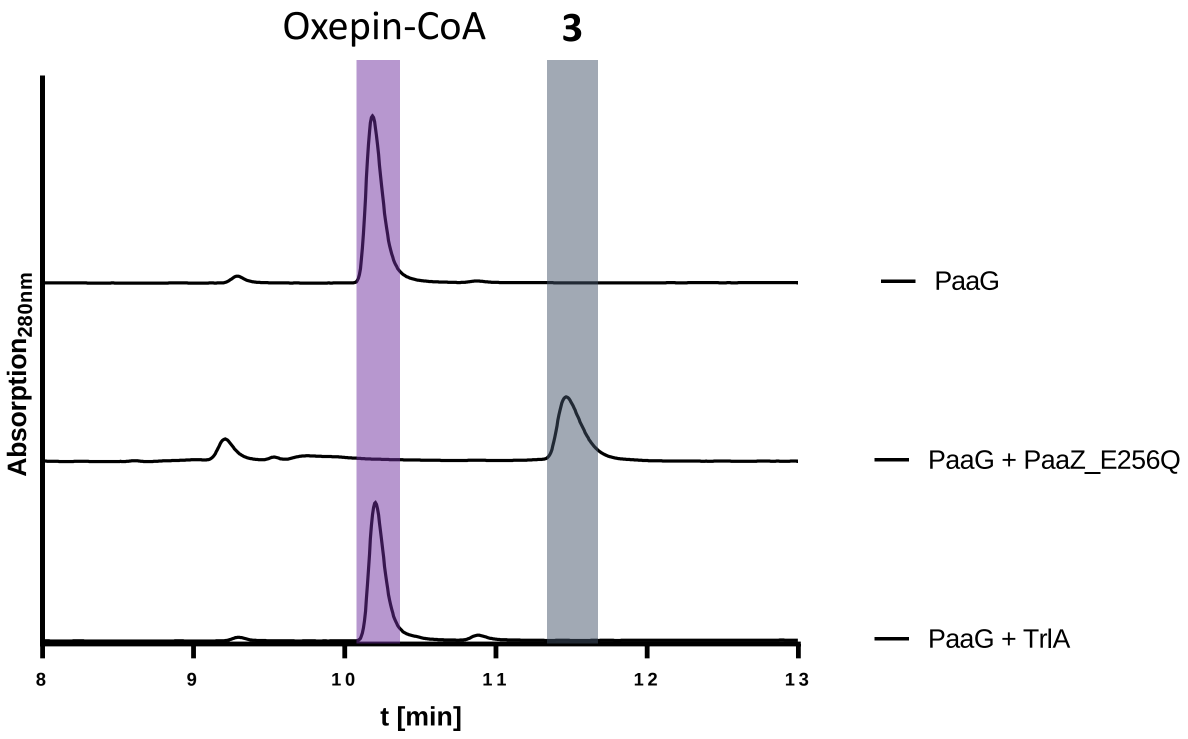

Figure S3 Functional test of putative hydratase TrlA. No visible conversion of the substrate oxepin-CoA (produced by PaaG) was observed in the assay when TrlA was added. When the enoyl-CoA hydratase variant PaaZ-E256Q was added full conversion of oxepin-CoA to 3 was observable. Samples were measured with the HPLC program for the semiprep nucleodur column (see above in method section, 1.18 HPLC analysis).

```
TrlA_Streptomyces_cupreus               --------MGATRLTTDDIITYAR-SFDPMPFHLDPEAAR-RSPFGGLVASGWHTGAVIM   50
TrlA_Streptomyces_cyaneofuscatus_Soc7   FRVGSTYELGTTRLTSEDIIGYAR-VWDPMPFHLDPEAAA-RSPFGGLVASGWHTGAVVM   64
TrlA_Streptomyces_sp._NRRL_B-1140       FRVGGTYELGATRLTSEDIMGYAR-VWDPMPFHLDPEAAA-RSPFGGLVASGWHTGAVVM   64
TrlA_Longimycelium_tulufanense          FHAGDRHELGRVVVTRDEIVEYAR-RWDPMPFHTDEAAAA-AGPFGGLVASGGHTTAHVT   64
TrlA_Amycolatopsis_regifaucium          FVTGDEHQLGQVSMTEEEIIGYAR-QWDPMPFHTDEAAAS-AGPFGKLVASGNHTTAHAT   64
Paaz_Thermus_thermophilus               LEVGETLTTHRRTVTEADIALFSALSWDHFYAHTDEIAAR-ESLFGKRVAHGYFVLSAAA   584
PaaZ_Phaeobacter_italicus               LAVGETLHTAPRTVTLEDIETFAHFTGDTFYAHMDDEAAKRNPFFPGRVAHGYLLLSFAA   600
PaaZ_Escherichia_coli_K12               LQPGDSLLTPRRTMTEADIVNFACLSGDHFYAHMDKIAAA-ESIFGERVVHGYFVLSAAA   592
PaaZ_Klebsiella_pneumoniae              IQPGDSLLTPRRTLTEADIVNFACLSGDHFYAHMDKIAAA-ESIFGERVVHGYFLISAAA   592
                                                     :*  :*  ::    * :  * *  **     *   *. *    :
```

Figure S4 Multiple sequence alignment of TrlA from *Streptomyces cyaneofuscatus* Soc7, PaaZ from *E. coli* K12 and homologue proteins from other organisms. Catalytically important residues are highlighted in red. The alignment was generated with ClustalOmega using the default parameter settings.

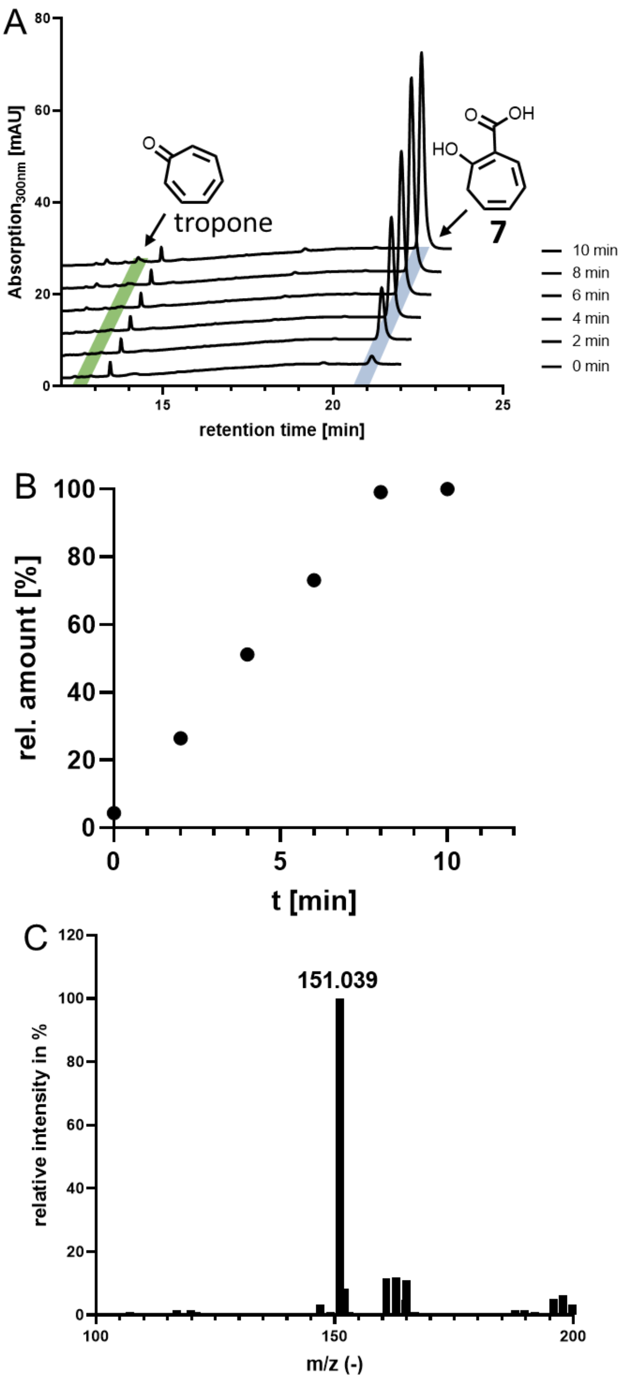

Figure S5 Time course of 7 formation by TrlF (A) RP-HPLC chromatograms at 300 nm showing the time course of the formation of **7** (from **3**, which is not shown here, as the polar CoA esters were not extracted by organic solvents), highlighted in blue. Small amounts of tropone (**8**) could be observed after prolonged incubation time (highlighted in green). **(B)** Relative amounts of **7** (as determined by the area under each peak) produced by TrlF in A. **(C)** Mass spectrum of 7 in negative ion mode. Samples were measured with the HPLC program for the semiprep nucleodur column (see above in method section, 1.18 HPLC analysis).

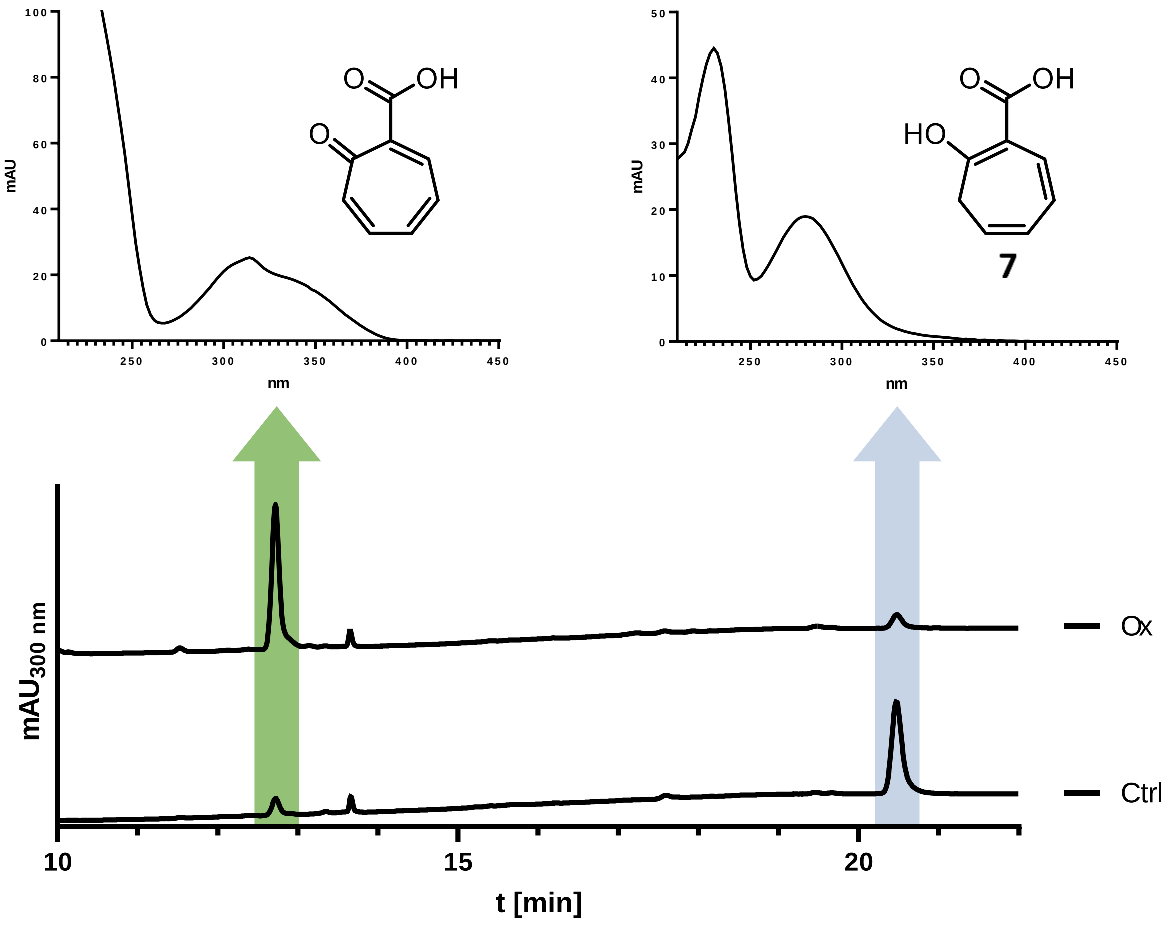

Figure S6 Oxidation of 7 to tropone-2-carboxylate (verified by chemically synthesized standard). 1 mM of potassium ferricyanide (III) was incubated with **7** for 5 min at 30°C and analyzed subsequently by RP-HPLC. Oxidation product was compared to a chemically synthesized standard and exhibited the same retention time and UV-visible spectrum. Samples were measured with the HPLC program for the semiprep nucleodur column (see above in method section, 1.18 HPLC analysis).

```
CA_Acinetobacter_baumannii             ------------------------------------MPCYSIDGVIPVVSPDAFVHPTAV   24
CaiE_Alphaproteobacteria_bacterium     -----------------------------------MAQVYSIDGVIPVVDPTAFVHPSAI   25
PaaY_Acidimicrobiaceae_bacterium       ------------------------------------MGIYEIEGVVPVVHPTAFVHPEAV   24
CA_Yinghuangia_soli                    -----------------------------------MARVYEIDSVVPVIDPTAFVHPDAV   25
TrlF_Amycolatopsis_regifaucium         -----------------------------------MARVYEIDGVVPVIAPEAFVHPDAV   25
TrlF_Longimycelium_tulufanense         --------------MTPSWKYSLPP-TESHELEEPVARVYEIDGVVPLIHPNAFVHPDAV   45
PaaY_Streptomyces_sp                   -----------------------------------MARIYAFEGHVPVVHPTAFVHPDAV   25
TrlF_Streptomyces_Cyaneofuscatus_Soc7  -----------------------------------MARTYSFEGNVPVVHPTAFVHPDAV   25
TrlF_Streptomyces_sp_WAC04114          -----------------------------------MARIYSFEGNVPVVHPTAFVHPDAV   25
CA_Thermus_thermophilus                ----------------------------------GSMSVYRFEDKTPAVHPTAFIAPGAY   26
CA_Burkholderia_Pseudomallei           MRGSHHHHHHGMASMTGGQQMGRDLYDDDDKDHPFTMTIYKLGENAPSIHESVFVADSAT   60
                                                                              * :    * :   .*:   *

CA_Acinetobacter_baumannii             LIGDVIIEAGVYVGPFASLRADFGRIHINQNANIQDSCTVHGFPQSVTLVEEMGHIGHGA   84
CaiE_Alphaproteobacteria bacterium     LIGDVIVGPGCYVGPAASLRGDFGRLILERGANLQDTCVMHGFPGTDTVVEEDGHVGHGA   85
PaaY_Acidimicrobiaceae_bacterium       LIGDVLIGEGCYVGPLASLRGDFGQVVVRAGANVQDGCVLHCFPGRDVVVDEDGHIGHGA   84
CA_Yinghuangia_soli                    LVGDVVIGPRCYIGPLVSLRGDFGRITVGPGANVQDGCVVHCFPGTDTVIEEDGHVGHGA   85
TrlF_Amycolatopsis_regifaucium         LIGDVLIGPGCYIGPLASLRGDFGRIEVRAGANIQDGCVVHSFPGSVTVVGENGHVGHGT   85
TrlF_Longimycelium_tulufanense         LIGDVVIGAGCYVGPLASLRGDFGRIVLEEGANIQDGCVAHCFPGSSTVVEQDGHVGHGA   105
PaaY_Streptomyces_sp                   LIGSVDIGPGCYVGPLASLRGDFGHIELRAGSNVQDGCVLHCFPGADTVVEEDGHVGHGS   85
TrlF_Streptomyces_Cyaneofuscatus_Soc7  LIGSVDIGPGCYVGPLASLRGDFGHIELRAGSNVQDGCVLHCFPGADTVVEEDGHVGHGS   85
TrlF_Streptomyces_sp_WAC04114          LIGSVDIGPGCYVGPLASLRGDFGHIALRAGSNVQDGCVLHCFPGADTVVEEDGHVGHGS   85
CA_Thermus_thermophilus                VVGAVEVGEGASIWFGAVVRGDLERVVVGPGTNVQDGAVLHADPGFPCLLGPEVTVGHRA   86
CA_Burkholderia_Pseudomallei           IVGKVVLEENASVWFGATIRGDNEPITVGAGSNVQEGAVLHTDPGCPLTIAPNVTVGHQA   120
                                       ::* * :     :   . :*.*   : :  .:*:*: .. *  *     :     :** :

CA_Acinetobacter_baumannii             ILHGCRIGKNVLVGMNSVILDYAEIGENTIIGANSLVKTKDIIPANVLAMGSPAKVARDL   144
CaiE_Alphaproteobacteria bacterium     VLHGCIVKRDALIGMNAVIMDGAVIGESAIVAAMAFVKAGFEVPPRMLVAGIPAKILRPV   145
PaaY_Acidimicrobiaceae_bacterium       VLHGCHIGRGVLVGMNSVVMDGAAIGDFTFVGACTFVRAEMEVPARHVVAGNPARVLREL   144
CA_Yinghuangia_soli                    ILHGCRVGRGVLVGMNAVVMDGVDLGEYAFVAAHTFVKAGTAVPARHLITGSPGVVTREL   145
TrlF_Amycolatopsis_regifaucium         VLHGCQVGRDVLVGMNSVLMDGVIVEDESFVGAMSFLKAETRVPARSLIAGSPAKVLREL   145
TrlF_Longimycelium_tulufanense         VLHGCRVGRGALIGMNSVLMDHVVVGERAFVGANSFVKSGFEVPAAHLATGSPAKVLREL   165
PaaY_Streptomyces_sp                   VLHGCRVGRDSLIGMKSVLMDGVVVGRQAFVGAGSFVKSRFQVPDRHLVAGSPAKVVREL   145
TrlF_Streptomyces_Cyaneofuscatus_Soc7  VLHGCRVGRDSLIGMKSVLMDGVVVGTRAFVGAGSFVKSRFQVPERHLVAGSPAKVVREL   145
TrlF_Streptomyces_sp_WAC04114          VLHGCRVGRDSLIGMKSVLMDGVVVGEQAFVGAGSFVKSRFEVPERHLVAGSPAKVVREL   145
CA_Thermus_thermophilus                VVHGAVVEEGALVGMGAVVLNGARIGKNAVVGAGAVVPPGMEVPEGRLALGVPARVVRPI   146
CA_Burkholderia_Pseudomallei           MLHGCTIGEGSLIGIQAVILNRAVIGRNCLVGAGAVITEGKAFPDNSLILGAPAKVVRTL   180
                                       ::**. : .. *:*: :*::: . :    .:.* :.:     .*   :  * *. : * :

CA_Acinetobacter_baumannii             SEQEKKWKTRGTQEYMELAQRCLNSMQEVQPLSSESDDRLTYKDFSS--------SNYQI   196
CaiE_Alphaproteobacteria bacterium     TDEEIAWKREGTEDYQRLAIRSMKTMRPVEPLTEVEPDRPRTDSGA---SVPLYLKK--G   200
CA_Yinghuangia_soli                    TDTELAWKANGTRVYQDLAARSLASLRPATALTAVEPDRRRVGTD---TSVAVPLHTYRE   202
TrlF_Amycolatopsis_regifaucium         TDVEIDWKANGTRTYQDLARRSLASLRETKPAAWEEPGRRGFAGSDGTEPVHVTLHSYRG   205
TrlF_Longimycelium_tulufanense         TEDEMAWKANGTRVYQDLARRCRKTLKPAEPLVGDPTEHRRAVTTAGGEPVHVTLPEYRN   225
PaaY_Streptomyces_sp                   TADEIAWKGNGTLQYQKLAQRCLTGLHPVEPATERTPG---VAPAHSGEAEHVTLHQYRA   202
TrlF_Streptomyces_Cyaneofuscatus_Soc7  TADEIAWKGNGTAQYQKLAQRCLTGLHAAEAATERTAP---AAPAEPGEHEHVTLHAYRS   202
TrlF_Streptomyces_sp_WAC04114          TADEIAWKGNGTAQYQKLAQRCLTGLHPAEAATERAAA---PAPTRPGEPEHVTLHAYRS   202
CA_Thermus_thermophilus                DPPGNAPR------YRALAERYRKALFPVAT-----------------------------   171
CA_Burkholderia_Pseudomallei           SDEDIARMHMNTKSYAMRRAYFKEQLVRIG------------------------------   210
                                                     *          :

CA_Acinetobacter_baumannii             KQDSV----------------     201
CaiE_Alphaproteobacteria bacterium     ETQ------------------     203
PaaY_Acidimicrobiaceae_bacterium       S--------------------     202
CA_Yinghuangia_soli                    RDREHDREQNTGGADANGAPL     223
TrlF_Amycolatopsis_regifaucium         R--------------------     206
TrlF_Longimycelium_tulufanense         Q--------------------     226
PaaY_Streptomyces_sp                   R--------------------     203
TrlF_Streptomyces_Cyaneofuscatus_Soc7  R--------------------     203
TrlF_Streptomyces_sp_WAC04114          R--------------------     203
CA_Thermus_thermophilus                ---------------------     171
CA_Burkholderia_Pseudomallei           ---------------------     210
```

Figure S7 Multiple sequence alignment of TrlF from *Streptomyces cyaneofuscatus* Soc7 and homologues from other organisms. PaaY represents the phenylacetic acid degradation protein PaaY, CA represents carbonic anhydrase functionality. Residues expected to be important for the trimeric interface are highlighted in red, residues expected to be important for metal binding (most likely Zn^{2+}) are highlighted in light blue. The alignment was generated with ClustalOmega, using the default parameter settings.

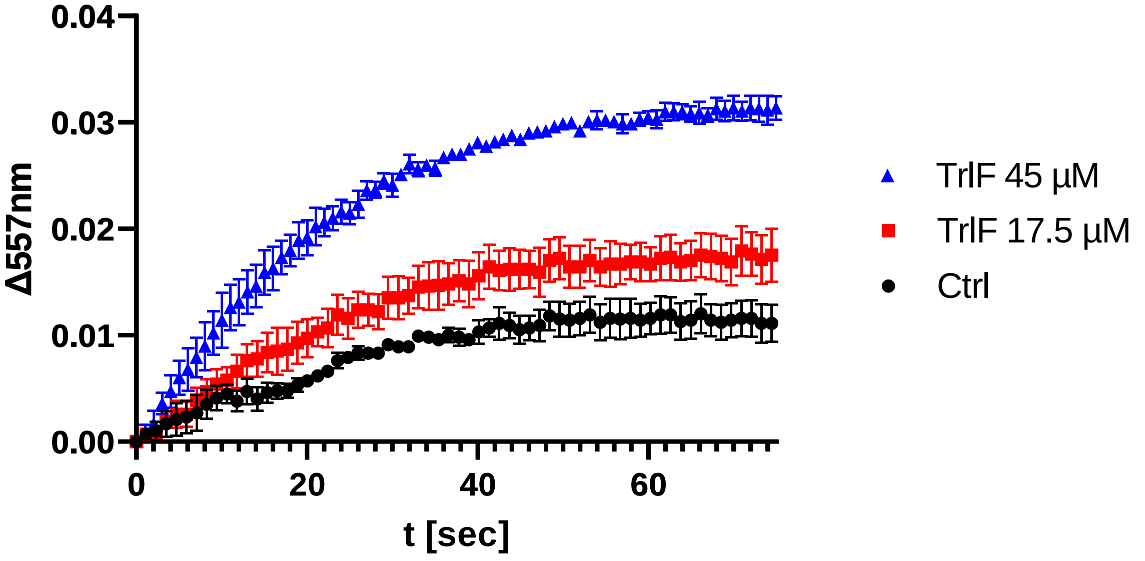

Figure S8 Test for carbonic anhydrase activity of TrlF. Two different concentrations of TrlF (blue: 45 µM, red: 17.5 µM) were incubated with the pH indicator dye phenol red in 20 mM Tris-HCl pH 8 buffer and CO_2-saturated water. The control group was incubated without any enzyme (black). Changes of the OD_{557nm} were monitored over time. All samples were prepared in triplicates.

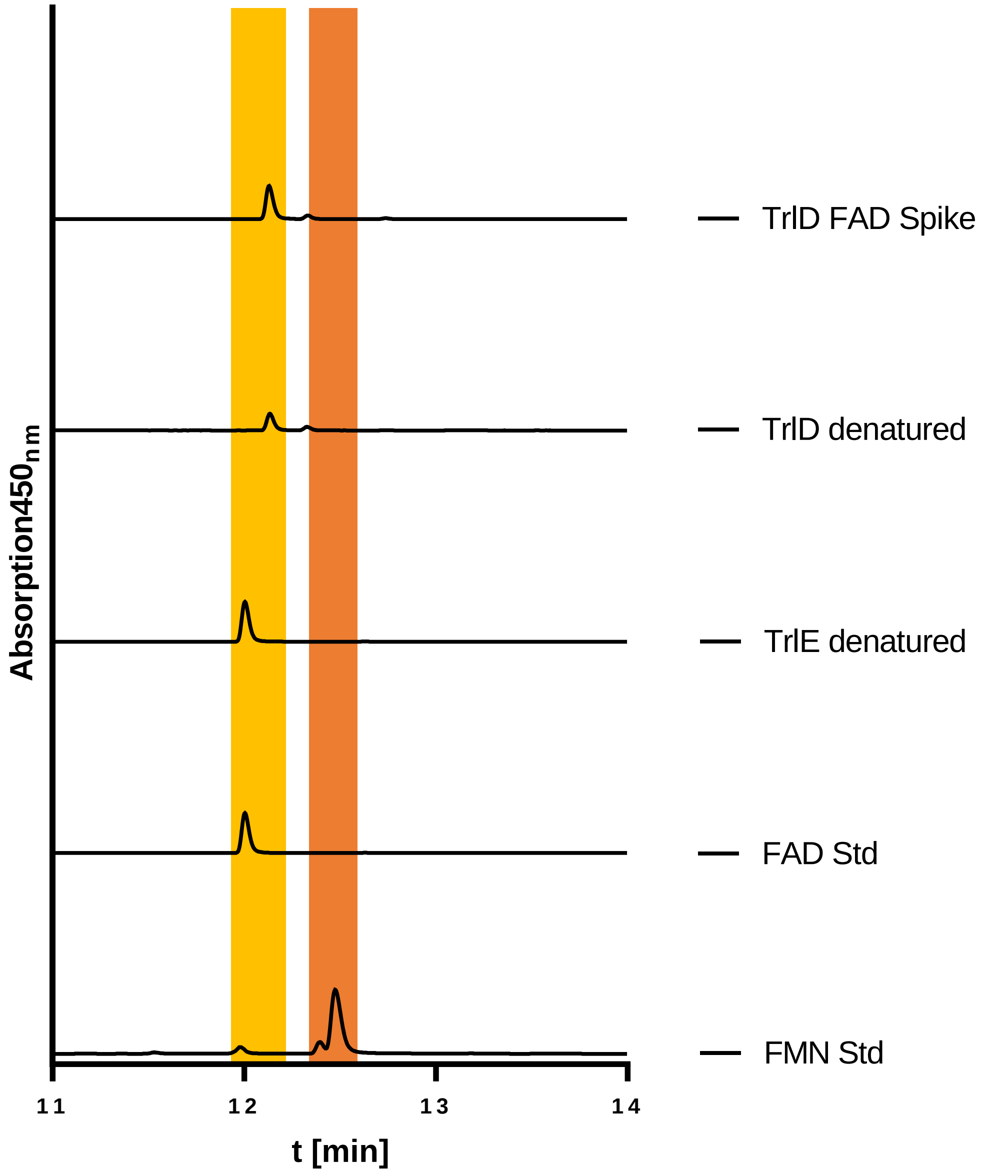

Figure S9 RP-HPLC analysis (450 nm) of isolated cofactors from TrlE and TrlD. Enzymes were heated at 95°C for 10 min, centrifuged for 10 min at 13.000 x g and the supernatant applied to the HPLC. Retention time of the cofactor was compared to commercially available standards. The slight shift in retention time for TrlD was due to some protein aggregates still being in the supernatant (peak observable at 280 nm). Spiking with FAD increased the cofactor peak, indicating FAD as the correct cofactor. Samples were measured with the HPLC program for the analytical SunFire column (see above in method section, 1.19 LC-MS analysis).

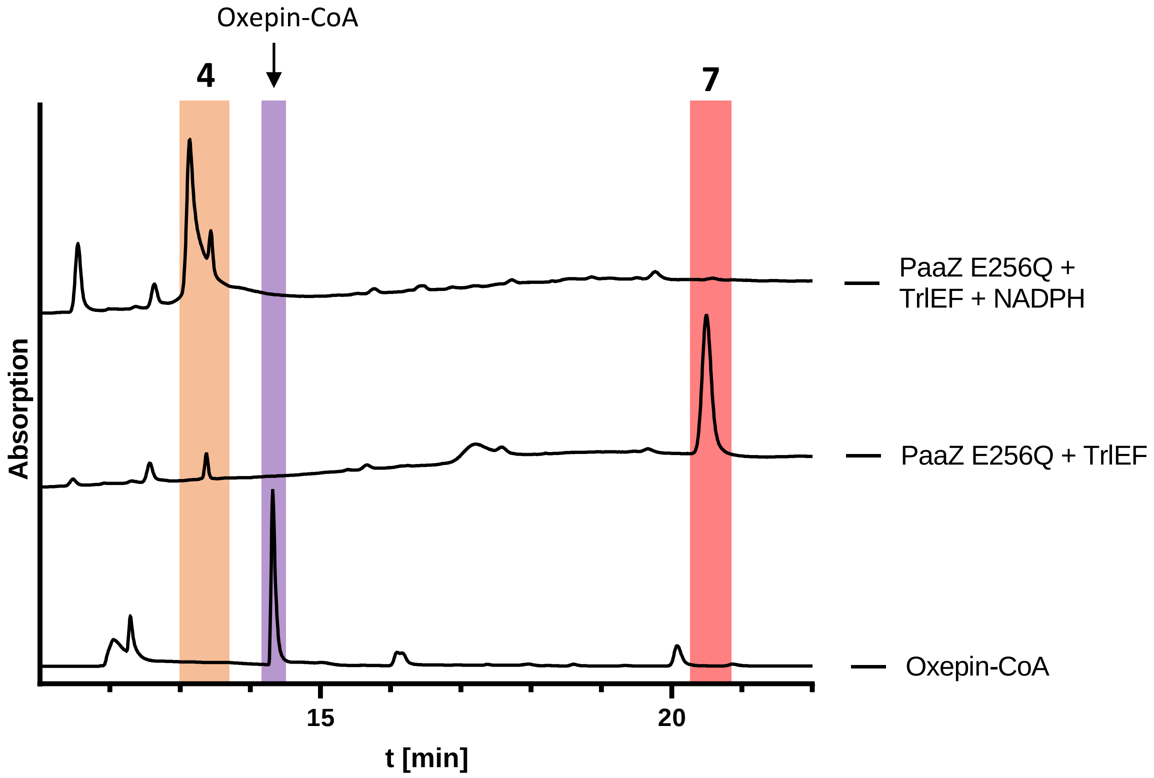

Figure S10 NADPH-dependency of the turnover of (TrlF-produced) 7 to tropolone (4) by TrlE. To investigate the need for NADPH as a cofactor for TrlE, oxepin-CoA was collected and used as a substrate in an assay with PaaZ E256Q, TrlF and TrlE. If no NADPH was added, only the formation of 7 by TrlF was observable. When NADPH was added to the assay, 4 was produced by TrlE. All samples were measured on a RP-HPLC system (see above in method section, 1.18 HPLC analysis), absorption was monitored at 280 nm (oxepin-CoA, highlighted in lila) and 300 nm (PaaZ E256Q + TrlEF, highlighted in red and PaaZ E256Q + TrlEF + NADPH, highlighted in orange). Since NADPH is also a cofactor for PaaABCE, it was necessary to test the NADPH dependency of TrlE in an assay were no PaaABCE was present, i.e. in an assay starting downstream of PaaABCE with PaaG-produced oxepin-CoA as substrate.

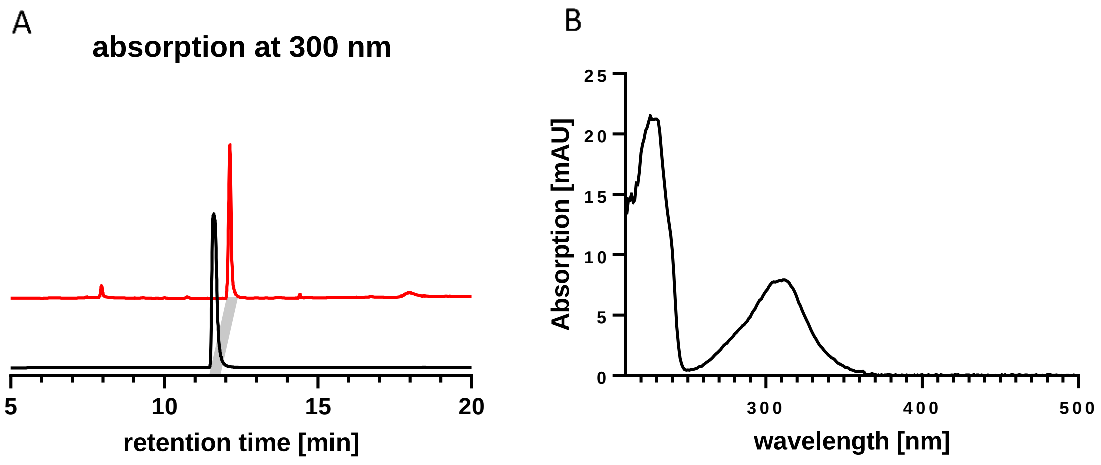

Figure S11 Incubation of tropone with TrlE. Commercially available tropone was incubated with TrlE and NADPH under shaking. (A) RP-HPLC analysis at 300 nm showing the identical retention time of the pure tropone standard (black) and the same peak from the assay (highlighted in red), which was not converted into any observable product within the time frame of the assay (15 min). (B) UV-visible spectrum of tropone with an absorption maximum around 312 nm.

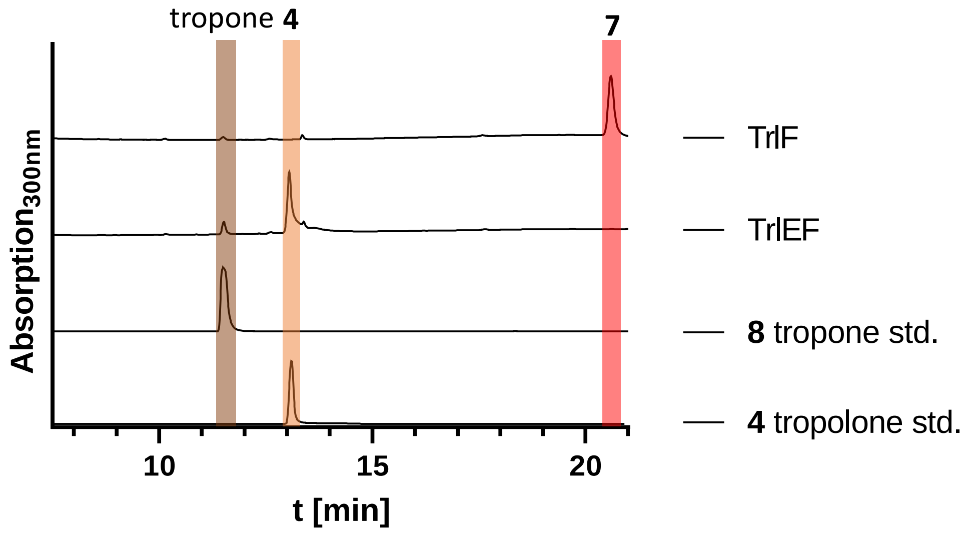

Figure S12 Comparison of tropone and tropolone standards to peaks from assay containing TrlE and TrlF. Verification of the formation of tropolone (4) as main product and tropone as side product by the TrlE-mediated conversion of compound 7 into compound 4. Samples were measured with the HPLC program for the semiprep nucleodur column (see above in method section, 1.18 HPLC analysis).

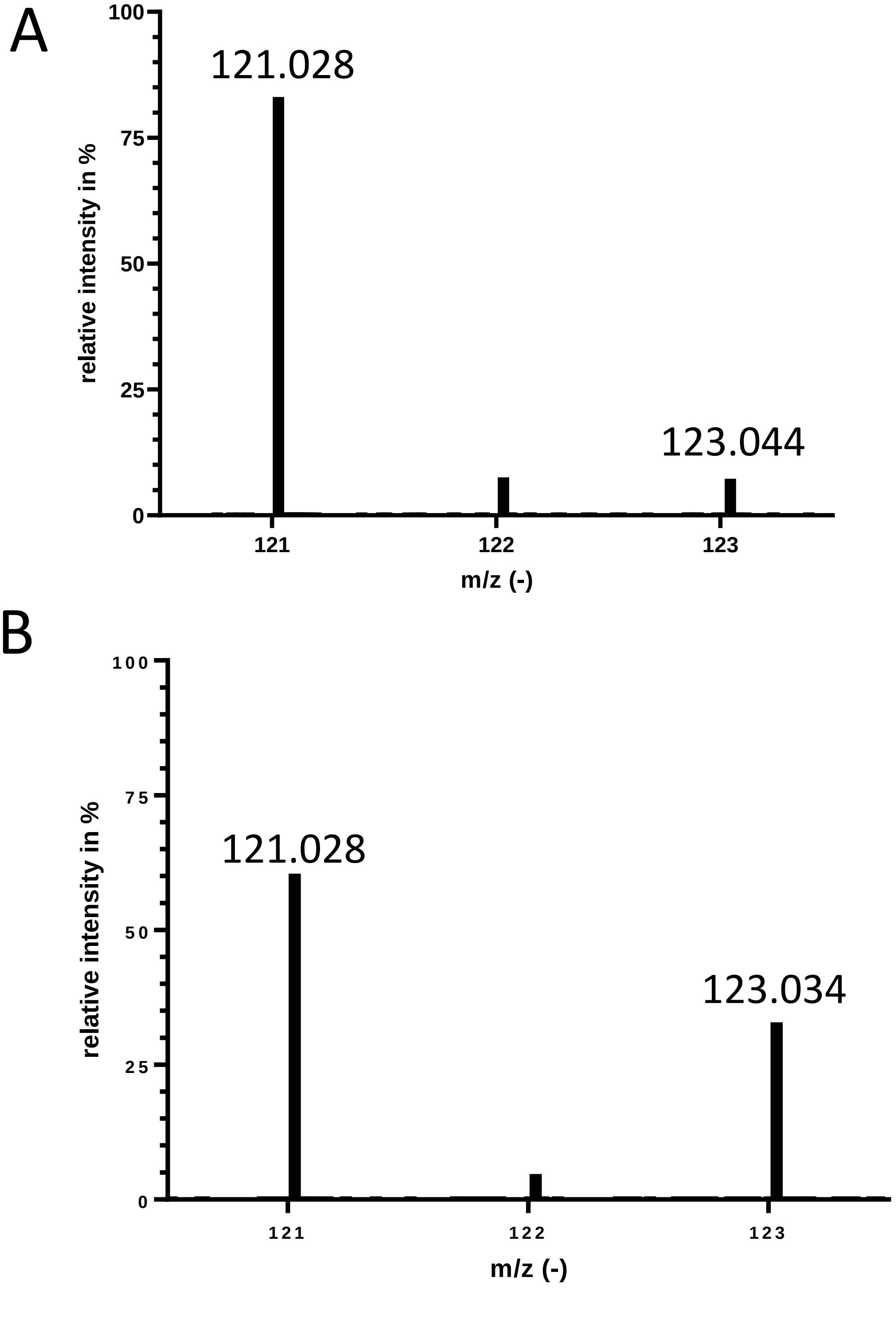

Figure S13 Incorporation of 18O labeled oxygen into tropolone (4) by TrlE. Distribution of masses (m/z (-)) for non-labeled oxygen that gets incorporated in tropolone by TrlE (A). The small peak at 123.044 is most likely background noise, as it does not fit the calculated mass of ^{18}O labeled tropolone of 123.0337. Distribution of masses for assay with ^{18}O-labeled oxygen (B). The ratio of 4 containing unlabeled or labeled oxygen is roughly 2:1.

```
TrlE      ----------------------MARKRPYVAVVGGGIGGLAVALGLRRQ-GVEAVVHE-Q  36
5EVY      MGS--SHHHHHHSSGDDD---DKSKSPLRVAVIGGGIAGTALALGLSKSSHVNVKLFE-T  54
4BJZ      ---------------------MSNLQDARIIIAGGGIGGAANALALAQK-GANVTLFE-R  37
5EOW      MGSDKIHHHHHHSSGENLYFQGHMRGRQKIAIVGAGLGGAAAATLLQQA-GFDVEVFE-Q  58
1DOD      -------------------------MKTQVAIIGAGPSGLLLGQLLHKA-GIDNVILERQ  34
                                         : : *.* .*   .  * :    :  : *

TrlE      AHA--LSHQGAGIAIGANGHRALRELGVAKRLTASAA-RPSRAD--FRHWRTGRSMVSHR  91
5EVY      APA--FGEIGAGVSFGVNAVEAIQRLGIGELYKSVADSTPAPWQDIWFEWRHAHDASLV-  111
4BJZ      ASE--FGEVGAGLQVGPHGARILDSWGVLDDVLSRAF-LP--KNIVFRDAITAEVLTKID  92
5EOW      APA--FTRLGAGIHIGPNVMKIFRRMGLEQKLELMGS-HPDFWF--SRDGNTGDYLSRIP  113
1DOD      TPDYVLGRIRAGVLE-QGMVDLLREAGVDRRMARDGLVHE-GVEIAFAGQRRRIDLKRLS  92
          :    : .  **:         :   *:       .

TrlE      LTGLYEERFGAPFWTVERAAVQQALLAELGP--RHVRL-GARCTGVDRTADGAVIRFEDG  148
5EVY      -GA--TVAPGIGQSSIHRADFIDMLEKRLPA--GIASL-GKHVVDYTENAEGVTLNFADG  165
4BJZ      LGSEFRGRYGGPYFVTHRSDLHATLVDAARAAGAELHT-GVTVTDVITEGDKAIVSTDDG  151
5EOW      LGEFARREYGAAYITIHRGDLHALQIEAIQP--GTVHF-GKRLEKIVDEGDQVRLDFADG  170
1DOD      -GGKTVTVYGQ-------TEVTRDLMEAREACGATTVYQAAEVRLHDLQGERPYVTFERD  144
                   *          .                  .         .:   :    .

TrlE      G---EAEADAVVGADGIHSAVRHSLFGP-----QEAVFSGTSGYRALVPMDRLRHVPEL-  199
5EVY      S---TYTADVAIAADGIKSSMRNTLLRAAGHDAVHPQFTGTSAYRGLVETSALREAYQAA  222
4BJZ      R---THEADIALGMDGLKSRLREKISGD------EPVSSGYAAYRGTTPYRDVE------  196
5EOW      T---HTVADIVIGADGIHSKIREELLGA-----EAPIYSGWVAHRALIRGVNLAQHADV-  221
1DOD      GERLRLDCDYIAGCDGFHGISRQSIPAERLKVFERVYPFGWLGLLADTPPVSHELIYA--  202
                 .*   . **::.  *. :              *  .  .

TrlE      -----AEPVLWLWLGPGRHFIAYPVADGSALNFLAVVPDRTWTV------ESWSTEGDAA  248
5EVY      SLDEHLLNVPQMYLIEDGHVLTFPVKKGKLIIIVAFVSDRSVAKPQWPSDQPWVRPATTD  282
4BJZ      -LDEDIED-VVGYIGPRCHFIQYPLRGGEMLNQVAVFESPGFKN----GIENW---GGPE  247
5EOW      -----FEPCVKWWSEDRHMMVYYTTGKRDEYYFVTGVPHEAWDF------QGAFVDSSQE  270
1DOD      -------------NHPRGFALCSQRSATRSRYYVQV--------PLSEKVEDWSDERFWT  241
                              :            :                :

TrlE      ELRAAFDGWHPFVTEVLG-ACERPGRWALYDREPQRVWSSGAVTLLGDAAHAMLPHHGQG  307
5EVY      EMLHRFAGAGEAVKTLLT-SIKSPTLWALHDFDPLPTYVHGRVALIGDAAHAMLPHQGAG  341
4BJZ      ELEQAYAHCHENVRRGID-YLWKDRWWPMYDREPIENWVDGRMILLGDAAHPPLQYLASG  306
5EOW      EMRAAFEGYHPTVQKLID-ATESITKWPLRNRNPLPLWSRGRLVLLGDACHPMKPHMAQG  329
1DOD      ELKARLPSE--VAEKLVTGPSLEKSIAPLRSF-VVEPMQHGRLFLAGDAAHIVPPTGAKG  298
          *:          .   :           : .         * : * ***.*      . *

TrlE      ANQALEDAVVLAHFLAR--T--DTGGVPSALRAYERLRRPRTRLLQAGSR-KNAGCFQLP  362
5EVY      AGQGLEDAYFMAELLGNPLH--EASDIPALLEVYDDVRRGRASKVQLTSR-EAGELYEYR  398
4BJZ      AVMAIEDAKCLADYAAEDFSTGGNSAWPQILKEVNTERAPRCNRILTTGR-MWGELWHLD  365
5EOW      ACMAIEDAAMLTRCLQE--T--GLSDHRTAFALYEANRKERASQVQSVSN-ANT------  378
1DOD      LNLAASDVSTLYRLLLKAYREGR----GELLERYSAICLRRIWKAERFSWWMTSVLHRFP  354
             . .*.  :     .             :   .     *       .

TrlE      DGPQAEARNA----RLATLPDDVAWIHGHDILGSLPVATSPA------------------  400
5EVY      TPGVERDTAK----LKALLESRMNWIWNYDLGAEARLAVKPAL---A-------------  438
4BJZ      GTARI-ARNELFRTRDTSSYKYTDWLWGYSSDRASKLGPEQKL---ISEEDLNSAVDHHH  421
5EOW      ---------------WLYSQEDPAWVYGYDLYGQQLESGEAA------------------  405
1DOD      DTDAF-------------SQRIQQTELEYYLGSEAGLATIAENYVGLPYEEIE-------  394
                                      :        .

TrlE      ---400
5EVY      ---438
4BJZ      HHH424
5EOW      ---405
1DOD      ---394
```

Figure S14 Multiple sequence alignment of TrlE with known FAD-dependent group A monooxygenases. The GD-fingerprint, a sequence motif for FAD-binding conserved in this class of enzymes, is highlighted in yellow. Likely catalytic active residues of TrlE are highlighted in red. The alignment was generated with ClustalOmega, default parameter setting were used.

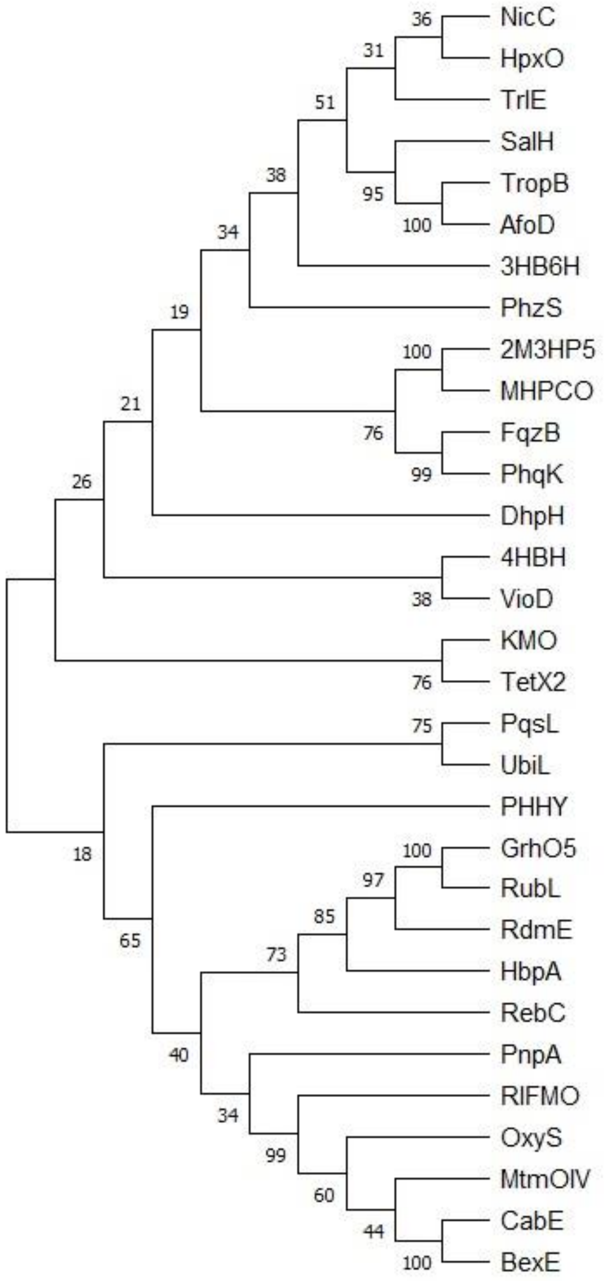

Figure S15 Phylogenetic distance tree of TrlE and other group A flavoprotein monooxygenases. The tree with indicated bootstrap values (1,000 replicates) was conducted with the Mega 11 software[6] using Jones-Taylor-Thornton (JTT) matrix based model and maximum likelihood method. Amino acid sequences were aligned using the ClustalW algorithm. Shown are members from the FAD-dependent group A monooxygenases with solved crystal structure (for PDB codes and accession numbers see table S2).

Table S2 TrlE and homologues enzymes from the phylogenetic distance tree with according PDB code, Uniprot Accession Nr. and species

Enzyme	PDB code	Species	Uniprot Accession Nr.
**NicC**	5EOW	*Pseudomonas putida*	Q88FY2
**HpxO**	3RP6	*Klebsiella pneumoniae*	A6T923
**TrlE**	8RQH	*Streptomyces cyaneofuscatus*	A0A2S1JZI9
**SalH**	5EVY	*Pseudomonas putida*	Q59713
**TropB**	6NES	*Talaromyces stipitatus*	B8M9J8
**AfoD**	7LO1	*Aspergillus nidulans*	Q5BEJ7
**3HB6H**	4BJZ	*Rhodococcus jostii*	Q0SFK6
**PhzS**	2RGJ	*Pseudomonas aeruginosa*	Q9HWG9
**2M3HP5**	3GMC	*Mesorhizobium loti*	Q988D3
**MHPCO**	4GF7	*Mesorhizobium japonicum*	Q988D3
**FqzB**	7CP6	*Aspergillus fumigatus*	A0A0J5T0B0
**PhqK**	6PVI	*Penicillium fellutanum*	L0E4H0
**DhpH**	2VOU	*Paenarthrobacter nicotinovorans*	Q93NG3
**4HBH**	1DOD	*Pseudomonas aeruginosa*	P20586
**VioD**	3C4A	*Chromobacterium violaceum*	Q9S3U8
**KMO**	5NAK	*Pseudomonas fluorescens*	Q84HF5
**TetX2**	3P9U	*Bacteroides thetaiotaomicron*	Q93L51
**PqsL**	6FHO	*Pseudomonas aeruginosa*	Q9HWJ1
**UbiL**	4K22	*Escherichia coli*	P25535
**PHHY**	1PN0	*Cutaneotrichosporun cutaneum*	P15245
**GrhO5**	7OUC	*Streptomyces sp. JP95*	Q8KSX7
**RubL**	7OUJ	*Streptomyces collinus*	Q8KY42
**RdmE**	3IHG	*Streptomyces purpurascens*	Q54530
**HbpA**	4CY8	*Pseudomonas nitroreducens*	O06647
**RebC**	2R0P	*Lechevalieria aerocolonigenes*	Q8KI25
**PnpA**	6AIO	*Pseudomonas putida*	C6FI48
**RIFMO**	5KOW	*Nocardia farcinica*	Q5YTV5
**OxyS**	4K2X	*Streptomyces rimosus*	L8EUQ6
**MtmOIV**	3FMW	*Streptomyces argillaceus*	Q194P4
**CabE**	2QA2	*Streptomyces sp.*	D0VWY3
**BexE**	4X4J	*Amycolatopsis orientalis*	D7RFJ3

Table S3 Data collection and refinement statistics from the crystal structure of TrlE. Statistics for the highest-resolution shell are shown in brackets**.**

PDB ID	8RQH
Beam line	Swiss Light Source X06SA
Wavelength (Å)	1.00003
Space group	P 3_1 2 1
Unit cell	
a, b, c (Å)	112.9, 112.9, 148.57
α, β, γ (°)	90, 90, 120
Resolution range (Å)	49.52 - 2.5 (2.589 - 2.5)
R_{merge}	0.2156 (2.813)
Mean I / σ(I)	14.02 (0.92)
$CC_{1/2}$	0.999 (0.554)
Completeness (%)	99.93 (99.87)
Multiplicity	20.6 (20.5)
R_{work}	0.2041 (0.3766)
R_{free}	0.2419 (0.4270)
Average B-factors	
Protein	67.74
Ligands	64.99
Solvent	56.98
RMSD	
Bonds (Å)	0.008
Angle (°)	1.47
Ramachandran plot	
In favored regions	97.22
In allowed regions	2.78
Outliers	0

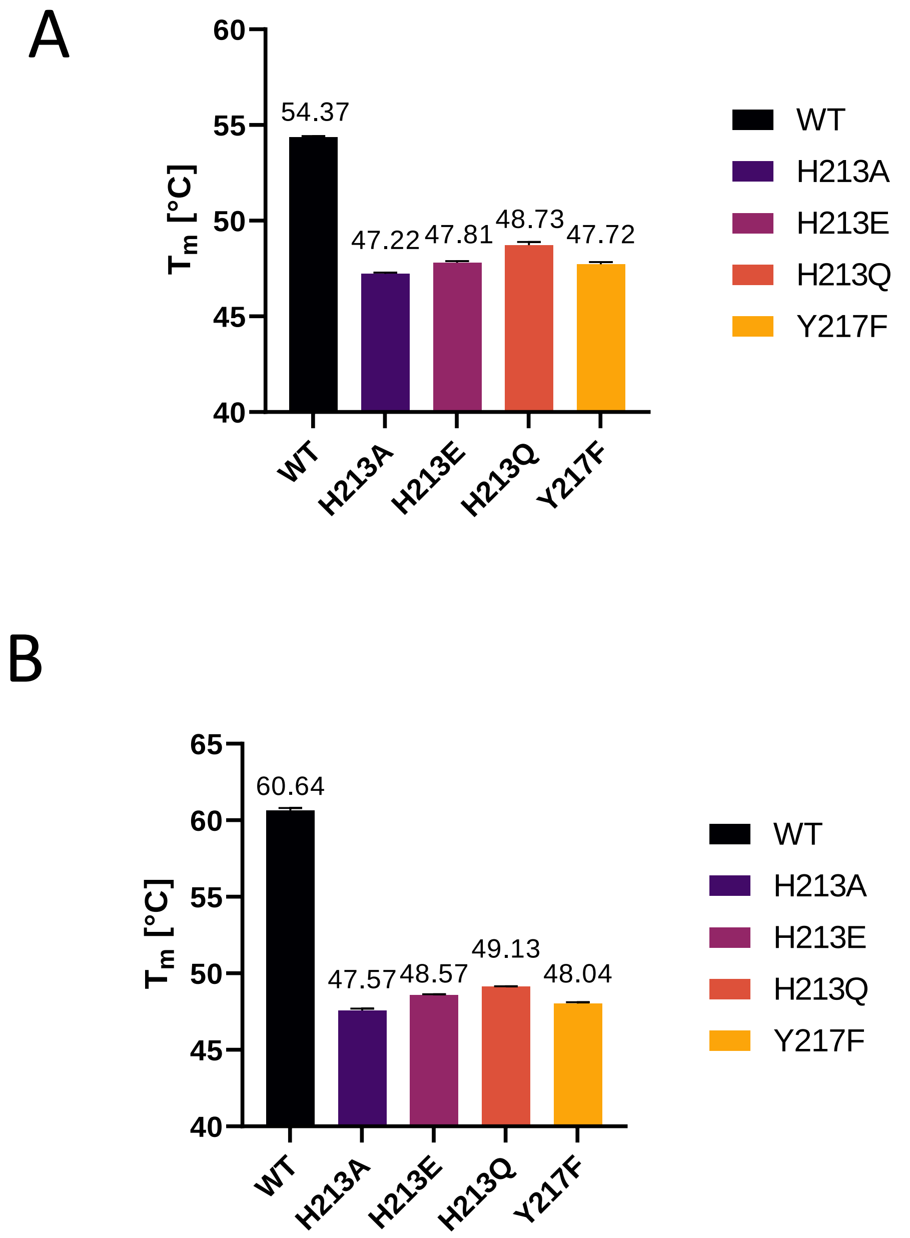

Figure S16 Determination of the melting temperature (T_m) of TrlE and variants in the absence (A) and presence of 1 mM salicylate or B) 50 µM FAD. Values were obtained by performing NanoDSF measurements with 10 µM enzyme in triplicates.

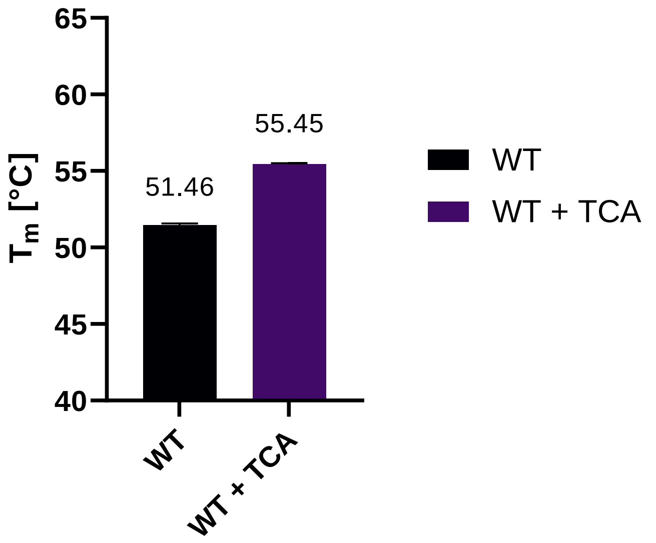

Figure S17 Effect of tropone-2-carboxylic acid (TCA) on the melting temperature (T_m) of TrlE. The melting temperature of 10 µM TrlE with and without the addition of 1 mM TCA was determined by NanoDSF in triplicates. No additional FAD was added in these measurements.

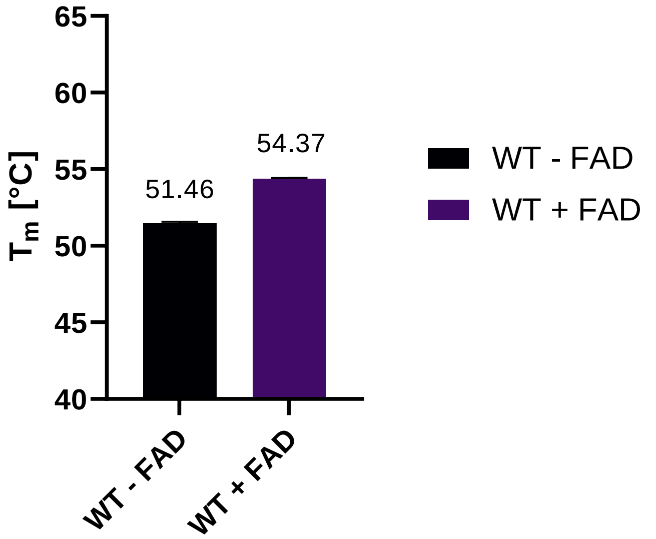

Figure S18 Effect of FAD on the melting temperature (T_m) of TrlE. The melting temperature of 10 µM TrlE with and without the addition of 50 µM FAD was determined by NanoDSF in triplicates

Table S4 Melting temperatures (TM) and standard deviation (SD) in °C of TrlE and its variants. Measured in triplicates on Prometheus NanoDSF device, with increments of 1.5°C per minute (ranging from 25°C to 90°C).

Sample	*- salicylate*		*+ salicylate*	
	**T_M**	**SD**	**T_M**	**SD**
WT	54.37	0.06	60.64	0.16
H213A	47.22	0.07	47.57	0.13
H213E	47.81	0.08	48.57	0.06
H213Q	48.73	0.16	49.13	0.03
Y217F	47.72	0.12	48.04	0.08

Sample	*- FAD*		*+ FAD*	
	**T_M**	**SD**	**T_M**	**SD**
WT	51.46	0.11	54.37	0.06

Sample	*- TCA*		*+ TCA*	
	**T_M**	**SD**	**T_M**	**SD**
WT	51.46	0.11	55.45	0.06

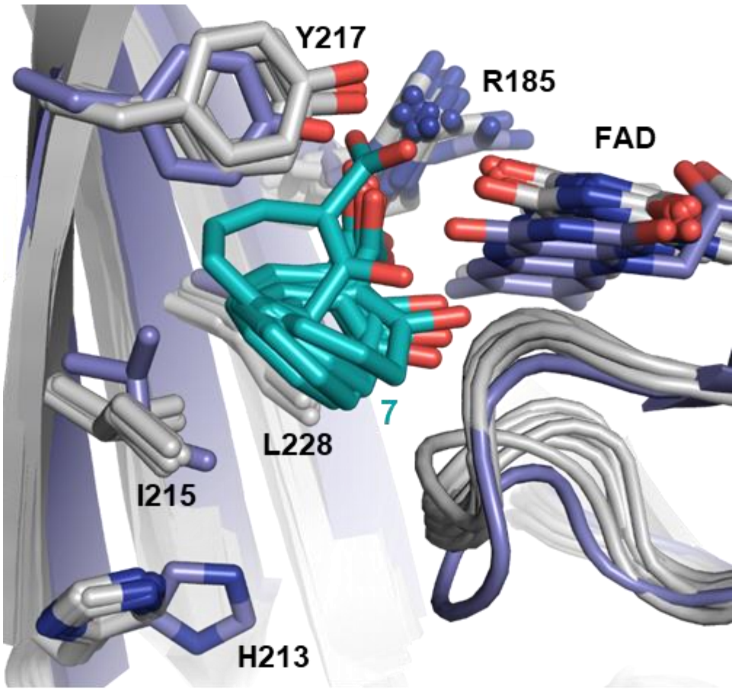

Figure S19 Representative poses of 7 **in the active site of TrlE.** Ten representative structures of **7** in the active site of TrlE after molecular dynamics simulation, clustering of the MD trajectory and energy minimization of the cluster representatives are shown. **7** is shown in teal and the corresponding TrlE and FAD after molecular dynamics simulation and minimization are shown in white. The simulated structures are aligned to the crystal structure of TrlE (shown in blue).

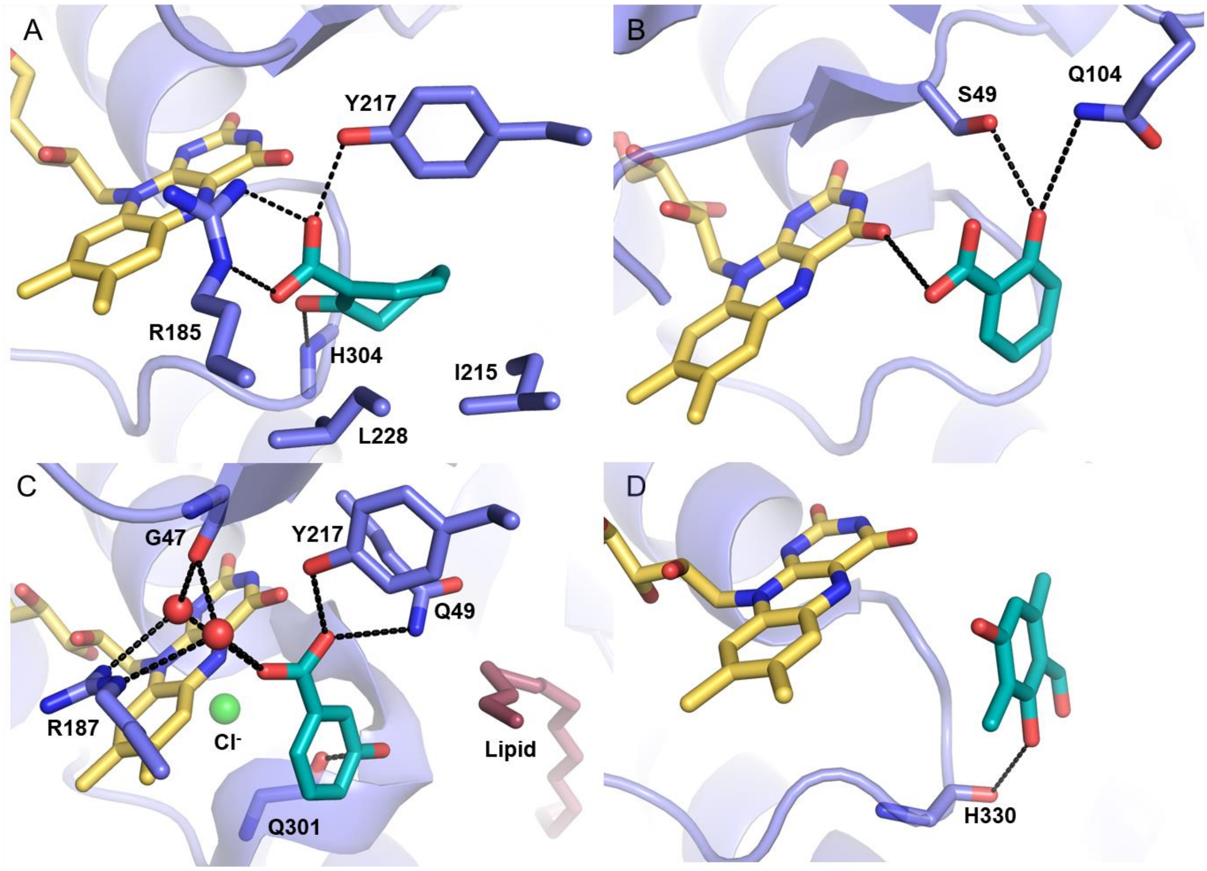

Figure S20 Comparison of the docked pose of 7 **in the active site of TrlE with crystal structures of homologs in complex with their native substrates.** (**A**) Pose obtained after docking, molecular dynamics simulation, clustering and minimization of representative of largest cluster of **7** in the active site of TrlE (representative structure). (**B**) Crystal structure of salicylate hydroxylase (SalH) with bound salicylate; PDB ID: 5EVY. (**C**) Crystal structure of 3-hydroxybenzoate 6-hydroxylase with bound 3-hydroxybenzoate; PDB ID: 4BK1. (**D**) Crystal structure of TropB with bound 2,4-dihydroxy-3,6-dimethylbenzaldehyde; PDB ID: 6NET. Hydrogen bonds are displayed as black dashes (donor-acceptor distance below 3.5 Å). FAD is shown in yellow, waters are shown as red spheres and the respective substrates are shown in teal**.**

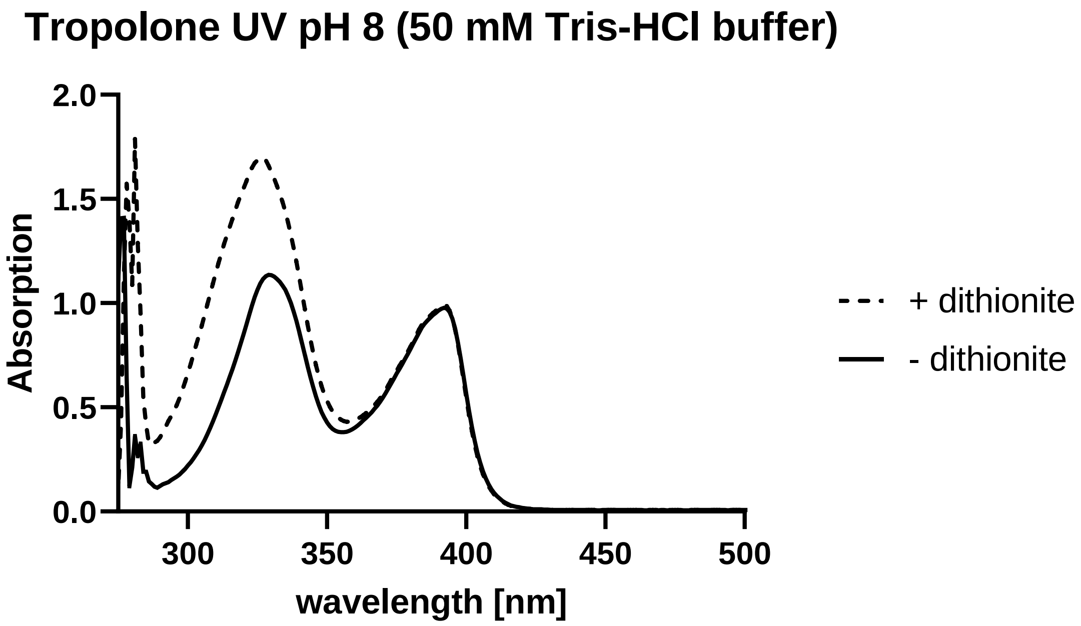

Figure S21 UV-visible spectra of tropolone (4) without (solid line) and with dithionite (dashed line) in 50 mM Tris-HCl pH 8 buffer.

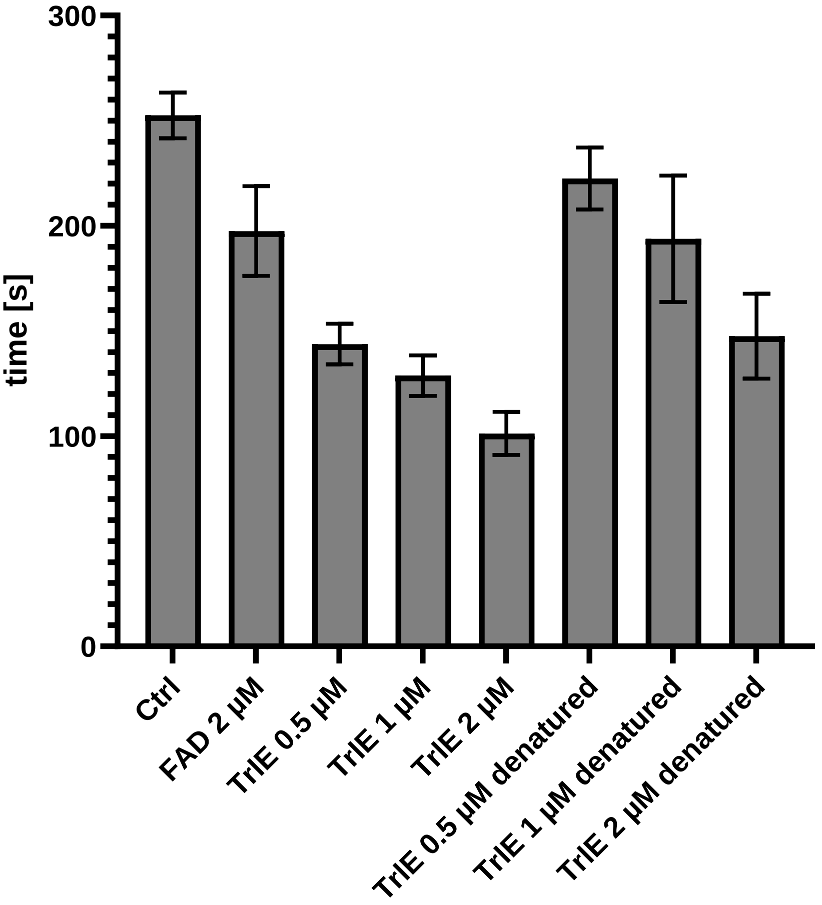

Figure S22 TrlE mediated re-oxidation of tropolone. Changes in absorption of 25 µM tropolone upon addition of 200 µM sodium dithionite were measured at 340 nm. The time it took to reach baseline level of absorption was measured as a control. Varying amounts of TrlE were added (0.5, 1 and 2 µM) and measured (N = 4). As additional controls, the experiment was repeated with the addition of FAD (2 µM) or heat-denatured TrlE (0.5, 1 and 2 µM) to the tropolone solution.

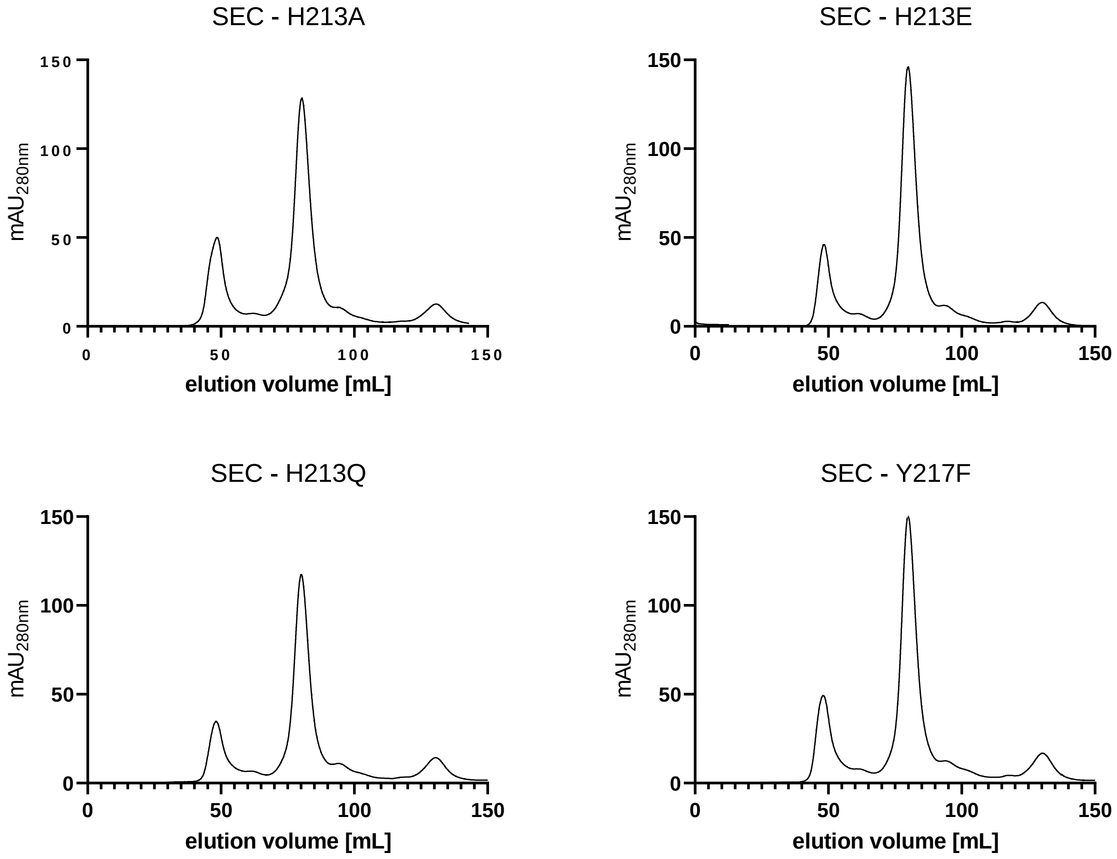

Figure S23 SEC of TrlE variants. Analytical gel-filtration of 6xHis-GB1-tagged TrlE Variants. All variants show a major peak at around 80 mL elution volume indicating the presence of the proteins as monomers (~52 kDa). The peaks at 50 mL elution volume correspond to aggregates and the ones at 130 mL to co-purified impurities from affinity chromatography.

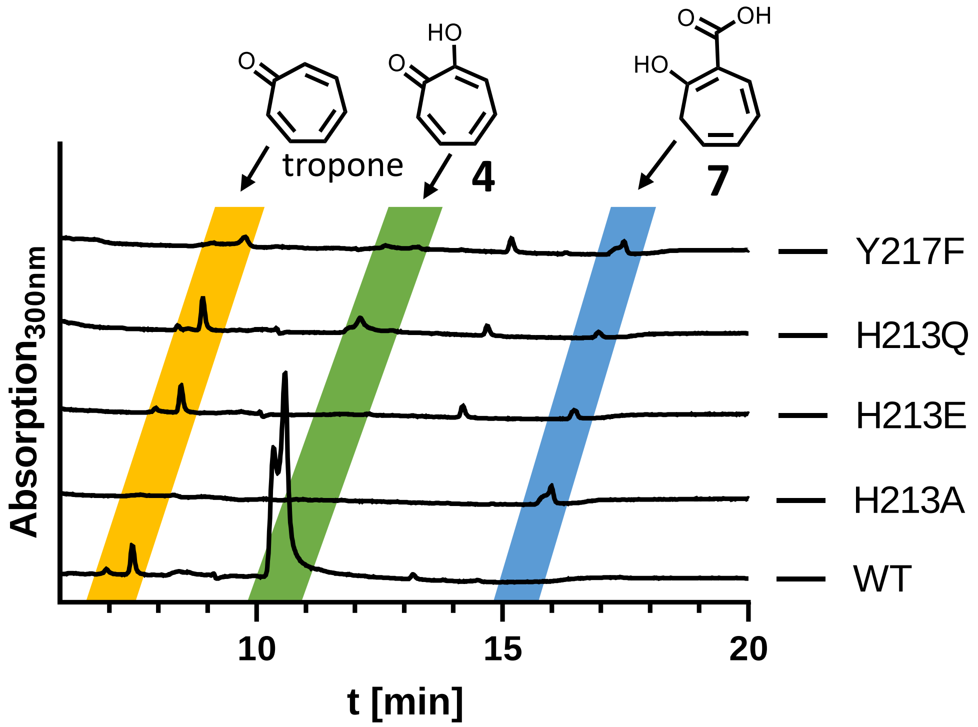

Figure S24 RP-HPLC analysis (300nm) of the conversion of 7 (highlighted in blue) into 4 (green) by TrlE WT and different variants. In order to investigate the role of different amino acids in the active site of TrlE, assays with the wild type enzyme and variants were conducted as described in 1.23. Exceptions during handling were using 2.5 µM of each enzyme and setting the incubation to 10 minutes. Samples were subsequently quenched with ethyl acetate + 1 % formic acid, before being dried under N_2 gas flow and resuspended in acetonitrile and water. Small amounts of formed product **4** could be observed for the variants H213Q and Y217F. Tropone could be observed as a side-product in all variants and the wild type except for variant H213A. Full substrate (**7**) conversion in the observed timeframe was only noted for the wild type TrlE. Samples were measured with the HPLC program for the analytical SunFire column (see above in method section, 1.19 LC-MS analysis).

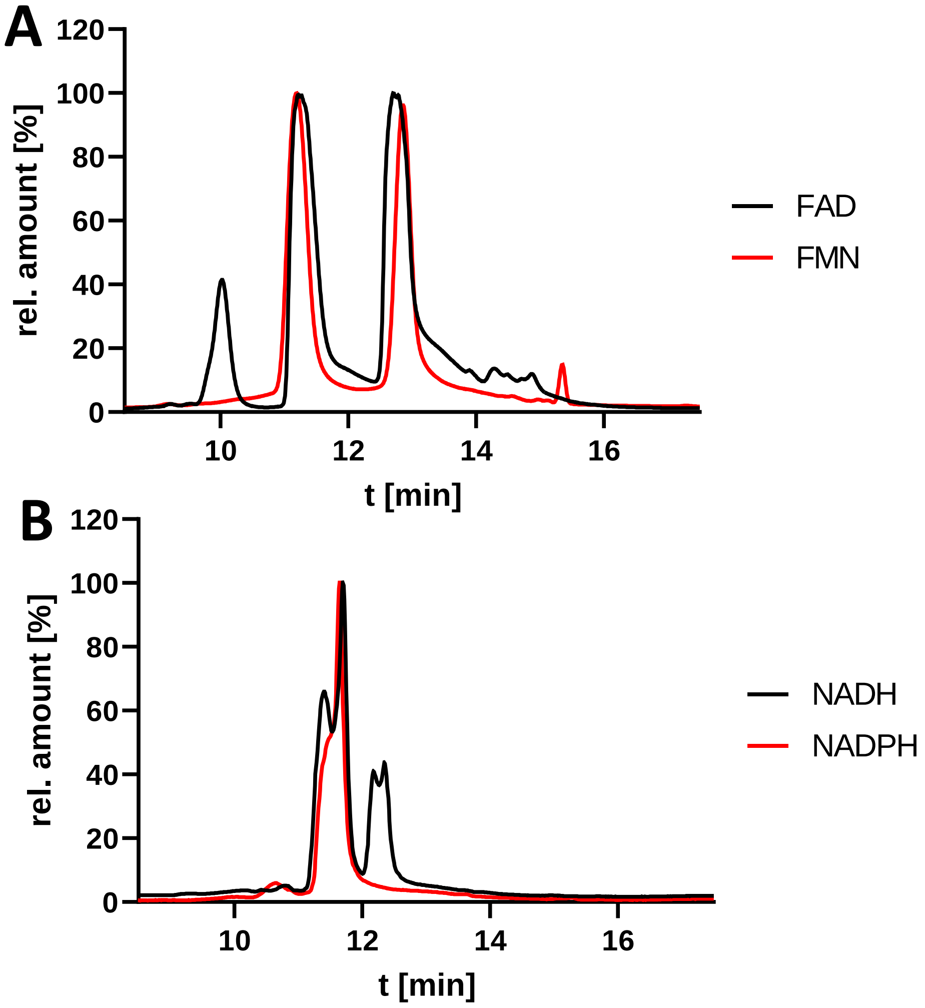

Figure S25 Cofactor preferences of TrlD. (**A**) Comparison of FAD and FMN usage of TrlCD in an assay with NADH regeneration system. The samples were prepared as described in 1.25, one time with FAD and one time with FMN. Incubation time was set to 2 h and extraction was carried out as described. The samples were analyzed on the semi-preparative HPLC using the XBridge column (see HPLC analysis, 1.17) Only when FAD was used in the assay, a peak corresponding to compound **6** could be observed. (**B**) Comparison of NADH and NADPH usage of TrlCD in an assay using FAD as Flavin cofactor. The samples were prepared as described in 1.25 with the exception of not using components for the NADH/NADPH regeneration system (sodium formate, formate dehydrogenase and catalase). Incubation time was set to 10 min and extraction was carried out as described. The samples were analyzed on the shimadzu LC-MS system using water and acetonitrile as mobile phase (see 1.19). Only in the sample with NADH a peak corresponding to compound **5** could be observed.

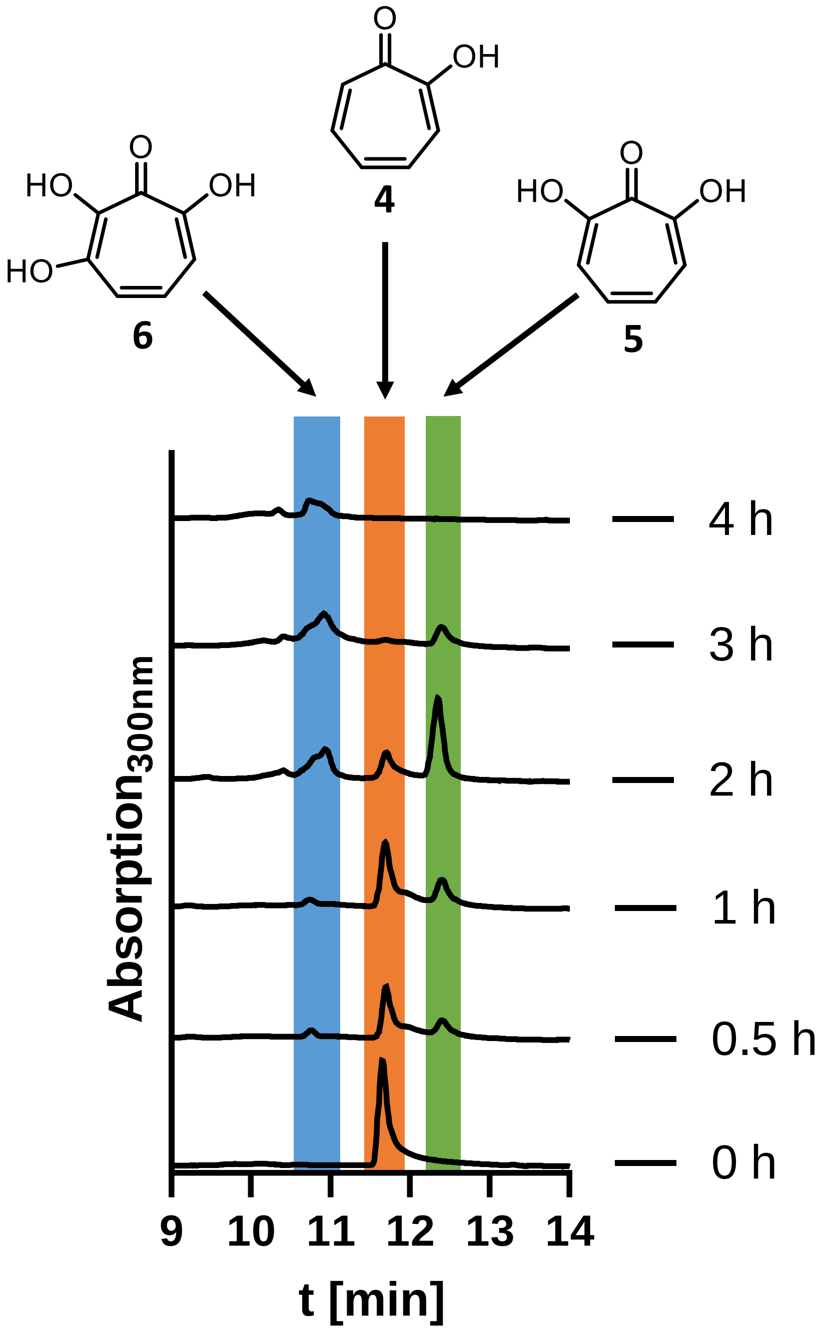

Figure S26 RP-HPLC analysis (300 nm) of 7-hydroxytropolone (5) and 3,7-dihydroxytropolone (6) formation catalyzed by TrlCD over time. Samples were drawn at 0, 0.5, 1, 2, 3 and 4 hours. An NADH cofactor regeneration system consisting of formate dehydrogenase, sodium formate and catalase was used. Without cofactor regeneration system, only traces of 6 could be observed, even with prolonged incubation. Samples were measured with the HPLC program for the analytical SunFire column (see above in method section, 1.19 LC-MS analysis, which causes different retention times compared to the method for the semiprep nucleodur column).

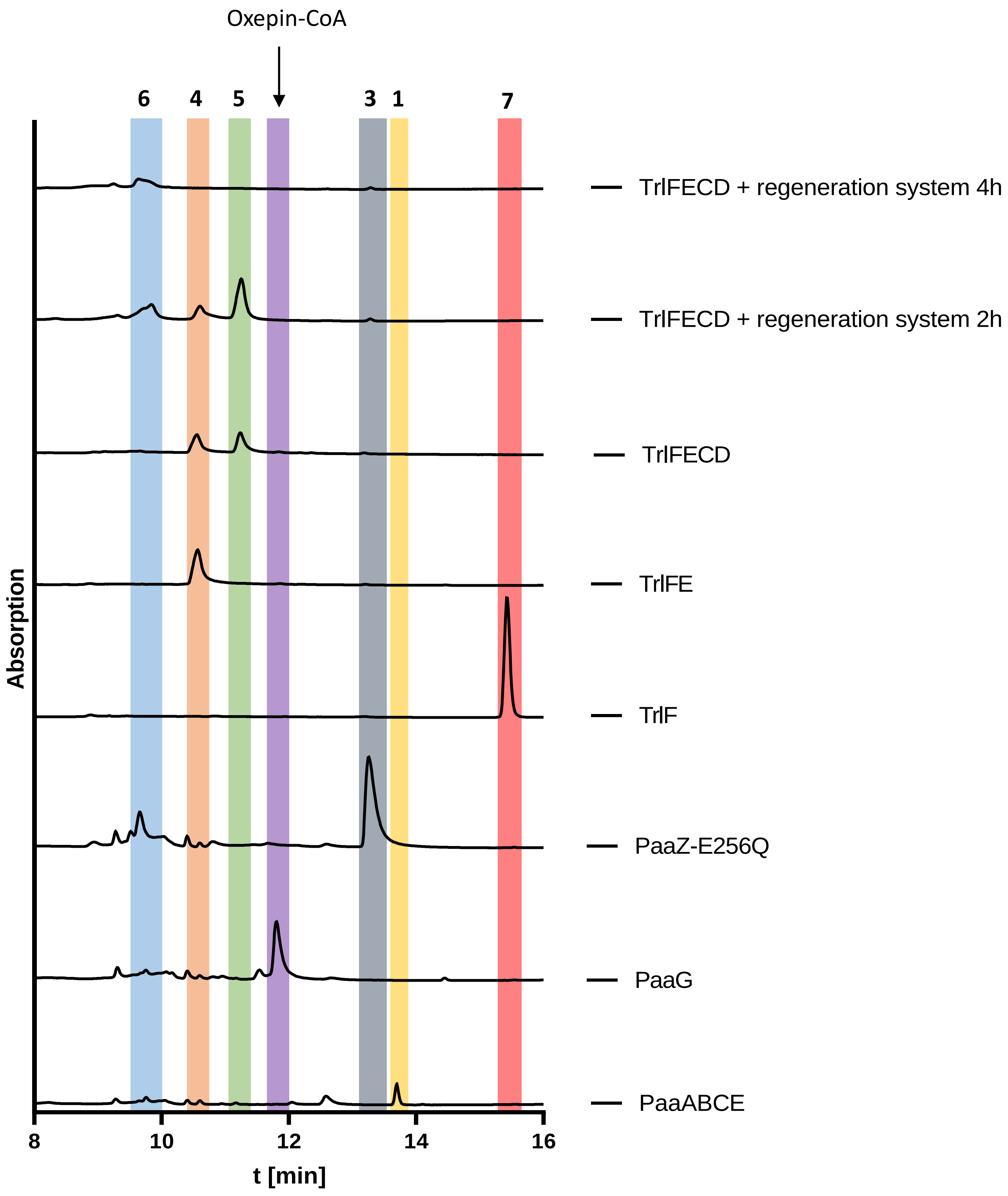

Figure S27 Complete 3,7-dihydroxytropolone (6) biosynthesis pathway reconstituted. UV traces (280 nm for PaaABCE – TrlF and 300 nm TrlFE – TrlFECD + regeneration system 4h) of RP-HPLC analysis of all involved enzymes in **6** biosynthesis. Samples in lines PaaABCE, PaaG and PaaZ-E256Q were obtained by stopping assays with MeOH, vortexing, centrifuging and drying them in a speedvac. HPLC analysis was done with program 1 (method with buffer containing ammonium acetate, see method section above). All other samples were obtained by extracting assays with ethylacetate + 0.1 % formic acid, vortexing, centrifuging and drying them under N2 flow. Samples were measured with the HPLC program for the analytical SunFire column (see above in method section, 1.19 LC-MS analysis).

Table S5 HRMS-measurements and calculated masses for compounds 4, 5 and 6

Compound	Sum formula	Calculated mass [M-H$^+$]	Measured mass [M-H$^+$]
**4**	$C_7H_6O_2$	121.029	121.028
**5**	$C_7H_6O_3$	137.024	137.023
**6**	$C_7H_6O_4$	153.019	153.019

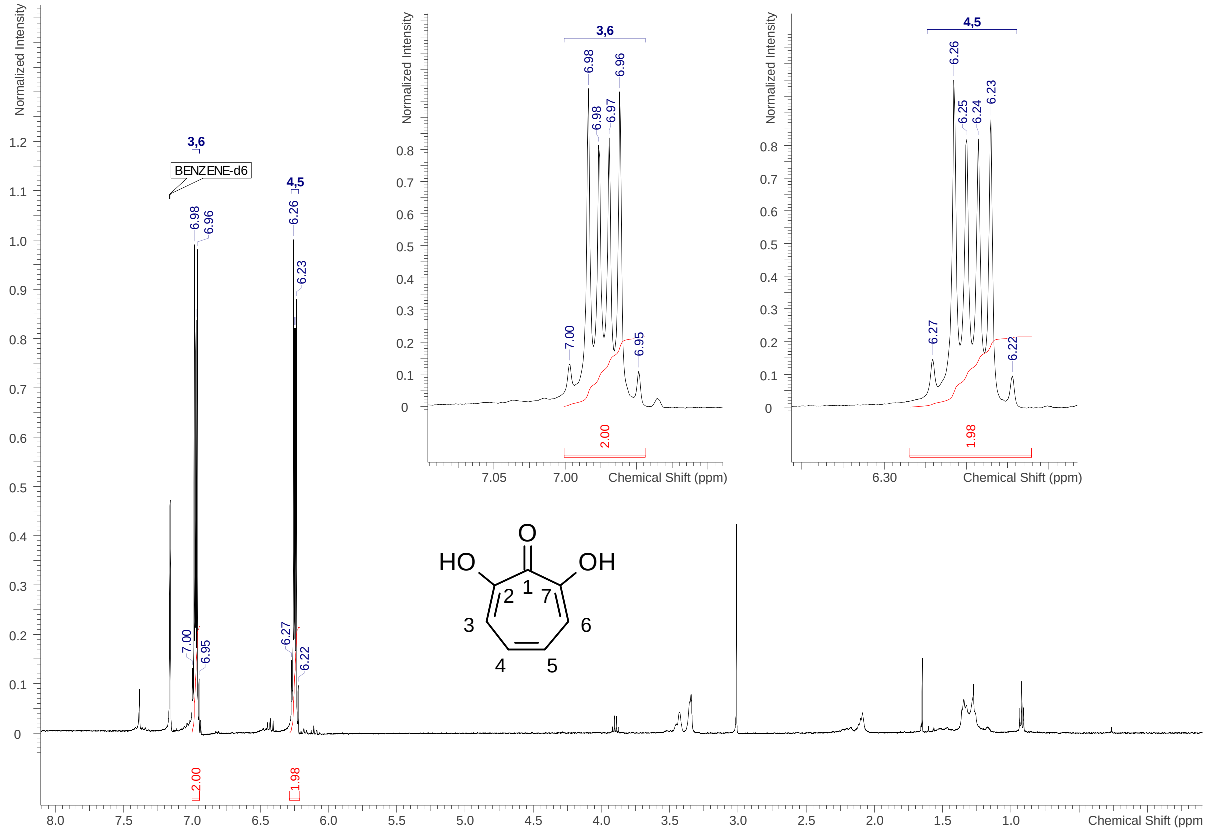

Figure S28 1H NMR spectrum of 5 **(500 MHz, C6D6)**.

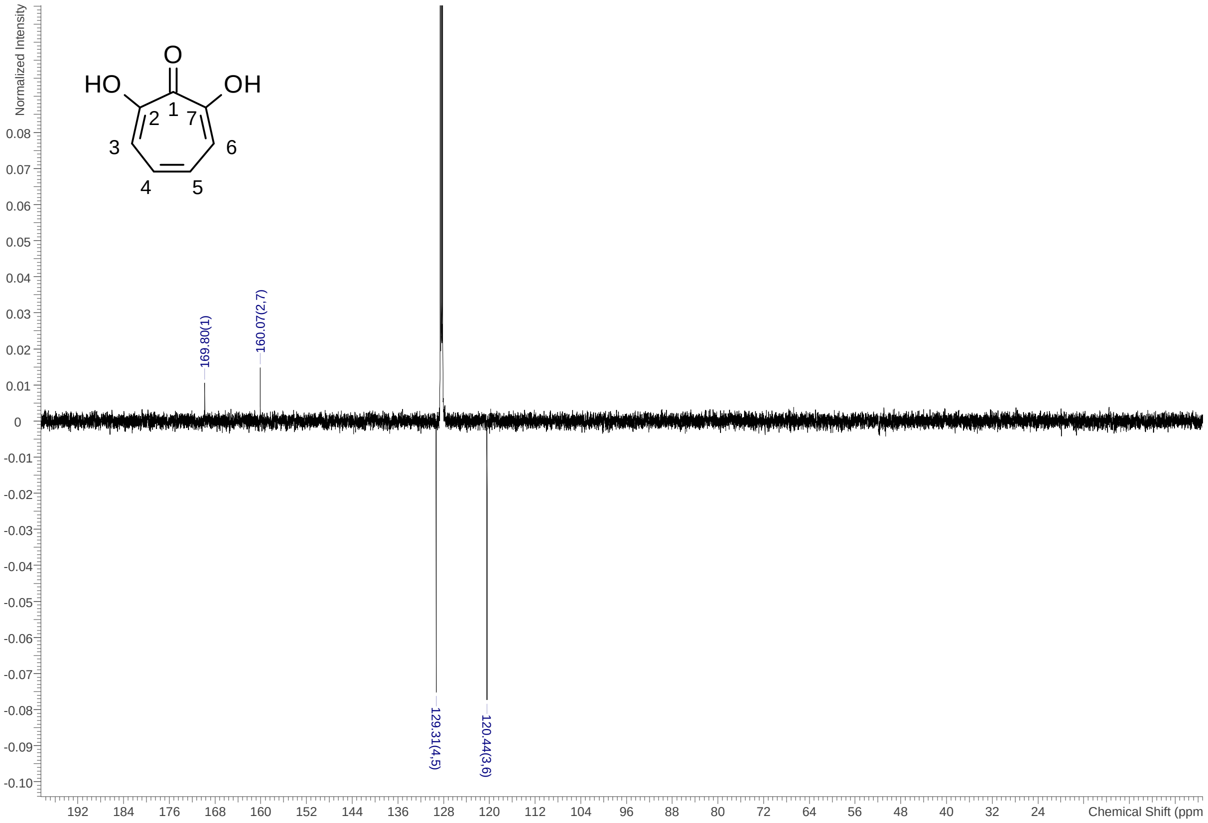

Figure S29 ^{13}C NMR spectrum of 5 **(126 MHz, C_6D_6).**

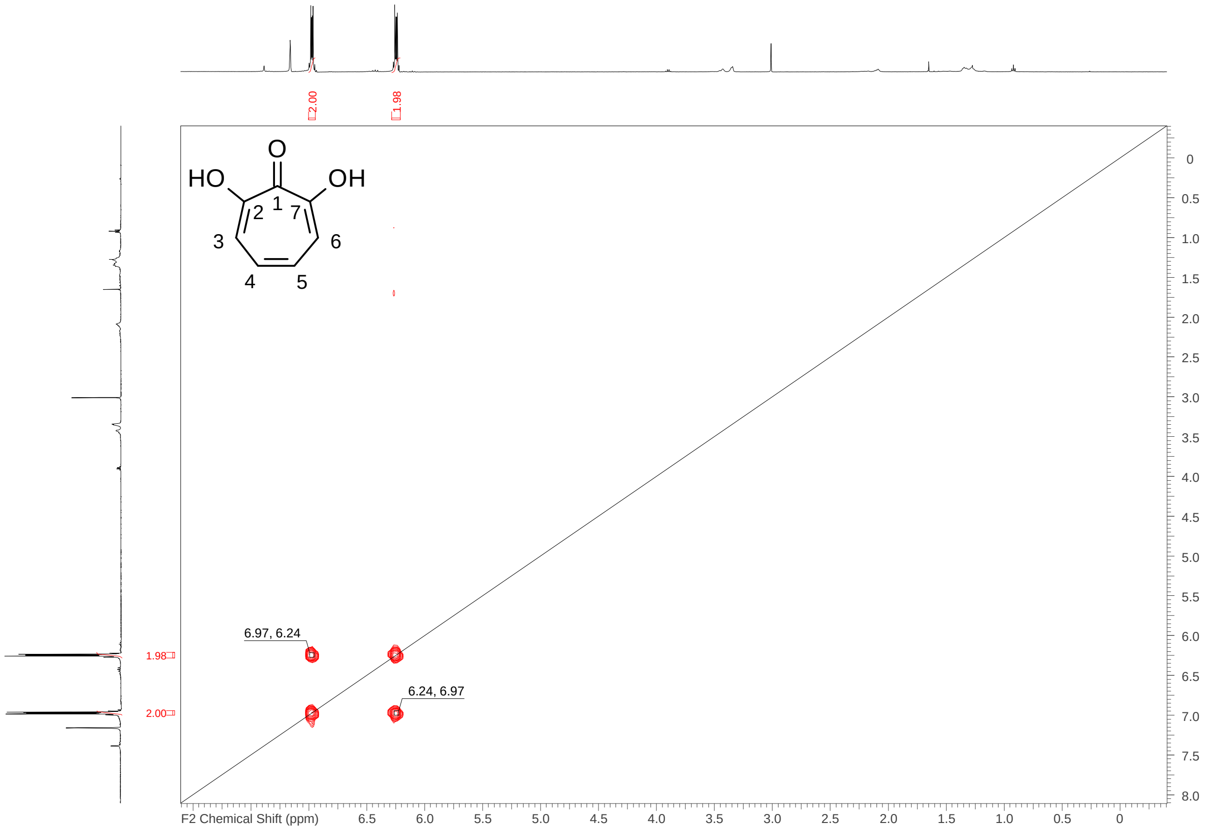

Figure S30 COSY NMR spectrum of 5 **(500 MHz, C_6D_6).**

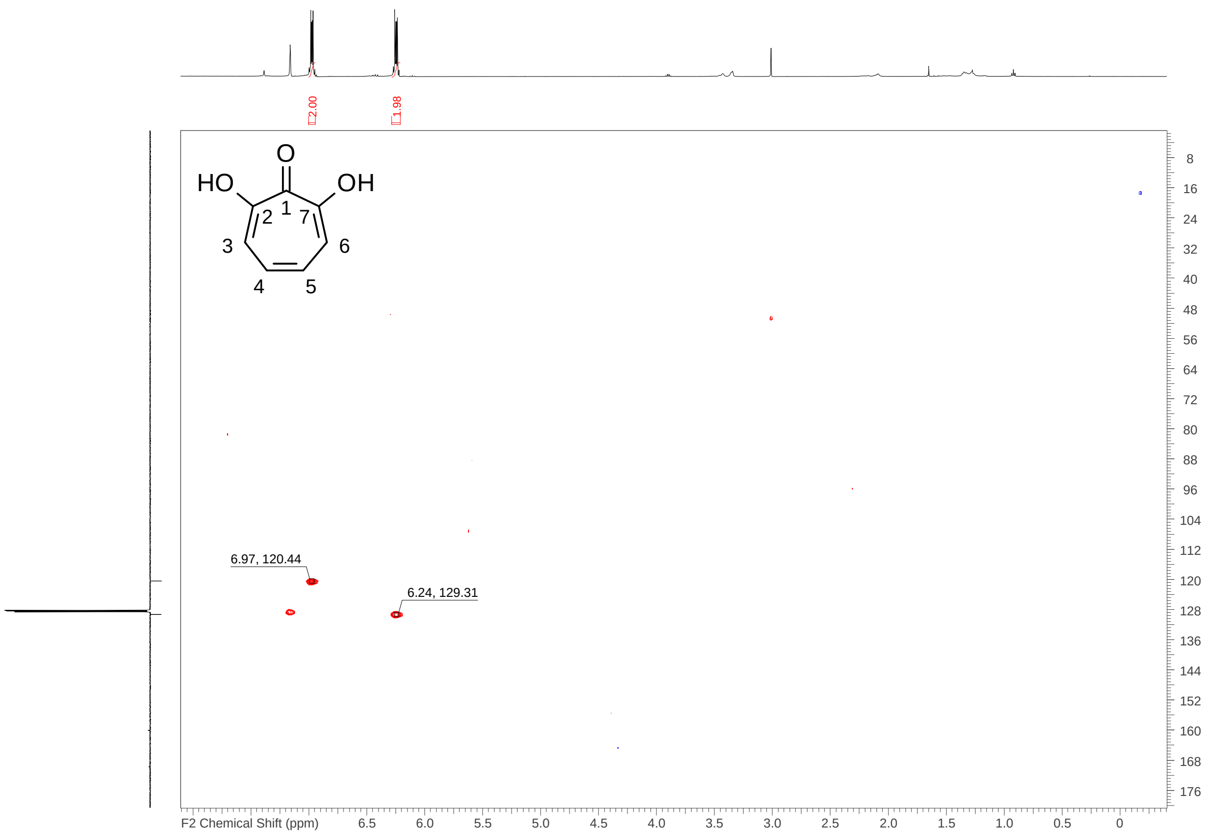

Figure S31 HSQC NMR spectrum of 5 (500 MHz, C_6D_6).

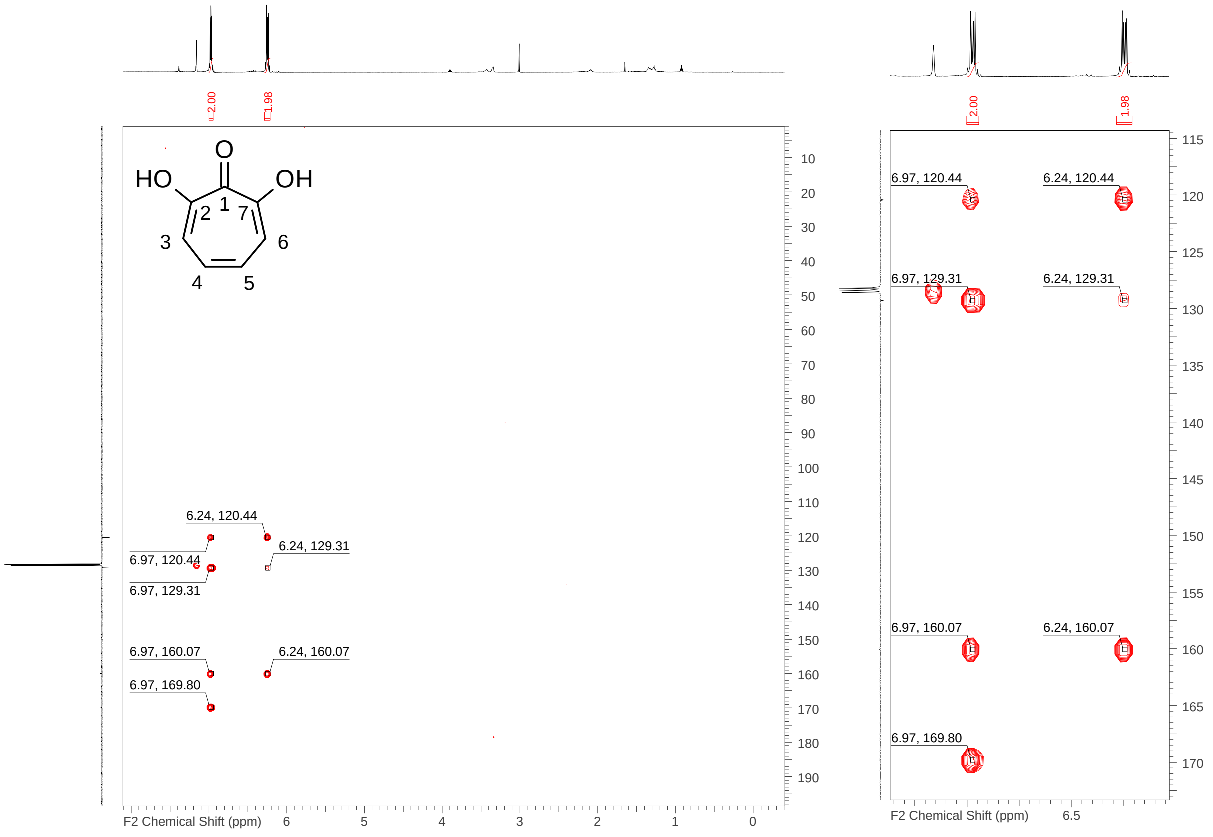

Figure S32 HMBC NMR spectrum of 5 (500 MHz, C_6D_6).

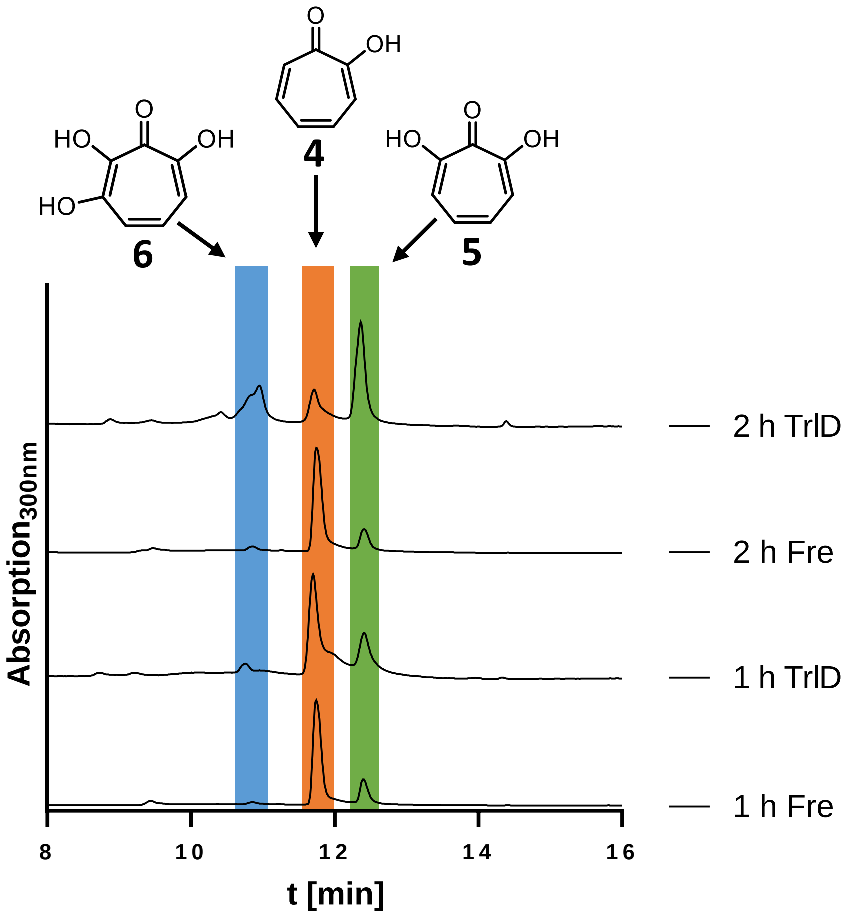

Figure S33 Comparison of the conversion of 4 into 5 and 6 by TrlCD and TrlC + Fre including cofactor regeneration system. RP-HPLC analysis of the conversion of 4 in different assays. Shown are two time points of an assay containing TrlC and its native reductase partner TrlD (1 h and 2 h) and two time points from the same assay with TrlD substituted by Fre from *E. coli* (after 1 h and 2 h respectively). Samples were measured with the HPLC program for the analytical SunFire column (see above in method section, 1.19 LC-MS analysis).

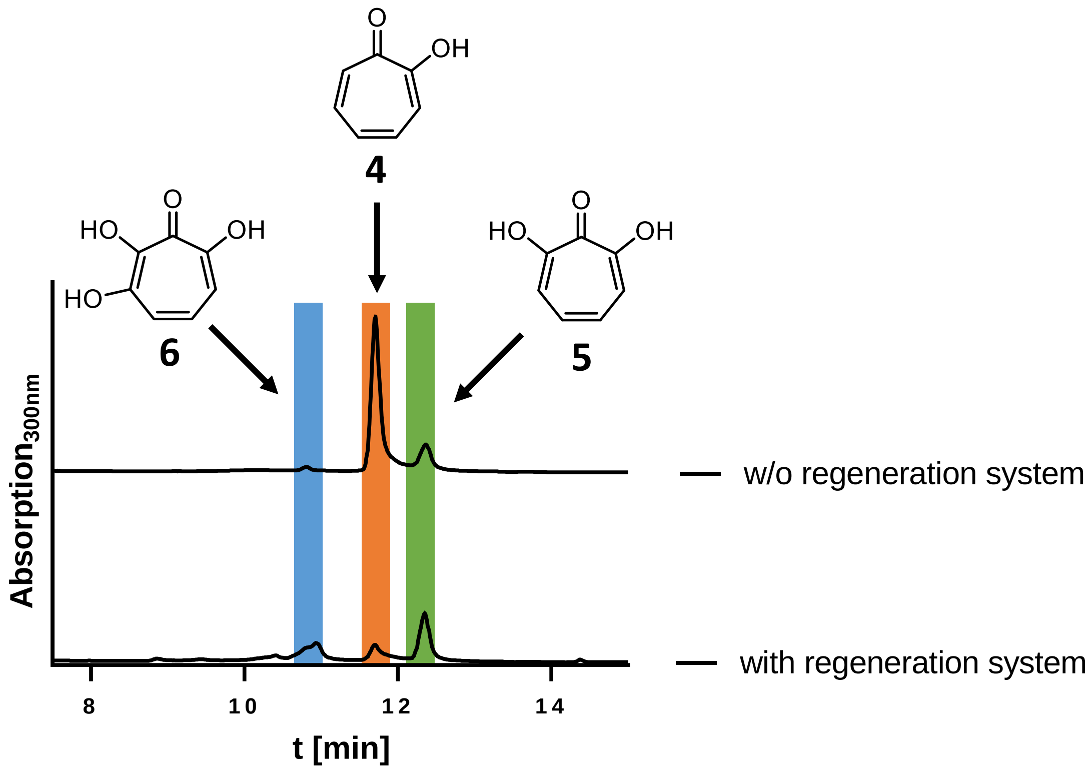

Figure S34 Comparison of conversion of tropolone (4) by TrlCD with and without cofactor regeneration system. In both assays, TrlCD +FAD were incubated for 2h at 30°C with 4, which was converted into **5** and **6** (see method section). One assay additionally contained the sodium formate NADH regeneration system, while the other one was only supplemented with NADH. Samples were measured with the HPLC program for the analytical SunFire column (see above in method section, 1.19 LC-MS analysis).

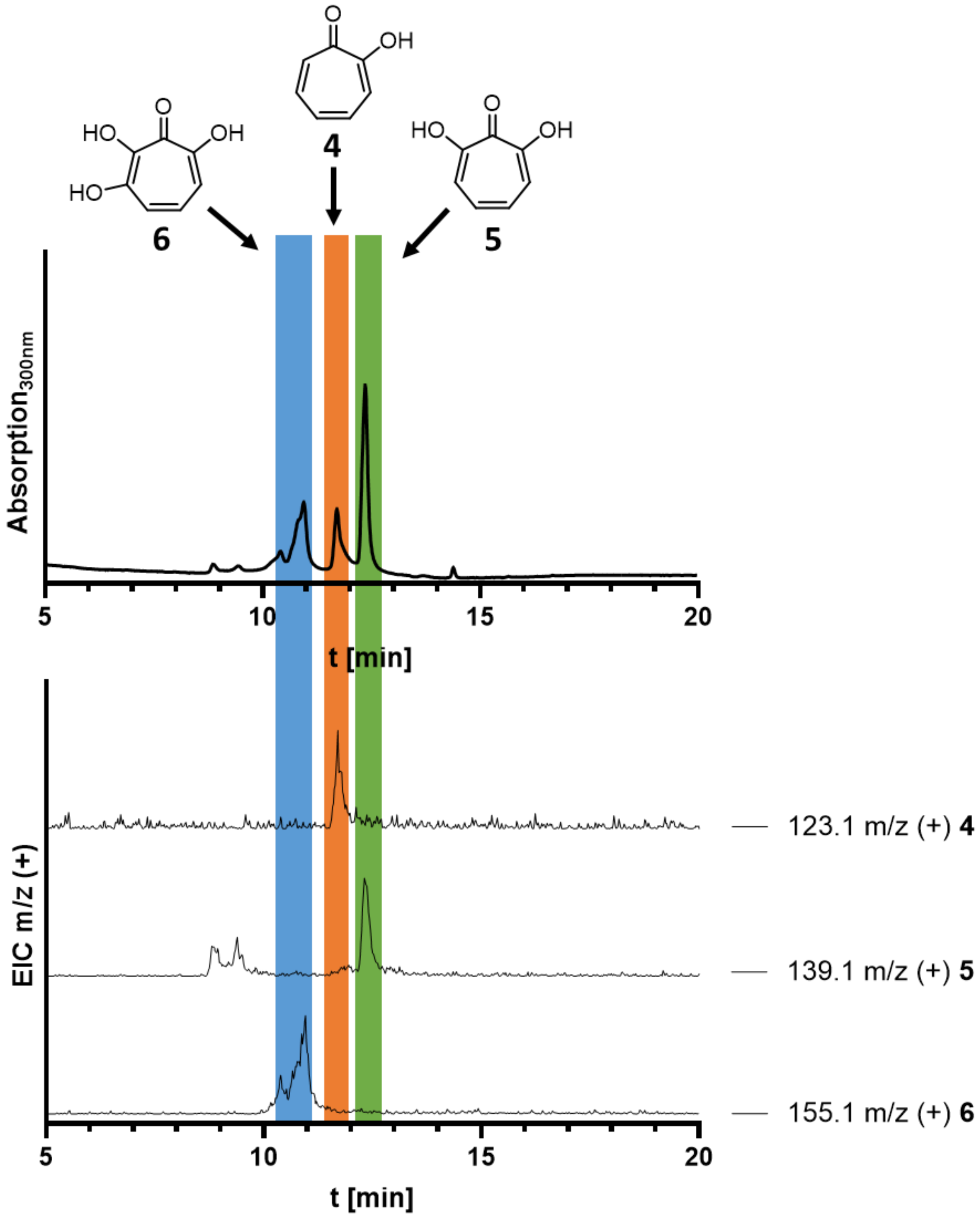

Figure S35 EIC of compounds 4, 5 and 6 obtained during enzymatic assays with TrlCD. Masses were measured on a low resolution LC-MS system in positive mode and theoretical masses for the different compounds were extracted. Samples were measured with the HPLC program for the analytical SunFire column (see above in method section, 1.19 LC-MS analysis).

7-Hydroxy-tropolone (**5**)

3,7-Dihydroxy-tropolone (**6**)

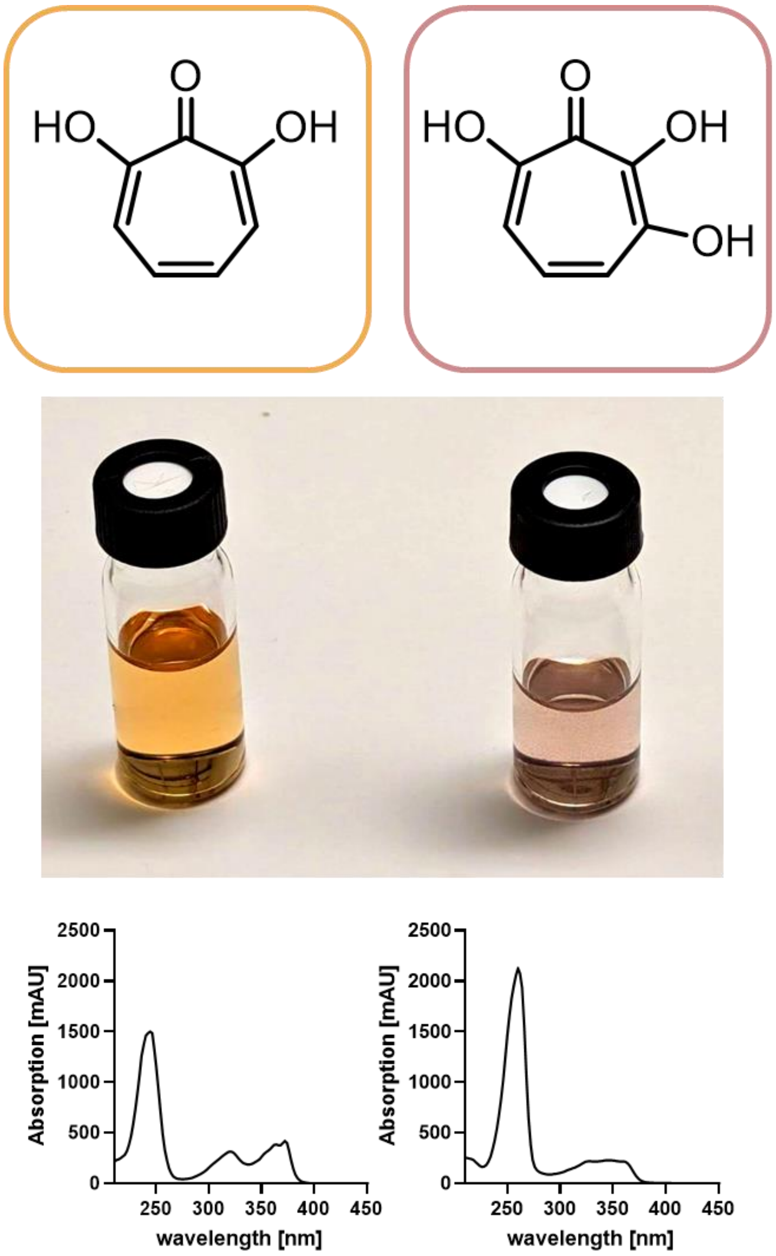

Figure S36 Overview of structures, methanol solutions and UV spectra of compounds 5 and 6

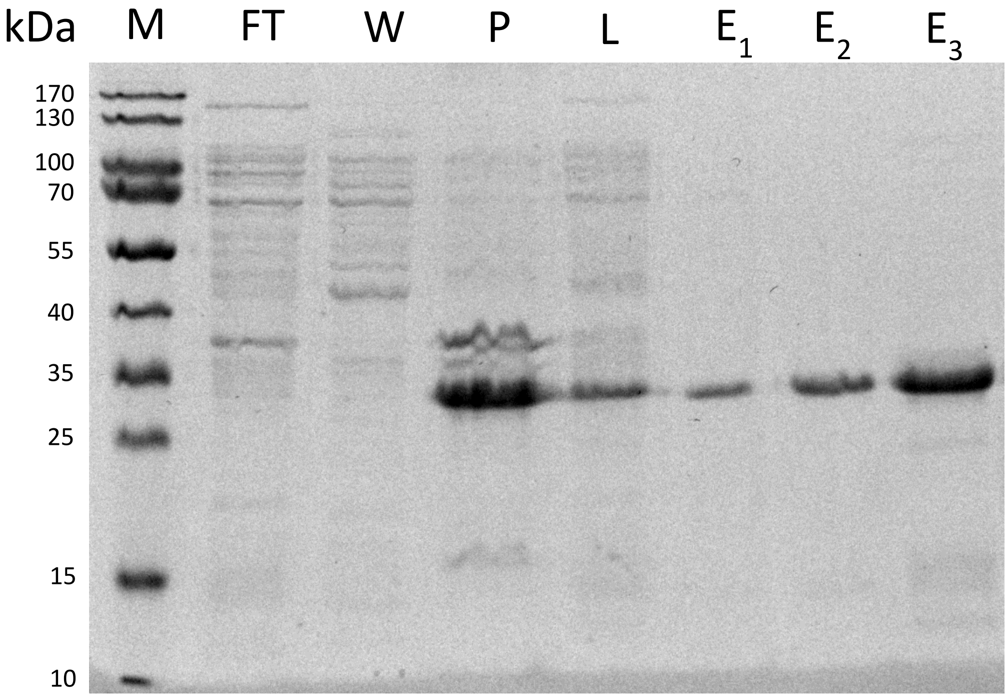

Figure S37 SDS-PAGE analysis of different fractions collected during affinity purification (IMAC) of 6xHis-gb1-tagged TrlA. M: Marker, PageRuler Prestained protein ladder (ThermoScientific), **FT**: column flow-through of the affinity purification, **W**: wash fraction, **P**: cell pellet after lysis, **L**: cleared cell lysate, **E_1-E_3**: elution fractions 1-3

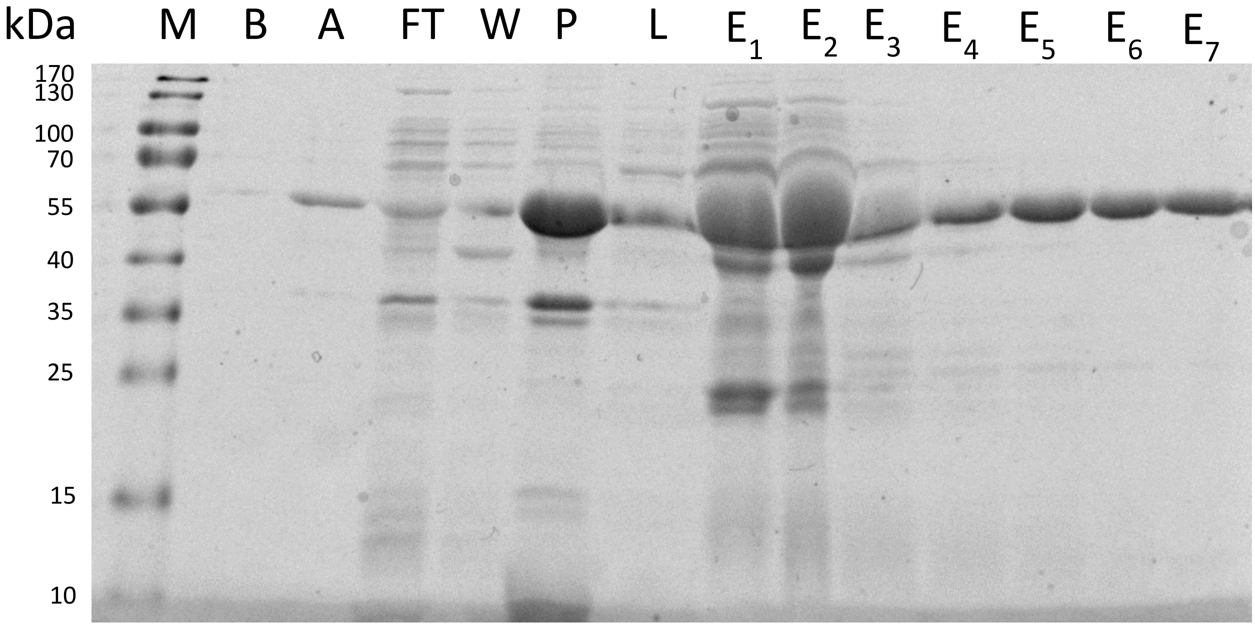

Figure S38 SDS-PAGE analysis of different fractions collected during affinity purification (IMAC) of 6xHis-tagged TrlC. M: Marker, PageRuler Prestained protein ladder (ThermoScientific), **B**: cell culture before induction with IPTG, **A**: cell culture after induction with IPTG, **FT**: column flow-through of the affinity purification, **W**: wash fraction, **P**: cell pellet after lysis, **L**: cleared cell lysate, **E_1-E_7**: elution fractions 1-7

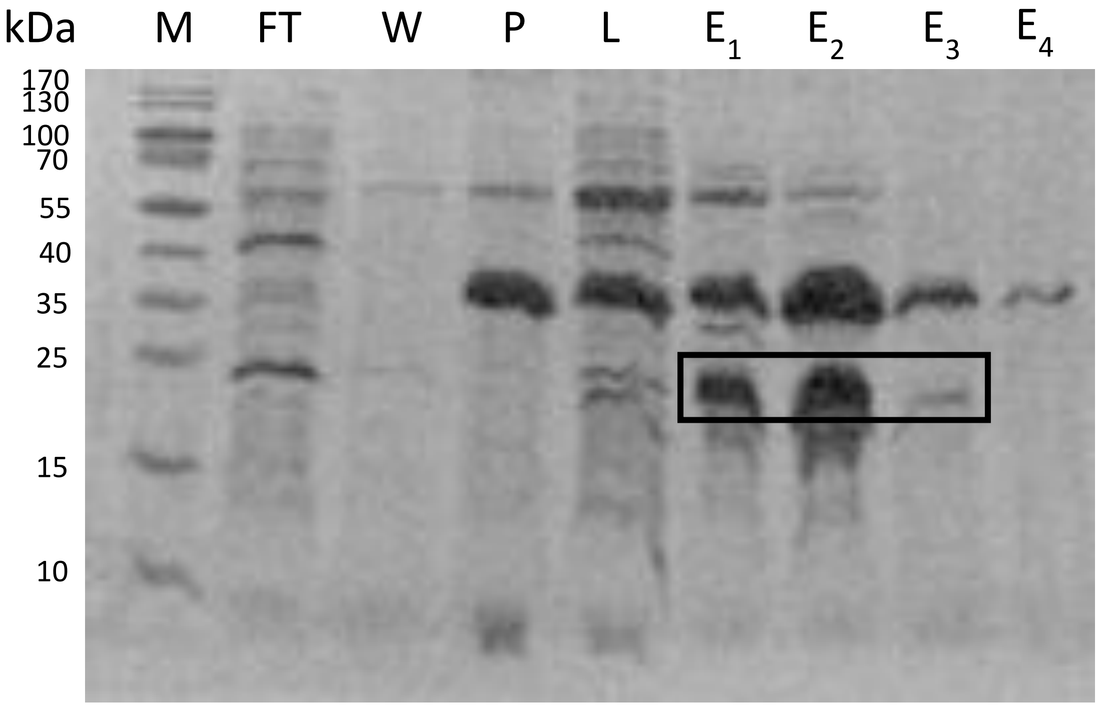

Figure S39 SDS-PAGE analysis of different fractions collected during affinity purification (IMAC) of 6xHis-gb1-tagged TrlD. **M**: Marker, PageRuler Prestained protein ladder (ThermoScientific), **FT**: column flow-through of the affinity purification, **W**: wash fraction, **P**: cell pellet after lysis, **L**: cleared cell lysate, **E_1-E_4**: elution fractions 1-4. TrlD is highlighted with the black box, other bands are from the overexpression of chaperones encoded in the producing strain.

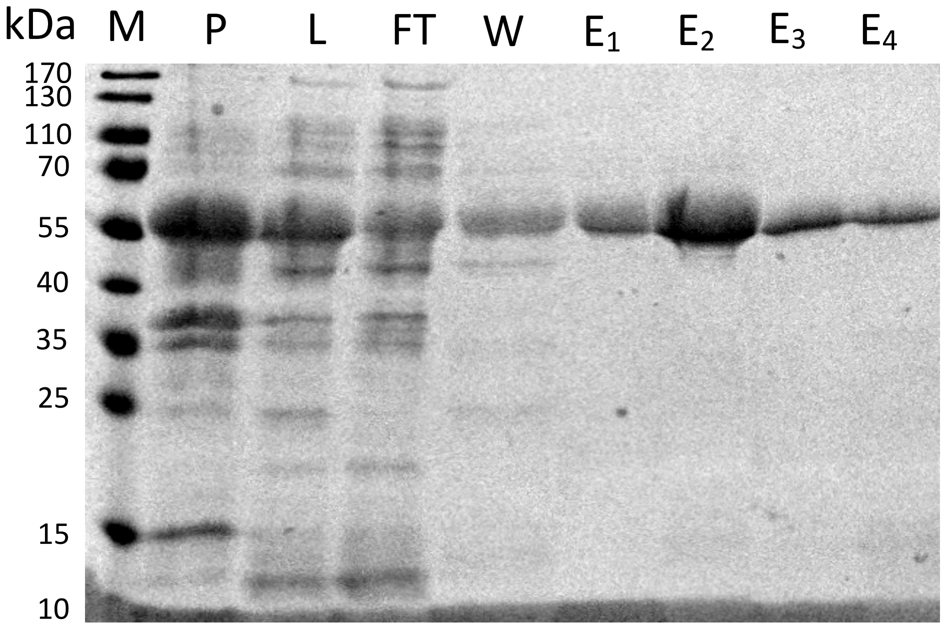

Figure S40 SDS-PAGE analysis of different fractions collected during affinity purification (IMAC) of 6xHis-gb1-tagged TrlE. M: Marker, PageRuler Prestained protein ladder (ThermoScientific), **FT**: column flow-through of the affinity purification, **W**: wash fraction, **P**: cell pellet after lysis, **L**: cleared cell lysate, **E_1-E_4**: elution fractions 1-4

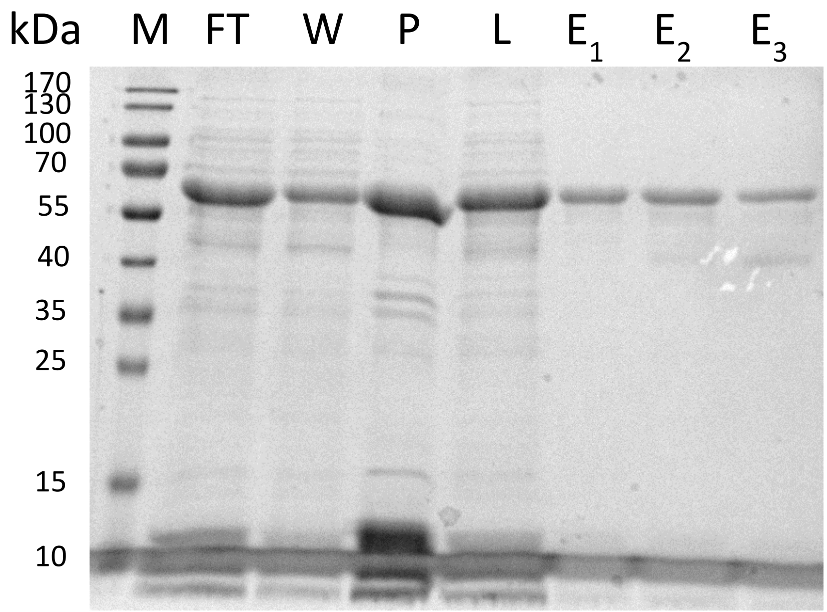

Figure S41 SDS-PAGE analysis of different fractions collected during affinity purification (MBP-Trap) of MBP-tagged TrlF. M: Marker, PageRuler Prestained protein ladder (ThermoScientific), **FT**: column flow-through of the affinity purification, **W**: wash fraction, **P**: cell pellet after lysis, **L**: cleared cell lysate, **E_1-E_4**: elution fractions 1-4

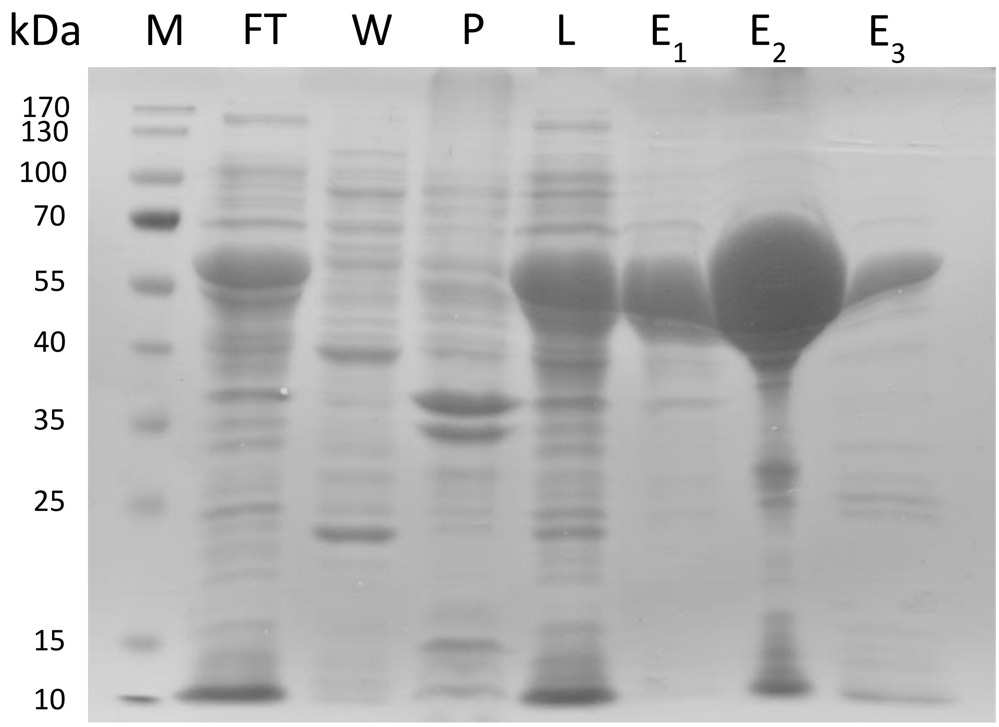

Figure S42 SDS-PAGE analysis of different fractions collected during affinity purification (IMAC) of 6xHis-gb1-tagged TrlE H213A. M: Marker, PageRuler Prestained protein ladder (ThermoScientific), **FT**: column flow-through of the affinity purification, **W**: wash fraction, **P**: cell pellet after lysis, **L**: cleared cell lysate, **E_1-E_3**: elution fractions 1-3

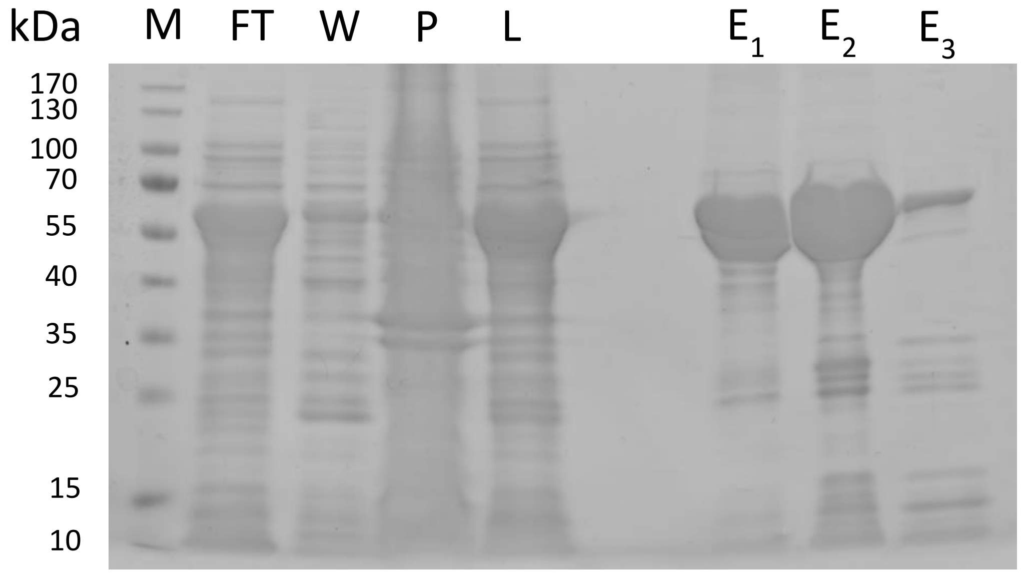

Figure S43 SDS-PAGE analysis of different fractions collected during affinity purification (IMAC) of 6xHis-gb1-tagged TrlE H213E. M: Marker, PageRuler Prestained protein ladder (ThermoScientific), **FT:** column flow-through of the affinity purification, W: wash fraction, **P:** cell pellet after lysis, **L:** cleared cell lysate, **E_1-E_3:** elution fractions 1-3

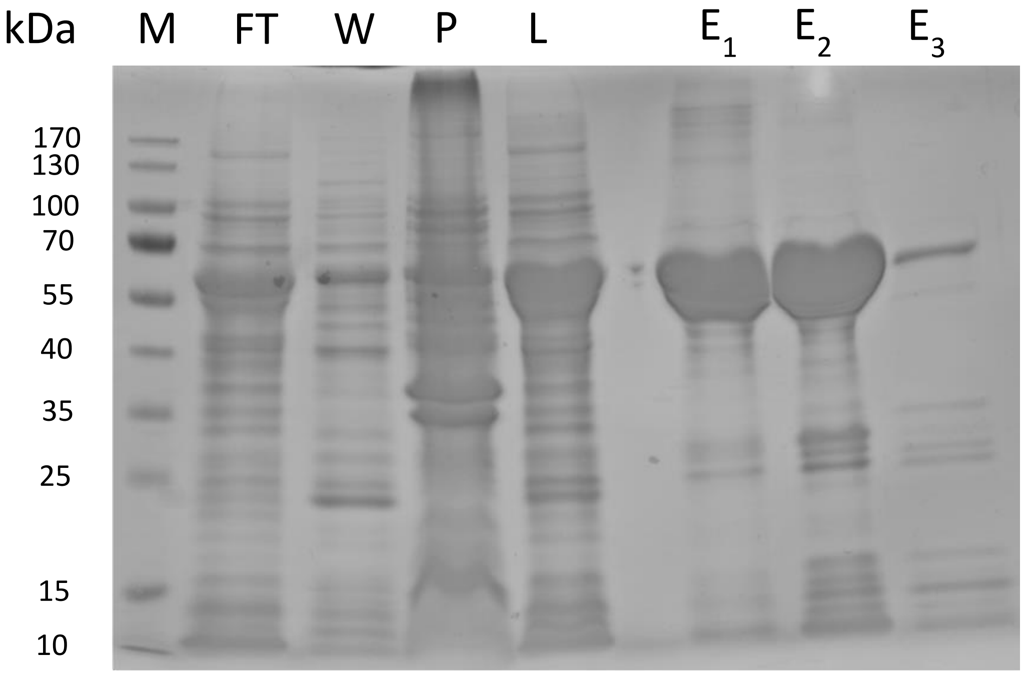

Figure S44 SDS-PAGE analysis of different fractions collected during affinity purification (IMAC) of 6xHis-gb1-tagged TrlE H213Q. M: Marker, PageRuler Prestained protein ladder (ThermoScientific), **FT**: column flow-through of the affinity purification, **W**: wash fraction, **P**: cell pellet after lysis, **L**: cleared cell lysate, **E_1-E_3**: elution fractions 1-3

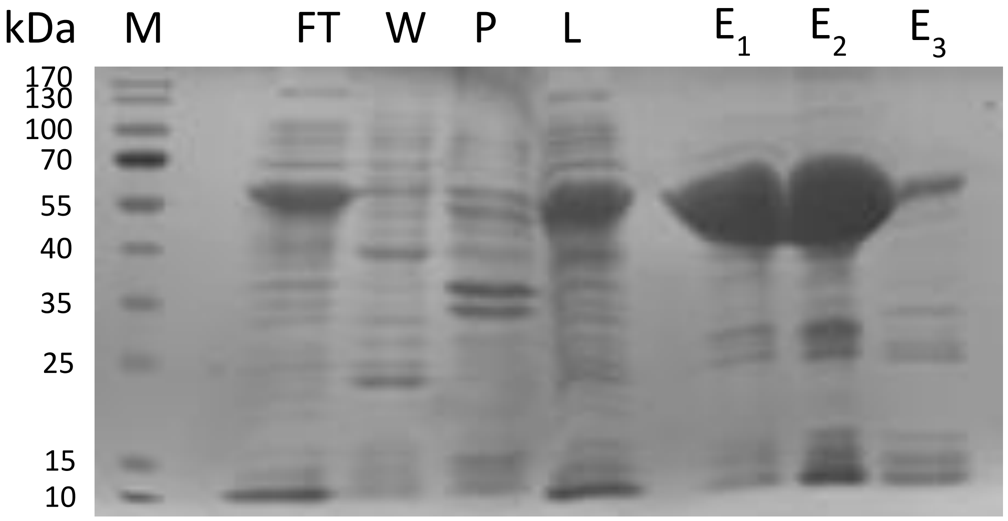

Figure S45 SDS-PAGE analysis of different fractions collected during affinity purification (IMAC) of 6xHis-gb1-tagged TrlE Y217F. M: Marker, PageRuler Prestained protein ladder (ThermoScientific), **FT**: column flow-through of the affinity purification, **W**: wash fraction, **P**: cell pellet after lysis, **L**: cleared cell lysate, **E_1-E_3**: elution fractions 1-3

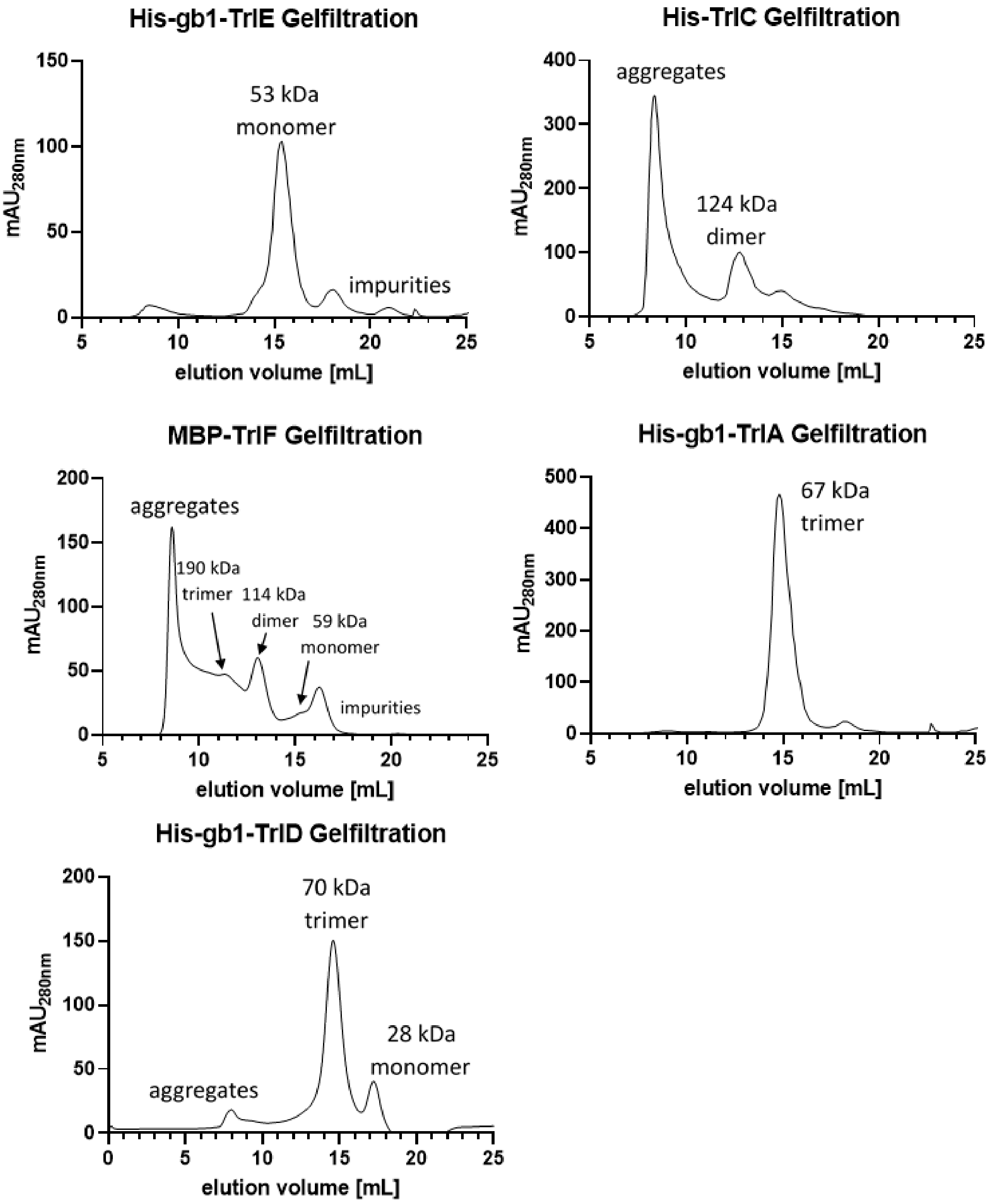

Figure S46 Analytical SEC of his-gb1 tagged TrlE, TrlA and TrlD, his tagged TrlC and MBP tagged TrlF.

3. SI References

1. L. Betancor, M. J. Fernandez, K. J. Weissman and P. F. Leadlay, *Chembiochem*, 2008, **9**, 2962-2966.
2. R. Teufel, V. Mascaraque, W. Ismail, M. Voss, J. Perera, W. Eisenreich, W. Haehnel and G. Fuchs, *Proc. Natl. Acad. Sci. U S A*, 2010, **107**, 14390-14395.
3. R. Teufel, C. Gantert, M. Voss, W. Eisenreich, W. Haehnel and G. Fuchs, *J. Biol. Chem.*, 2011, **286**, 11021-11034.
4. R. Teufel, T. Friedrich and G. Fuchs, *Nature*, 2012, **483**, 359-362.
5. M. van den Belt, C. Gilchrist, T. J. Booth, Y. H. Chooi, M. H. Medema and M. Alanjary, *BMC Bioinformatics*, 2023, **24**, 181.
6. K. Tamura, G. Stecher and S. Kumar, *Mol. Biol. Evol.*, 2021, **38**, 3022-3027.
7. W. Kabsch, *Acta Crystallogr. D Biol. Crystallogr.*, 2010, **66**, 125-132.
8. P. Evans, *Acta Crystallogr. D Biol. Crystallogr.*, 2006, **62**, 72-82.
9. A. J. Mccoy, R. W. Grosse-Kunstleve, P. D. Adams, M. D. Winn, L. C. Storoni and R. J. Read, *J. Appl. Crystallogr.*, 2007, **40**, 658-674.
10. J. Jumper, R. Evans, A. Pritzel, T. Green, M. Figurnov, O. Ronneberger, K. Tunyasuvunakool, R. Bates, A. Zídek, A. Potapenko, A. Bridgland, C. Meyer, S. A. A. Kohl, A. J. Ballard, A. Cowie, B. Romera-Paredes, S. Nikolov, R. Jain, J. Adler, T. Back, S. Petersen, D. Reiman, E. Clancy, M. Zielinski, M. Steinegger, M. Pacholska, T. Berghammer, S. Bodenstein, D. Silver, O. Vinyals, A. W. Senior, K. Kavukcuoglu, P. Kohli and D. Hassabis, *Nature*, 2021, **596**, 583-+.
11. P. Emsley and K. Cowtan, *Acta Crystallogr. D Biol. Crystallogr.*, 2004, **60**, 2126-2132.
12. P. D. Adams, R. W. Grosse-Kunstleve, L. W. Hung, T. R. Ioerger, A. J. McCoy, N. W. Moriarty, R. J. Read, J. C. Sacchettini, N. K. Sauter and T. C. Terwilliger, *Acta Crystallogr. D Biol. Crystallogr.*, 2002, **58**, 1948-1954.
13. G. N. Murshudov, P. Skubák, A. A. Lebedev, N. S. Pannu, R. A. Steiner, R. A. Nicholls, M. D. Winn, F. Long and A. A. Vagin, *Acta Crystallogr. D Biol. Crystallogr.*, 2011, **67**, 355-367.
14. V. B. Chen, W. B. Arendall, J. J. Headd, D. A. Keedy, R. M. Immormino, G. J. Kapral, L. W. Murray, J. S. Richardson and D. C. Richardson, *Acta Crystallogr. D Biol. Crystallogr.*, 2010, **66**, 12-21.
15. D. Schachter and J. V. Taggart, *J. Biol. Chem.*, 1953, **203**, 925-934.
